# Data for in-situ industrial site characterization with the applications of combined subsurface and surface mapping

**DOI:** 10.1016/j.dib.2018.04.119

**Published:** 2018-05-02

**Authors:** Mohd Hariri Arifin, John Stephen Kayode, Muhammad Azrief Azahar, Habibah Jamil, Saznira Fadila Ahmad Sabri

**Affiliations:** a School of Environmental Science and Natural Resources, Department of Geology, National University of Malaysia, Malaysia; b Environmental Technology, School of Industrial Technology, Universiti Sains Malaysia, 11800 Pulau-Pinang, Malaysia; c In Infra Tech Geo Solutions, Unit: 840, 8th Floor, Block A, Lobby C, Complex Kelana Centre Point, No. 3, Jalan SS7/19, 47301 Petaling Jaya, Malaysia

## Abstract

The paper presents the data from the surface and subsurface mapping of this area for the purpose of siting industrial city in the area. The field data collected combine with the borehole data was to successfully apply these to solving geological, environmental and engineering complications posed by the complexity of the subsurface geological structures underlain this area. The Electrical Resistivity, (ER) and Induced Polarization, (IP) data were initially processed using RES2DINV software model to generate the depth to the lithological units together with topographic correction. The 2-D ER and IP data were collected from 23rd April 2017 up until 7th May 2017 covering a total of about 17.6 km along 44 survey lines using ABEM Terrameter SAS4000 for the field measurement. A total of 20 Borehole logs data were recorded to better characterized in-situ, the subsurface geological formations emplaced in the study area. The study area is located at Bagan Datuk, Perak Darul Ridzuan situated on Latitude 2° 44.653'N and Longitudes 104° 28.79' E along the west coast Peninsula Malaysia. The topography of the area is generally flat low–laying and elevation range from about 0 m to 32 m above mean sea level (MSL).

**Specifications table**

**Table:** 

Subject area	*Engineering, Geophysics and Geology*
More specific subject area	*Electrical Resistivity and Borehole Engineering*
Type of data	*Table, image, text file, figure*
How data was acquired	*The 2D ER and IP data were acquired using Land imaging system by Survey with ABEM Terrameter SAS4000, and Rig YWE D-90R using Rotary wash method to bore and auto record the borehole logs.*
Data format	*Raw, filtered, analyzed*
Experimental factors	*The 2D ER and IP data were originally processed using RES2DINV software model. The borehole data directly characterized the subsurface geologic formations in-situ.*
Experimental features	*Very brief experimental description*
Data source location	*Bagan Datuk, Perak Darul Ridzuan, Malaysia, Latitudes 2° 44.653′N and Longitudes 104° 28.79′E*
Data accessibility	*The data is with this article.*

**Value of the data**

• The data in this research is valuable for industrial project of this magnitude as it helps in the design of the super structural facilities envisioned for the industrial city.
• The data stands as vital tools in project planning and designing of suitable foundations for the industrial and city structures to promote best safety practice.
• The data being reported is exceptionally relevant in policy formulations, assessment and prudential project management that could be implemented in any part of the World.
• The data help in smart decisions and prioritizing the type of edifices to be sited at a specific location because the study area serves as an interphase between the coastal sediments and the basement structures.
• The data is to be used by the building, construction and geotechnical companies as a bench mark in the determinations of the integrity of subsurface structures and the type of stabilization to be adopted.

## 1 Data

**Fig. 1:**
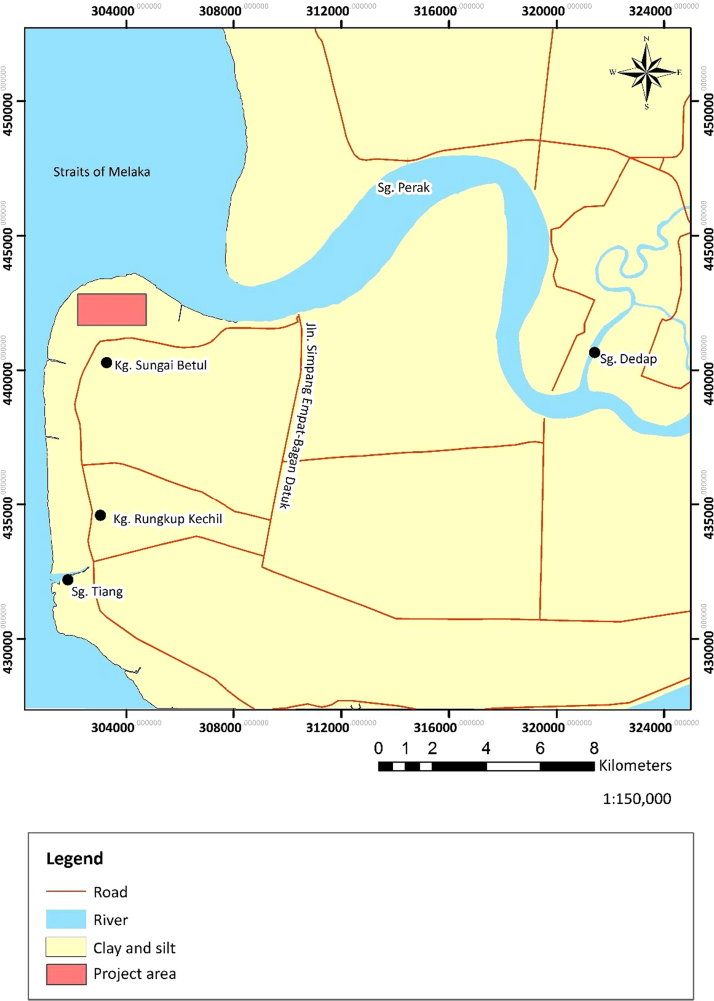
Geological map of Bagan Datuk, Perak Darul Ridzuan, Malaysia showing the data collection location.

Geological settings of the project area where the data was acquired is mostly quaternary deposits of Gula Formation that consist of recent alluvium of marine facies. The Quaternary sediments in the Bagan Datuk area are said to be Holocene marine deposits, which consist mostly of clay and plant remains deposited in a mangrove environment as shown in Fig. 1. The subsurface geological structures are made up of peat with some minor gravel, silty sands and clayey sands. The Bagan Datuk member represents a shallow marine and estuarine deposits that underlain the Port Weld Member [1].

Electrical Resistivity (ER) and IP methods of geophysical survey greatly depends on the electrolytic conduction as the common mechanism where the current injected in the ground flow via the movement of ions in subsurface fluids. Although the two methods are widely used for groundwater exploration, however, the ER method is useful in the in-situ characterization of the subsurface, if combine with the borehole logs. In most geologic environments, the resistivity of these rocks greatly dependent on the degree of fracturing and the percentage of these fractures filled with fluids [2].

**Fig. 2:**
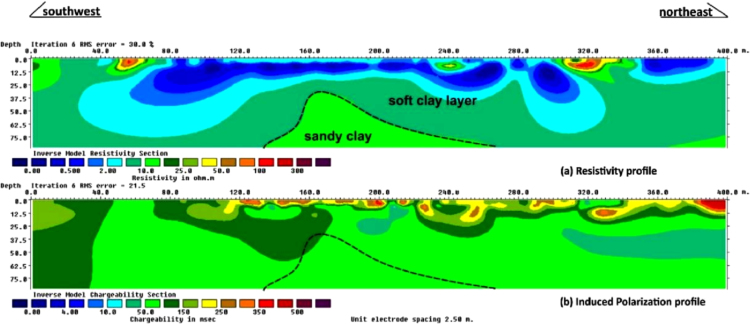
A typical sample of the ER and IP data collected along the survey line 1.

Rock sediments rich in Clay contents have higher chargeability values recorded than sediments that are principally composed of sands. However, several factors impact the chargeability values of rocks. Typically, the chargeability values depend on concentration of clay or metallic particles present in rocks. The higher the chargeability, the lower the electrical conductivity, hence, the chargeability increases with decreasing porosity [2]. A typical sample of the data collected is as shown in Fig. 2. The 20 Borehole logs comprises of 60 data sheets are attached as Appendix A.

## 2 Experimental design

**Fig. 3:**
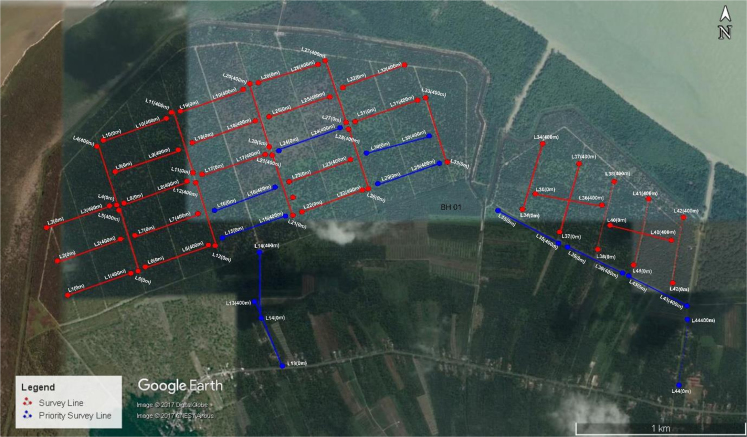
Experimental design and location of the data collection area in Bagan Datuk, Perak Darul Ridzuan, Malaysia.

The survey was carried out using 61 electrodes, connected to the multi-core cable reals. A resistivity meter for land imaging system with internal microprocessor (ABEM Terrameter SAS4000) controlled the circuitry together with an electronic switching unit (ABEM LUND ES 10 – 64) used to automatically select the relevant four electrodes at a time for each data point (Fig. 3).

## 3 Materials and methods

The Electrical resistivity, (ER) data are to define the subsurface geological structures, while the induced polarization (IP) data are to delineate the concentrations and distributions of the clayey contents of the subsurface strata. The ER data give values that ranged from 0 Ω-m to 1000 Ω-m (Fig. 2a). In the same vein, the IP Chargeability data showed large chargeability values from 0 ms to 800 ms as shown in Fig. 2b.
