# Supplementary material for: Data for in-situ industrial site characterization with the applications of combined subsurface and surface mapping
Source: Data Brief. 2018 May 2;18:1864–8. doi: 10.1016/j.dib.2018.04.119 (PMC5999011; doi:10.1016/j.dib.2018.04.119)
Supplement: Supplementary file 2 — Supplementary material [file mmc2.pdf]

APPENDIX A

BOREHOLE LOGS

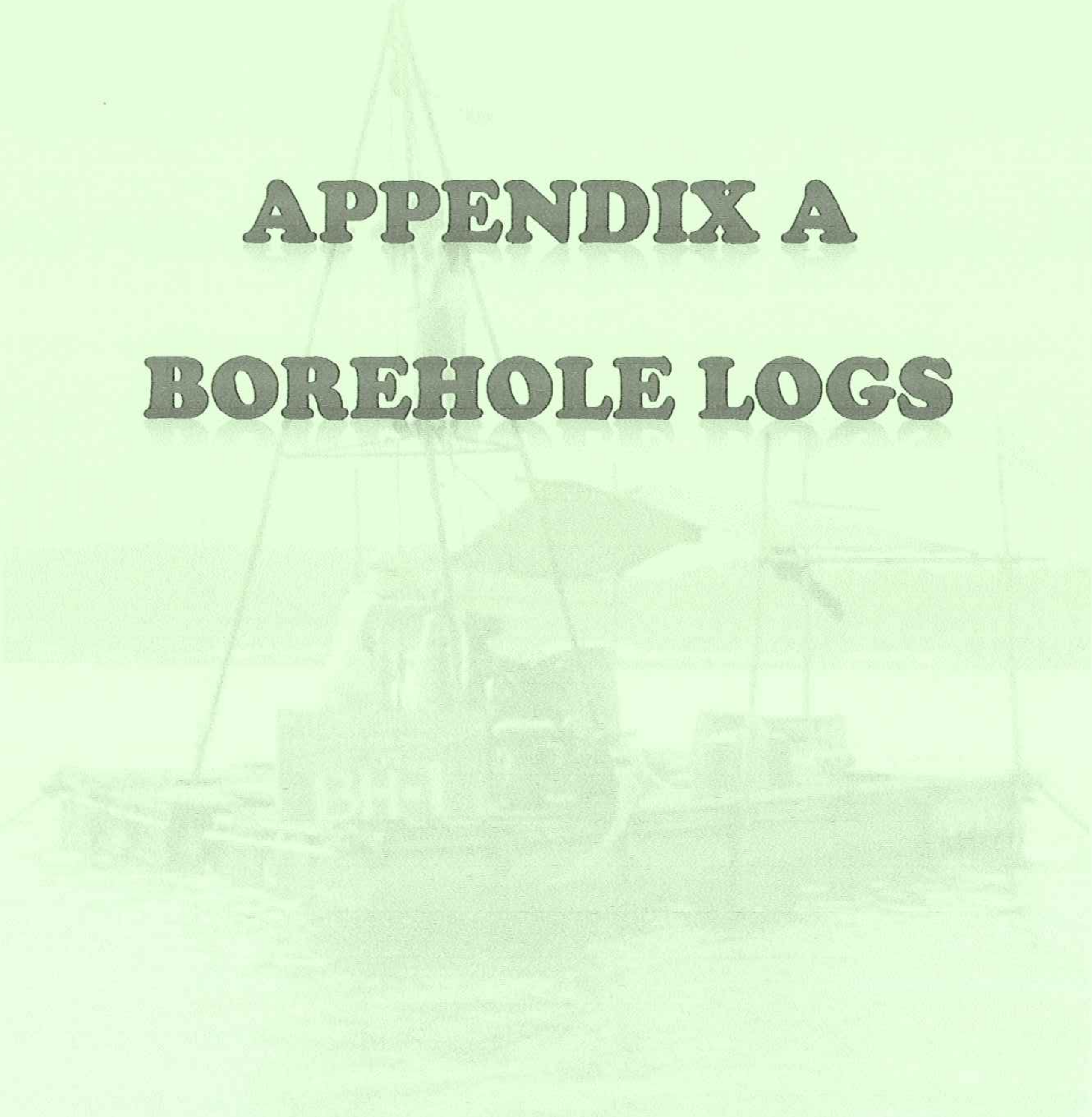

ENGINEERING BOREHOLE LOG

Project :	SOIL INVESTIGATION WORKS		Borehole No : **BH 1**
Location :	AT BAGAN DATUK WATER CITY PHASE 1, PERAK DARUL RIDZUAN		Ground Level: 0.429 m
Client :	PERBADANAN KEMAJUAN NEGERI PERAK	Rig Type : YWE D-90R	Supervisor: SHAH
Consultant:	INFRA TECH GEO SOLUTIONS (M) SDN. BHD.	Drill Method: Rotary Wash	REDZA
Maincon :	INFRA TECH PROJECTS MALAYSIA SDN. BHD.	Casing Type : NW	Water Level : 0.35 m
			Date Start : 11.05.2017
			Date Finish : 13.05.2017

Depth m	Strata Thick-ness	Description of Strata	Log	Sample No	Depth m	Rec Ratio	75	75	75	75	75	75	For <75	N Value	For <300
0															
	0.30	Top soil.		D	0.300										
1.5		Very soft dark grey CLAY.	---	P1/D1	1.500<br>1.950	100%	0	0	0	0	0	0		0	
3		Very soft dark grey CLAY with some organic material (seashell).	---	P2/D2	3.000<br>3.450	100%	0	0	0	0	0	0		0	
4.5		Very soft dark grey CLAY with some organic material (seashell).	---	P3/D3	4.500<br>4.950	100%	0	0	0	0	0	0		0	
5		Dark grey CLAY of high plasticitv.	---	UD 1	5.000<br>5.500	100%									
6		Very soft dark grey CLAY with some organic material (seashell).	---	P4/D4	6.000<br>6.450	100%	0	0	0	0	0	0		0	
7.5		Very soft dark grey CLAY.	---	P5/D5	7.500<br>7.950	100%	0	0	0	0	0	0		0	
8.5			---	UD 2	8.500	100%									
9		Very soft dark grey silty CLAY.	---	P6/D6	9.000<br>9.450	100%	0	0	0	0	0	0		0	
10.5		Very soft dark grey CLAY.	---	P7/D7	10.500<br>10.950	100%	0	0	0	0	0	0		0	
12		Very soft dark grey CLAY.	---	P8/D8	12.000<br>12.450	100%	1	0	0	0	0	0		1	
12.5		Dark grey CLAY of high plasticity.	---	UD 3	12.500<br>13.000	100%									
13.5		No recovery.		P9	13.500<br>13.950	0%	0	0	0	0	0	0		0	
15		Very soft dark grey CLAY.	---	P10/D9	15.000<br>15.450	100%	0	1	0	0	0	0		1	
16.5		Very soft dark grey CLAY with lenses of fine grain sand.	---	P11/D10	16.500<br>16.950	100%	1	1	0	0	0	0		2	
17			---	UD 4	17.000<br>17.500	100%									
18		Very soft dark grey CLAY with decayed wood and lenses of fine to medium sand.	---	P12/D11	18.000<br>18.450	100%	0	0	0	0	0	0		0	
19.5		Very soft dark grey CLAY with some organic material.	---	P13/D12	19.500<br>19.950	100%	0	1	0	1	0	0		1	
20															

Rec = Recovery

SPT PLOT

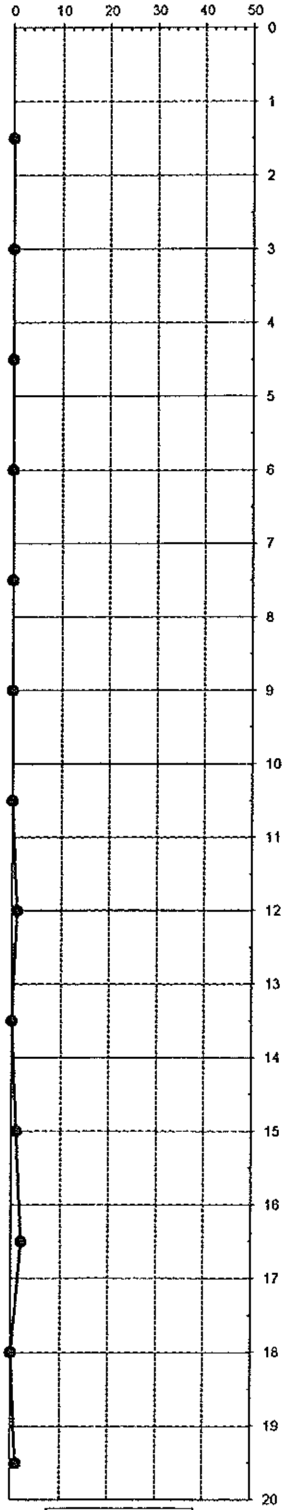

SPT PLOT

Legend:

- D — Disturbed Sample
- P — Standard Penetration Test
- UD — Undisturbed Sample
- MZ — Mazier Sample
- VS — Vane Shear Test
- C — Rock Coring
- W — Water Sample
- N - No. of Blows/300mm

HSD ENGINEERING SERVICES

NOTE :

** Existing ground leve

Example:
50 | 120 = 50 Blows/120 mm

Cohesive Soil (N): 0, 2, 4, 8, 15, 30 — V.Soft, Soft, Firm, Stiff, V.Stiff, Hard

Non-cohesive Soil (N): 0, 4, 10, 30, 50 — V.Loose, Loose, Med Dense, Dense, V.Dense

ENGINEERING BOREHOLE LOG

Project : SOIL INVESTIGATION WORKS			Borehole No : BH 1
Location : AT BAGAN DATUK WATER CITY PHASE 1, PERAK DARUL RIDZUAN			Ground Level: 0.429 m
Client : PERBADANAN KEMAJUAN NEGERI PERAK	Rig Type : YWE D-90R	Driller : SHAH	Water Level : 0.35 m
Consultant: INFRA TECH GEO SOLUTIONS (M) SDN. BHD.	Drill Method: Rotary Wash	Supervisor: REDZA	Date Start : 11.05.2017
Job No. : INFRA TECH PROJECTS MALAYSIA SDN. BHD.	Casing Type : NW		Date Finish : 13.05.2017

Depth m	Strata Thickness	Description of Strata	Log	Sample No	Depth m	Rec Ratio	75	75	75	75	75	75	For <75	N Value	For <300
20		Dark grey CLAY of high plasticity.		UD 5	20.000 – 20.500	100%									
21		Very soft dark grey CLAY with some organic material.		P14/D13	21.000 – 21.450	100%	0	0	0	0	0	1		1	
22–23		Very soft dark grey CLAY with some organic material.		P15/D14	22.500 – 22.950	100%	0	0	0	0	0	0		0	
24		Very soft dark grey CLAY with some organic material.		P16/D15	24.000 – 24.450	100%	0	0	0	0	0	0		0	
25–26		Very soft dark grey CLAY with some organic material.		P17/D16	25.500 – 25.950	100%	0	0	0	0	0	0		0	
27		Very soft dark grey CLAY with some organic material.		P18/D17	27.000 – 27.450	100%	0	0	0	0	0	0		0	
28–29		Very soft dark grey CLAY with some organic material.		P19/D18	28.500 – 28.950	100%	0	0	0	0	0	0		0	
30–31	31.50	Very soft dark grey CLAY.		P20/D19	30.000 – 30.450	100%	0	0	0	0	0	0		0	
31.5–32		Soft dark grey silty CLAY with some organic material.		P21/D20	31.500 – 31.950	100%	0	0	1	1	0	1		3	
33		Firm dark grey silty CLAY with some organic material.		P22/D21	33.000 – 33.450	100%	0	1	1	2	1	1		5	
34–35		Stiff light silty CLAY contact with organic shell (10 cm).		P23/D22	34.500 – 34.950	100%	1	1	2	2	4	3		11	
36		Very soft to soft light grey silty CLAY.		P24/D23	36.000 – 36.450	100%	0	0	1	0	1	0		2	
37–38		Stiff light grey CLAY with lenses of fine to medium sand.		P25/D24	37.500 – 37.950	100%	0	0	1	2	3	3		9	
39–40		Stiff light grey silty CLAY with decayed wood.		P26/D25	39.000 – 39.450	100%	0	1	3	2	2	3		10	

Sampling Detail; Penetration, P (mm) – SPT BLOW COUNT. Rec = Recovery

SPT PLOT

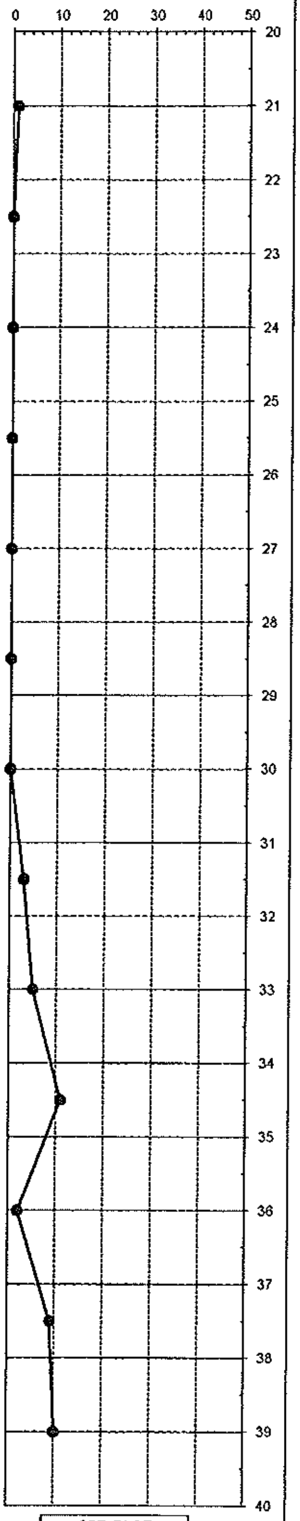

Legend:

- D – Disturbed Sample
- P – Standard Penetration Test
- UD – Undisturbed Sample
- MZ – Mazier Sample
- VS – Vane Shear Test
- C – Rock Coring
- W – Water Sample
- N - No. of Blows/300mm

NOTE :

** Existing ground level

Example: 50 | 120 = 50 Blows/120 mm

Cohesive Soil (N)	0	2	4	8	15	30	
	V.Soft	Soft	Firm	Stiff	V.Stiff	Hard	
Non-cohesive Soil (N)	0	4	10	30	50		
	V.Loose	Loose	Med Dense	Dense	V.Dense		

HSD ENGINEERING SERVICES

ENGINEERING BOREHOLE LOG

Project : SOIL INVESTIGATION WORKS			Borehole No : **BH 1**
Location : AT BAGAN DATUK WATER CITY PHASE 1, PERAK DARUL RIDZUAN			Ground Level: 0.429 m
Client : PERBADANAN KEMAJUAN NEGERI PERAK	Rig Type : YWE D-90R	Driller : SHAH	Water Level : 0.35 m
Consultant: INFRA TECH GEO SOLUTIONS (M) SDN. BHD.	Drill Method: Rotary Wash	Supervisor: REDZA	Date Start : 11.05.2017
Job No. : INFRA TECH PROJECTS MALAYSIA SDN. BHD.	Casing Type : NW		Date Finish : 13.05.2017

Depth m	Strata Thickness	Description of Strata	Log	SAMPLING DETAIL Sample No	Depth m	Rec Ratio	Penetration, P (mm) 75	75	75	75	75	75	For <75	N Value	For <300
							SPT BLOW COUNT								
40															
41	42.00	Medium dense grey slightly silty SAND.		P27/D26	40.500 40.950	22%	3	5	5	5	7	5		22	
42 – 43		Dense light grey fine SAND.		P28/D27	42.000 42.450	9%	3	5	7	10	9	10		36	
44		Medium dense light grey SAND.		P29/D28	43.500 43.950	13%	2	5	5	4	5	8		22	
45	45.45	Firm light grey silty CLAY with some organic material.		P30/D29	45.000 45.450	71%	0	1	0	3	2	2		7	
46		End of BH 1 at 45.45 m depth.													
47 – 60															

Rec = Recovery

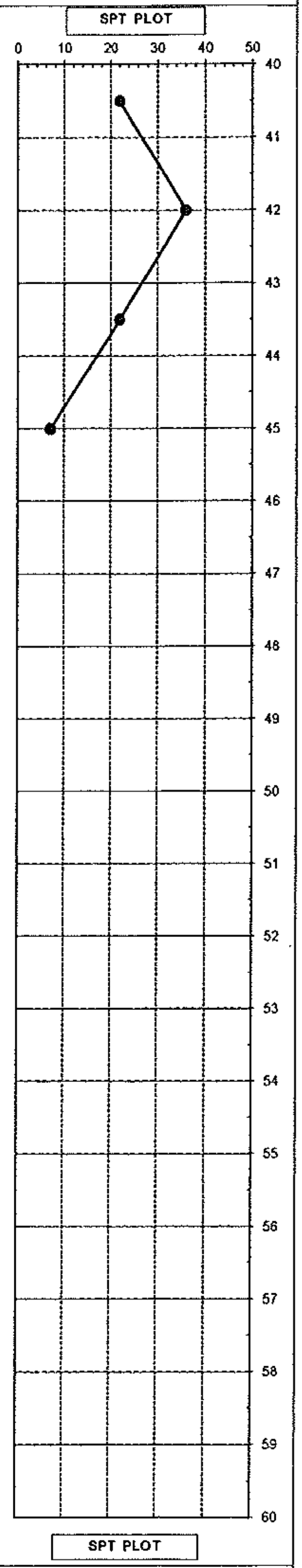

Legend:

- D Disturbed Sample
- P Standard Penetration Test
- UD Undisturbed Sample
- MZ Mazier Sample
- VS Vane Shear Test
- C Rock Coring
- W Water Sample
- N · No. of Blows/300mm

NOTE :

** Existing ground level

Example:

50 | 120 = 50 Blows/120 mm

Cohesive Soil (N): 0, 2, 4, 8, 15, 30 — V.Soft, Soft, Firm, Stiff, V.Stiff, Hard

Non-cohesive Soil (N): 0, 4, 10, 30, 50 — V.Loose, Loose, Med Dense, Dense, V.Dense

HSD ENGINEERING SERVICES

ENGINEERING BOREHOLE LOG

Project : SOIL INVESTIGATION WORKS			Borehole No : BH 2
Location : BAGAN DATUK WATER CITY PHASE 1, PERAK DARUL RIDZUAN			Ground Level: 0.252 m
Client : PERBADANAN KEMAJUAN NEGERI PERAK	Rig Type : YWE D-90R	Driller : SHAH	Water Level : 0.42 m
Consultant: INFRA TECH GEO SOULTIONS (M) SDN. BHD.	Drill Method: Rotary Wash	Supervisor: REDZA	Date Start : 6.07.2017
Maincon : INFRA TECH PROJECTS MALAYSIA SDN. BHD.	Casing Type : NW		Date Finish : 11.07.2017

Depth m	Strata Thickness	Description of Strata	Log	SAMPLING DETAIL Sample No	Depth m	Rec Ratio	Penetration, P (mm) 75	75	75	75	75	75	For <75	N Value	For <300
0	0.30	Top soil.		D	0.300										
1–2		Very soft<br>light grey<br>CLAY with decayed wood.		P1/D1	1.500<br>1.950	100%	0	0	0	0	0	0		0	
3		Very soft<br>light grey<br>CLAY with decayed wood.		P2/D2<br>VS 1	3.000<br>3.450<br>3.500	100%	0	0	0	0	0	0		0	
4–5		Very soft<br>light grey<br>CLAY with decayed wood.		P3/D3	4.500<br>4.950	100%	0	0	0	0	0	0		0	
5		Dark grey<br>CLAY of extremely high plasticity.		UD 1	5.000<br>5.500	100%									
6		Very soft<br>light grey<br>CLAY with decayed wood.		P4/D4<br>VS 2	6.000<br>6.450<br>6.500	100%	0	0	0	0	0	0		0	
7–8		Very soft<br>light grey<br>CLAY with lenses of fine grained sand and some organic material (seashell and decayed wood).		P5/D5	7.500<br>7.950	100%	0	0	0	0	0	0		0	
9		Very soft<br>light grey<br>CLAY with lenses of fine grained sand with seashell and decayed wood.		P6/D6<br>VS 3	9.000<br>9.450<br>9.500	89%	0	0	0	0	0	0		0	
10		Dark grey<br>CLAY of extremely high plasticity.		UD 2	10.000										
10–11		Very soft<br>light grey<br>CLAY with lenses of fine sand and some organic material (decayed wood and seashell).		P7/D7	10.500<br>10.950	100%	0	0	0	0	0	0		0	
12		Very soft<br>light grey<br>CLAY with some organic material (decayed wood).		P8/D8<br>VS 4	12.000<br>12.450<br>12.500	100%	0	0	0	0	0	0		0	
13–14		Very soft<br>light grey<br>CLAY with lenses of fine grained sand and some organic material (seashell).		P9/D9	13.500<br>13.950	100%	0	0	0	0	0	0		0	
15		Very soft<br>dark grey<br>CLAY with traces of fine grained sand and seashell.		P10/D10	15.000<br>15.450	100%	0	0	0	0	0	0		0	
15–16		Dark grey<br>CLAY of high plasticity.		UD 3	15.500<br>16.000										
16–17		Very soft<br>dark grey<br>sandy CLAY with some organic material which is seashell. Sand is fine grained.		P11/D11	16.500<br>16.950	100%	0	0	0	0	0	0		0	
18		Very soft<br>dark grey<br>sandy CLAY with some seashell and decayed wood. Sand is fine grained.		P12/D12	18.000<br>18.450	100%	0	0	0	0	0	0		0	
19–20		Very soft<br>dark grey<br>CLAY with lenses of fine grained sand and seashell and decayed wood.		P13/D13	19.500<br>19.950	100%	0	0	0	0	0	0		0	

Rec = Recovery

SPT PLOT

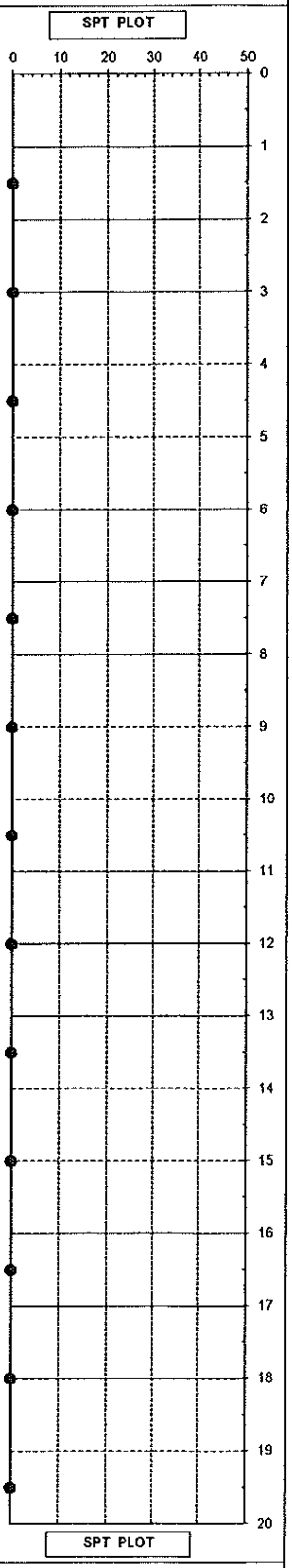

Legend:

- D – Disturbed Sample
- P – Standard Penetration Test
- UD – Undisturbed Sample
- MZ – Mazier Sample
- VS – Vane Shear Test
- C – Rock Coring
- W – Water Sample
- N - No. of Blows/300mm

NOTE:

** Existing ground level

Example:

50 | 120 = 50 Blows/120 mm

Cohesive Soil (N)	0	2	4	8	15	30	
	V.Soft,	Soft,	Firm,	Stiff,	V.Stiff,	Hard	
Non-cohesive Soil (N)	0	4	10	30	50		
	V.Loose	Loose	Med Dense	Dense	V.Dense		

HSD ENGINEERING SERVICES

ENGINEERING BOREHOLE LOG

Project : SOIL INVESTIGATION WORKS			Borehole No : BH 2
Location : BAGAN DATUK WATER CITY PHASE 1, PERAK DARUL RIDZUAN			Ground Level: 0.252 m
Client : PERBADANAN KEMAJUAN NEGERI PERAK	Rig Type : YWE D-90R	Driller : SHAH	Water Level : 0.42 m
Consultant: INFRA TECH GEO SOULTIONS (M) SDN. BHD.	Drill Method: Rotary Wash	Supervisor: REDZA	Date Start : 6.07.2017
Job No. : INFRA TECH PROJECTS MALAYSIA SDN. BHD.	Casing Type : NW		Date Finish : 11.07.2017

Depth m	Strata Thick-ness	Description of Strata	Log	SAMPLING DETAIL Sample No		Depth m	Rec Ratio	Penetration, P (mm) 75	75	75	75	75	75	For <75	N Value	For <300
								SPT BLOW COUNT								
20	21.00	Dark grey<br>CLAY of very high plasticity.		UD 4	UD	20.000<br>20.500	100%									
21		Very soft<br>dark grey<br>silty CLAY with decayed wood.	-- x	P14/D14	P	21.000<br>21.450	100%	0	0	0	0	0	0		0	
22		Very soft<br>light grey<br>silty CLAY with decayed wood.	-- x	P15/D15	P	22.500<br>22.950	100%	0	0	0	0	0	0		0	
23–24		Very soft<br>light grey<br>silty CLAY with decayed wood.	-- x	P16/D16	P	24.000<br>24.450	100%	0	0	0	0	0	0		0	
25		Very soft<br>dark grey<br>silty CLAY with decayed wood.	-- x	P17/D17	P	25.500<br>25.950	100%	0	0	0	0	0	0		0	
26–27		Very soft<br>dark grey<br>silty CLAY with decayed wood.	-- x	P18/D18	P	27.000<br>27.450	100%	0	0	0	0	0	0		0	
28		Very soft<br>dark grey<br>silty CLAY with decayed wood.	-- x	P19/D19	P	28.500<br>28.950	100%	0	0	0	0	0	0		0	
29–30		Very soft<br>dark grey<br>silty CLAY with decayed wood.	-- x	P20/D20	P	30.000<br>30.450	100%	0	0	0	0	0	0		0	
31		Very soft<br>dark grey<br>silty CLAY with lenses of fine grained sand and decayed wood.	-- x	P21/D21	P	31.500<br>31.950	100%	0	0	0	0	0	0		0	
32–33		Very soft<br>dark grey and light grey<br>silty CLAY with decayed wood.	-- x	P22/D22	P	33.000<br>33.450	100%	0	0	0	0	0	0		0	
34		Very soft<br>light grey<br>silty CLAY with fine to medium grained sand.	-- x	P23/D23	P	34.500<br>34.950	105%	0	0	0	0	0	0		0	
35–36		Very soft<br>light grey<br>sandy CLAY with decayed wood.	-- x	P24/D24	P	36.000<br>36.450	100%	0	0	0	0	0	0		0	
37		Very soft<br>dark grey<br>sandy CLAY with decayed wood. Sand is medium to coarse grained.	-- x	P25/D25	P	37.500<br>37.950	100%	0	0	0	0	0	0		0	
38–39		Soft<br>dark grey<br>silty CLAY with decayed wood.	-- x	P26/D26	P	39.000<br>39.450	100%	0	0	0	1	1	1		3	
40																

Rec = Recovery

SPT PLOT

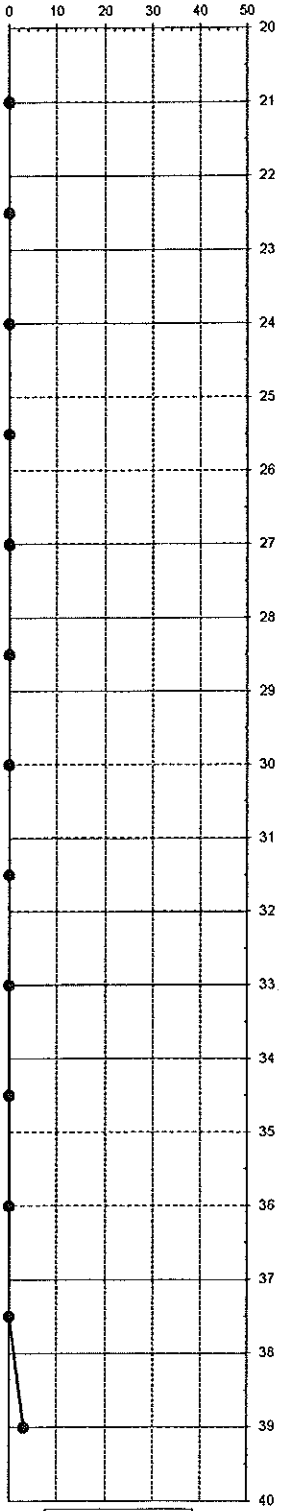

Legend:

- D — Disturbed Sample
- P — Standard Penetration Test
- UD — Undisturbed Sample
- MZ — Mazier Sample
- VS — Vane Shear Test
- C — Rock Coring
- W — Water Sample
- N - No. of Blows/300mm

NOTE:

** Existing ground level

Example:

50 | 120 = 50 Blows/120 mm

Cohesive Soil (N)	0	2	4	8	15	30
	V.Soft	Soft	Firm	Stiff	V.Stiff	Hard

Non-cohesive Soil (N)	0	4	10	30	50
	V.Loose	Loose	Med Dense	Dense	V.Dense

HSD ENGINEERING SERVICES

ENGINEERING BOREHOLE LOG

Project :	SOIL INVESTIGATION WORKS		Borehole No : **BH 2**
Location :	BAGAN DATUK WATER CITY PHASE 1, PERAK DARUL RIDZUAN		Ground Level: 0.252 m
Client :	PERBADANAN KEMAJUAN NEGERI PERAK	Rig Type : YWE D-90R / Driller : SHAH	Water Level : 0.42 m
Consultant:	INFRA TECH GEO SOULTIONS (M) SDN. BHD.	Drill Method: Rotary Wash / Supervisor: REDZA	Date Start : 6.07.2017
Job No. :	INFRA TECH PROJECTS MALAYSIA SDN. BHD.	Casing Type : NW	Date Finish : 11.07.2017

Depth m	Strata Thick-ness	Description of Strata	Log	Sample No	Depth m	Rec Ratio	75	75	75	75	75	75	For <75	N Value	For <300
40															
		Firm<br>dark grey<br>sandy CLAY with decayed wood.		P27/D27	40.500<br>40.950	83%	1	1	1	1	1	2		5	
41															
42		Very soft<br>light grey<br>sandy CLAY with decayed wood. Sand is fine grained.		P28/D28	42.000<br>42.450	100%	0	0	0	0	0	0		0	
43	43.50														
		Medium dense<br>light grey<br>medium to coarse SAND with some silt.		P29/D29	43.500<br>43.950	23%	1	2	4	4	5	7		20	
44															
45	45.45	Dense<br>grey<br>medium to coarse SAND.		P30/D30	45.000<br>45.450	49%	4	7	7	8	9	7		31	
46		End of BH 2 at 45.45 m depth.													
47															
48															
49															
50															
51															
52															
53															
54															
55															
56															
57															
58															
59															
60															

SAMPLING DETAIL; Penetration, P (mm); SPT BLOW COUNT; Rec = Recovery

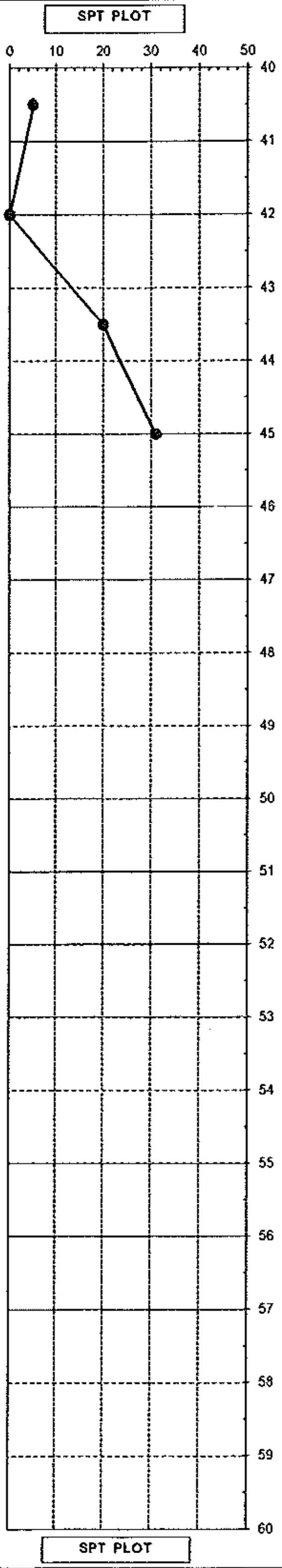

Legend:

- D — Disturbed Sample
- P — Standard Penetration Test
- UD — Undisturbed Sample
- MZ — Mazier Sample
- VS — Vane Shear Test
- C — Rock Coring
- W — Water Sample
- N - No. of Blows/300mm

NOTE:

** Existing ground level

Example:

50 | 120 = 50 Blows/120 mm

Cohesive Soil (N): 0 2 4 8 15 30 — V.Soft, Soft, Firm, Stiff, V.Stiff, Hard

Non-cohesive Soil (N): 0 4 10 30 50 — V.Loose Loose Med Dense Dense V.Dense

HSD ENGINEERING SERVICES

ENGINEERING BOREHOLE LOG

Project :	SOIL INVESTIGATION WORKS	Borehole No :	BH 3
Location :	BAGAN DATUK WATER CITY PHASE 1, PERAK DARUL RIDZUAN	Ground Level:	0.281 m
Client :	PERBADANAN KEMAJUAN NEGERI PERAK; Rig Type : YWE D-90R; Driller : SHAH	Water Level :	0.47 m
Consultant:	INFRA TECH GEO SOULTIONS (M) SDN. BHD.; Drill Method: Rotary Wash; Supervisor: REDZA	Date Start :	03.06.2017
Maincon :	INFRA TECH PROJECTS MALAYSIA SDN. BHD.; Casing Type : NW	Date Finish :	04.06.2017

Depth m	Strata Thick-ness	Description of Strata	Log	Sample No	Depth m	Rec Ratio	75	75	75	75	75	75	For <75	N Value	For <300
0	0.30			D	0.300										
1.5		Very sot grey CLAY.		P1/D1	1.500 1.950	100%	0	0	0	0	0	0		0	
3		Very sot grey CLAY.		P2/D2	3.000 3.450	100%	0	0	0	0	0	0		0	
4.5		Very sot grey CLAY.		P3/D3	4.500 4.950	100%	0	0	0	0	0	0		0	
6		Very soft grey CLAY.		P4/D4	6.000 6.450	100%	0	0	0	0	0	0		0	
7.5		Very soft grey CLAY with some seashell.		P5/D5	7.500 7.950	100%	0	0	0	0	0	0		0	
8		Dark grey CLAY of high plasticity.		UD 1	8.000 8.500	100%									
9		Very soft grey CLAY with lenses of fine grained sand and some seashell.		P6/D6	9.000 9.450	100%	0	0	0	0	0	0		0	
10.5		Very soft grey CLAY with lenses of fine grained sand and some seashell.		P7/D7	10.500 10.950	100%	0	0	0	0	0	0		0	
11		Dark grey CLAY of extremely high plasticity.		UD 2	11.000 11.500	100%									
12		Very soft grey CLAY with lenses of fine grained sand and some seashell.		P8/D8	12.000 12.450	100%	0	0	0	0	0	0		0	
13.5	15.00	Very soft grey CLAY with lenses of fine grained sand and some seashell.		P9/D9	13.500 13.950	100%	0	0	0	0	0	0		0	
15	16.50	Very loose grey silty fine SAND with decayed wood.		P10/D10	15.000 15.450	100%	0	0	0	1	0	0		1	
16.5	17.50	Very soft grey CLAY with lenses of fine grained sand and decayed wood.		P11/D11	16.500 16.950	100%	0	0	0	0	1	0		1	
17	18.00	Dark grey sandy CLAY of intermediate plasticity.		UD 3	17.000 17.500	60%									
18		Medium dense grey silty fine to medium coarse SAND with some seashell.		P12/D12	18.000 18.450	11%	0	2	4	4	3	4		15	
19.5		Very soft grey CLAY with decayed wood.		P13/D13	19.500 19.950	100%	0	0	0	0	0	0		0	
20						Rec = Recovery									

SPT PLOT

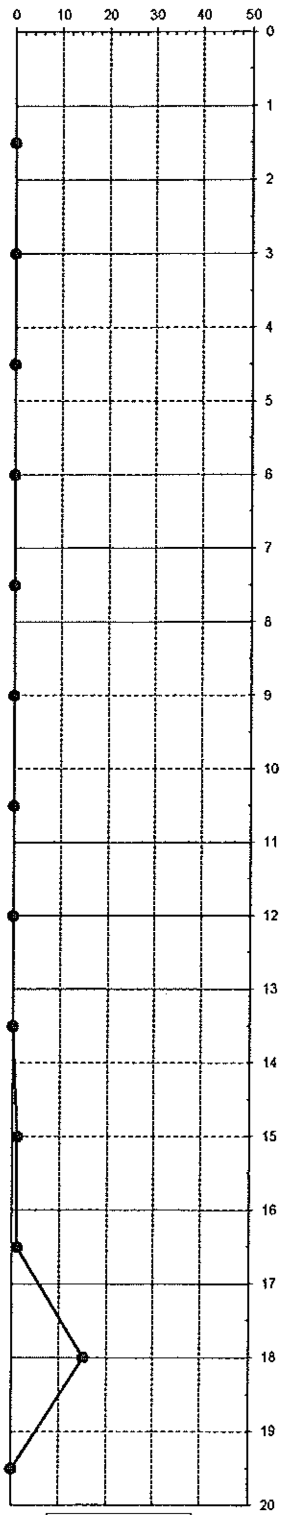

Legend:

- D Disturbed Sample
- P Standard Penetration Test
- UD Undisturbed Sample
- MZ Mazier Sample
- VS Vane Shear Test
- C Rock Coring
- W Water Sample
- N - No. of Blows/300mm

NOTE:

** Existing ground level

Example:

50 | 120 = 50 Blows/120 mm

Cohesive Soil (N)	0	2	4	8	15	30
	V.Soft,	Soft,	Firm,	Stiff,	V.Stiff,	Hard

Non-cohesive Soil (N)	0	4	10	30	50
	V.Loose	Loose	Med Dense	Dense	V.Dense

HSD ENGINEERING SERVICES

ENGINEERING BOREHOLE LOG

Project :	SOIL INVESTIGATION WORKS	Borehole No :	BH 3
Location :	BAGAN DATUK WATER CITY PHASE 1, PERAK DARUL RIDZUAN	Ground Level:	0.281 m

Client : PERBADANAN KEMAJUAN NEGERI PERAK	Rig Type : YWE D-90R	Driller : SHAH	Water Level : 0.47 m
Consultant: INFRA TECH GEO SOULTIONS (M) SDN. BHD.	Drill Method: Rotary Wash	Supervisor: REDZA	Date Start : 03.06.2017
Job No. : INFRA TECH PROJECTS MALAYSIA SDN. BHD.	Casing Type : NW		Date Finish : 04.06.2017

Depth m	Strata Thickness	Description of Strata	Log	Sample No	Depth m	Rec Ratio	75	75	75	75	75	75	For <75	N Value	For <300
20	21.00	Dark grey CLAY of very high plasticity.		UD 4	20.000 20.500	100%									
21		Very soft grey silty CLAY.		P14/D14	21.000 21.450	100%	0	0	0	0	0	0		0	
22		Very soft grey silty CLAY.		P15/D15	22.500 22.950	100%	0	0	0	0	0	0		0	
24		Very soft grey silty CLAY with decayed wood.		P16/D16	24.000 24.450	100%	0	0	0	0	0	0		0	
25		Very soft grey silty CLAY with decayed wood.		P17/D17	25.500 25.950	100%	0	0	0	0	0	0		0	
27		Very soft grey silty CLAY with decayed wood.		P18/D18	27.000 27.450	100%	0	0	0	0	0	0		0	
28		Soft to firm grey silty CLAY with decayed wood.		P19/D19	28.500 28.950	100%	0	0	1	1	1	1		4	
30		Soft grey silty CLAY with decayed wood.		P20/D20	30.000 30.450	100%	0	0	1	0	1	1		3	
31	33.00	Very stiff light grey silty CLAY interbedded with fine to medium coarse sand some organic material.		P21/D21	31.500 31.950	100%	1	2	4	4	6	10		24	
33	34.50	Dark brown decayed wood.		P22/D22	33.000 33.450	80%	1	2	4	4	6	9		23	
34		Soft grey silty CLAY.		P23/D23	34.500 34.950	100%	1	3	1	0	1	1		3	
36		Very soft grey silty CLAY.		P24/D24	36.000 36.450	100%	0	0	0	0	0	0		0	
37		Very soft grey silty CLAY interbedded with fine to medium coarse sand and fragment of angular cobbles.		P25/D25	37.500 37.950	100%	0	1	0	1	0	1		2	
39		Stiff dark grey silty CLAY with decayed wood.		P26/D26	39.000 39.450	100%	0	1	2	3	2	2		9	
40															

Rec = Recovery

SPT PLOT

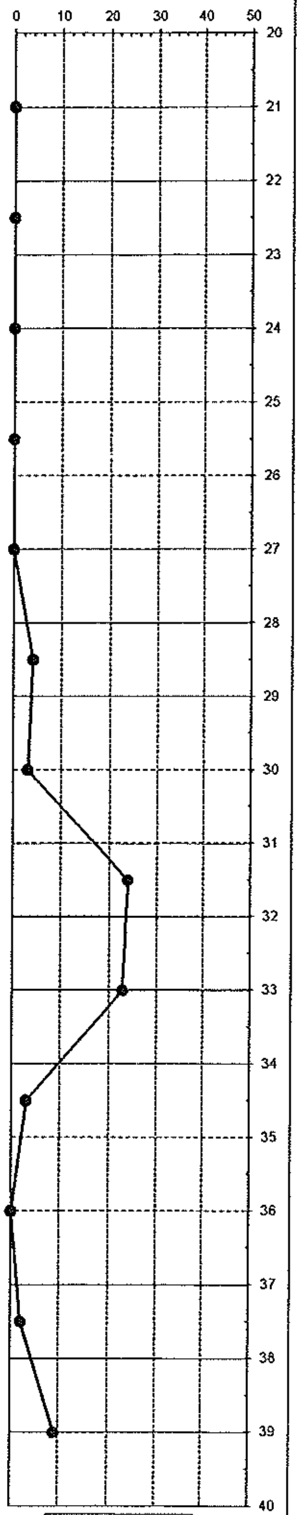

SPT PLOT

Legend:

- D Disturbed Sample
- P Standard Penetration Test
- UD Undisturbed Sample
- MZ Mazier Sample
- VS Vane Shear Test
- C Rock Coring
- W Water Sample
- N - No. of Blows/300mm

NOTE:

** Existing ground level

Example:

50 | 120 = 50 Blows/120 mm

Cohesive Soil (N)	0	2	4	8	15	30	
	V.Soft,	Soft,	Firm,	Stiff,	V.Stiff,	Hard	
Non-cohesive Soil (N)	0	4	10	30	50		
	V.Loose	Loose	Med Dense	Dense	V.Dense		

HSD ENGINEERING SERVICES

ENGINEERING BOREHOLE LOG

Project :	SOIL INVESTIGATION WORKS	Borehole No :	BH 3
Location :	BAGAN DATUK WATER CITY PHASE 1, PERAK DARUL RIDZUAN	Ground Level:	0.281 m
Client :	PERBADANAN KEMAJUAN NEGERI PERAK<br>Rig Type : YWE D-90R<br>Driller : SHAH	Water Level :	0.47 m
Consultant:	INFRA TECH GEO SOULTIONS (M) SDN. BHD.<br>Drill Method: Rotary Wash<br>Supervisor: REDZA	Date Start :	03.06.2017
Job No. :	INFRA TECH PROJECTS MALAYSIA SDN. BHD.<br>Casing Type : NW	Date Finish :	04.06.2017

Depth m	Strata Thick-ness	Description of Strata	Log	Sample No	Depth m	Rec Ratio	75	75	75	75	75	75	For <75	N Value	For <300
40															
41		Stiff light grey silty CLAY.		P27/D27	40.500<br>40.950	100%	1	2	3	3	3	4		13	
42		Very stiff light grey silty CLAY interbedded with medium to coarse sand.		P28/D28	42.000<br>42.450	82%	2	6	6	7	7	7		27	
43	43.50														
44		Medium dense light grey fine to medium coarse SAND with some granular and decayed wood.		P29/D29	43.500<br>43.950	2%	1	1	3	4	4	5		16	
45	45.45	Medium dense light grey medium to coarse grained SAND with some gravels.		P30/D30	45.000<br>45.450	1%	0	2	6	7	5	5		23	
46		End of BH 3 at 45.45 m depth.													
47															
48															
49															
50															
51															
52															
53															
54															
55															
56															
57															
58															
59															
60															

SAMPLING DETAIL; Penetration, P (mm); SPT BLOW COUNT; Rec = Recovery

SPT PLOT

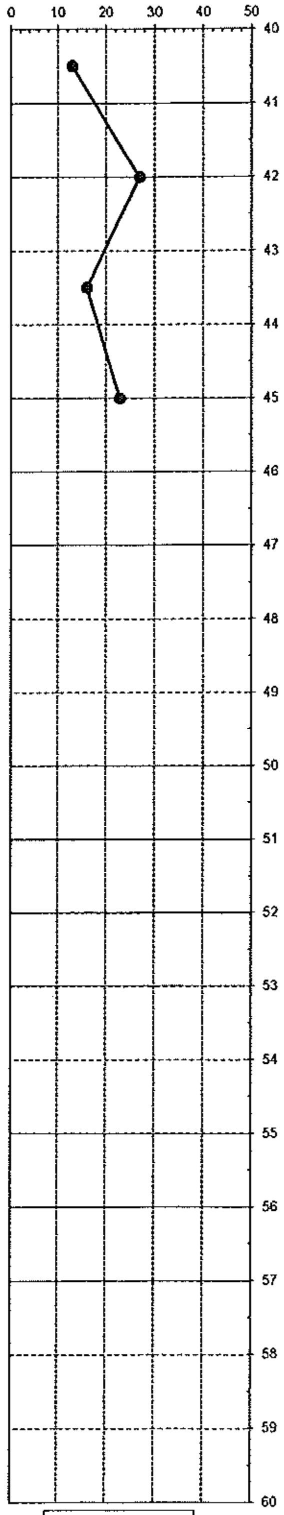

SPT PLOT

Legend:

- D — Disturbed Sample
- P — Standard Penetration Test
- UD — Undisturbed Sample
- MZ — Mazier Sample
- VS — Vane Shear Test
- C — Rock Coring
- W — Water Sample
- N - No. of Blows/300mm

NOTE:

** Existing ground level

Example:

50 | 120 = 50 Blows/120 mm

Cohesive Soil (N)	0	2	4	8	15	30	
	V.Soft,	Soft,	Firm,	Stiff,	V.Stiff,	Hard	
Non-cohesive Soil (N)	0	4	10	30	50		
	V.Loose	Loose	Med Dense	Dense	V.Dense		

HSD ENGINEERING SERVICES

ENGINEERING BOREHOLE LOG

Project :	SOIL INVESTIGATION WORKS	Borehole No :	BH 4
Location :	BAGAN DATUK WATER CITY PHASE 1, PERAK DARUL RIDZUAN	Ground Level:	0.160 m

Client :	PERBADANAN KEMAJUAN NEGERI PERAK	Rig Type :	YWE D-90R	Driller :	SHAH	Water Level :	0.60 m
Consultant:	INFRA TECH GEO SOULTIONS (M) SDN. BHD.	Drill Method:	Rotary Wash	Supervisor:	REDZA	Date Start :	07.05.2017
Maincon :	INFRA TECH PROJECTS MALAYSIA SDN. BHD.	Casing Type :	NW			Date Finish :	11.05.2017

Depth m	Strata Thickness	Description of Strata	Log	Sample No	Type	Depth m	Rec Ratio	75	75	75	75	75	75	For <75	N Value	For <300
0	0.30	Top soil			D	0.300										
1.5		Very soft dark grey CLAY.		P1/D1	P	1.500<br>1.950	100%	1	0	0	0	0	0		1	
3		Very soft dark grey CLAY.		P2/D2	P	3.000<br>3.450	100%	0	0	0	0	0	0		0	
3.5		Dark grey CLAY.		UD 1	UD	3.500<br>4.000	100%									
4.5		Very soft dark grey CLAY.		P3/D3	P	4.500<br>4.950	100%	0	0	0	0	0	0		0	
6		Very soft dark grey CLAY.		P4/D4	P	6.000<br>6.450	100%	0	0	0	0	0	0		0	
6.5		Dark grey CLAY of high plasticity.		UD 2	UD	6.500<br>7.000	100%									
7.5		Very soft dark grey CLAY.		P5/D5	P	7.500<br>7.950	100%	0	0	0	0	0	0		0	
9		Very soft medium to dark grey sandy CLAY.		P6/D6	P	9.000<br>9.450	100%	0	0	0	0	0	0		0	
9.5		Dark grey CLAY.		UD 3	UD	9.000<br>9.500	100%									
10.5		Very soft medium to dark grey CLAY.		P7/D7	P	10.500<br>10.950	100%	0	0	0	0	0	0		0	
12		Very soft medium grey CLAY.		P8/D8	P	12.000<br>12.450	100%	0	0	0	0	0	0		0	
12.5		Dark grey CLAY of high plasticity.		UD 4	UD	12.500<br>13.000	100%									
13.5		Very soft medium grey CLAY.		P9/D9	P	13.500<br>13.950	100%	0	0	0	0	0	0		0	
15		Very soft to soft medium grey CLAY.		P10/D10	P	15.000<br>15.450	100%	1	0	1	0	1	0		2	
15.5		Dark grey CLAY.		UD 5	UD	15.500<br>16.000	100%									
16.5		Soft dark grey CLAY.		P11/D11	P	16.500<br>16.950	100%	1	0	1	0	1	1		3	
18		Soft dark grey CLAY.		P12/D12	P	18.000<br>18.450	100%	1	0	1	0	1	1		3	
18.5		Dark grey CLAY of extremely high plasticity.		UD 6	UD	18.500<br>19.000	100%									
19.5		Very soft medium to dark grey CLAY.		P13/D13	P	19.500<br>19.950	100%	1	0	0	1	0	0		1	
20																

Rec = Recovery

SPT PLOT

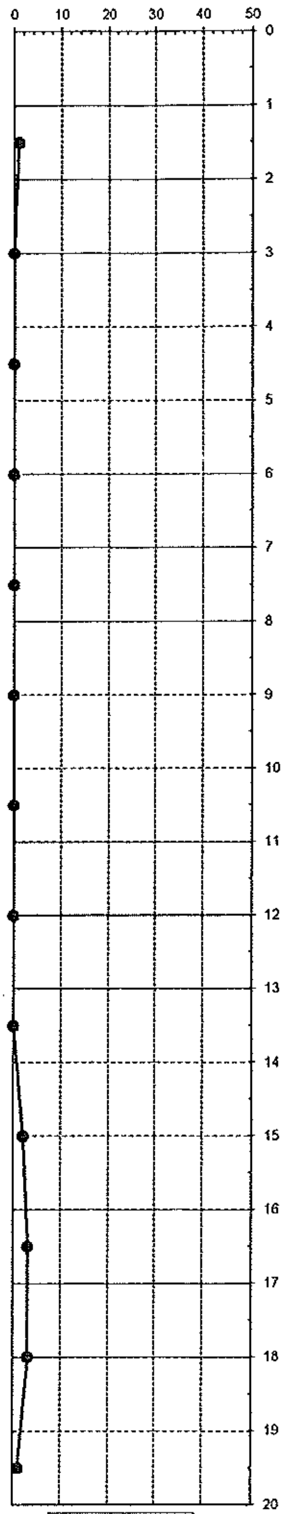

Legend:

- D — Disturbed Sample
- P — Standard Penetration Test
- UD — Undisturbed Sample
- MZ — Mazier Sample
- VS — Vane Shear Test
- C — Rock Coring
- W — Water Sample
- N · No. of Blows/300mm

NOTE:

** Existing ground level

Example:

50 | 120 = 50 Blows/120 mm

Cohesive Soil (N)	0	2	4	8	15	30	
	V.Soft,	Soft,	Firm,	Stiff,	V.Stiff,	Hard	
Non-cohesive Soil (N)	0	4	10	30	50		
	V.Loose	Loose	Med Dense	Dense	V.Dense		

HSD ENGINEERING SERVICES

ENGINEERING BOREHOLE LOG

Project :	SOIL INVESTIGATION WORKS	Borehole No :	BH 4
Location :	BAGAN DATUK WATER CITY PHASE 1, PERAK DARUL RIDZUAN	Ground Level:	0.160 m
Client :	PERBADANAN KEMAJUAN NEGERI PERAK	Rig Type : YWE D-90R	Driller : SHAH
Consultant:	INFRA TECH GEO SOULTIONS (M) SDN. BHD.	Drill Method: Rotary Wash	Supervisor: REDZA
Maincon :	INFRA TECH PROJECTS MALAYSIA SDN. BHD.	Casing Type : NW	
Water Level :	0.60 m	Date Start :	07.05.2017
Date Finish :	11.05.2017		

Depth m	Strata Thick-ness	Description of Strata	Log	Sampling Detail: Sample No	Depth m	Rec Ratio	Penetration, P (mm) 75	75	75	75	75	75	For <75	N Value	For <300
20															
21		Very soft medium grey CLAY.		P14/D14	21.000 21.450	100%	0	0	0	0	0	0		0	
		Dark grey CLAY.		UD 7	21.500 22.000	100%									
22															
		Very soft medium grey CLAY.		P15/D15	22.500 22.950	100%	0	0	0	0	0	0		0	
23															
24		Very soft dark grey CLAY.		P16/D16	24.000 24.450	100%	0	0	0	0	0	0		0	
25															
		Very soft dark grey CLAY.		P17/D17	25.500 25.950	100%	0	0	0	0	0	0		0	
26															
27		Very soft dark grey CLAY.		P18/D18	27.000 27.450	100%	0	0	0	0	0	0		0	
28															
		Very soft dark grey CLAY.		P19/D19	28.500 28.950	100%	0	0	0	0	0	0		0	
29															
30		Very soft medium grey mottled with dark grey CLAY.		P20/D20	30.000 30.450	100%	0	0	0	0	0	0		0	
31															
		Very soft medium grey mottled with dark grey CLAY.		P21/D21	31.500 31.950	100%	0	0	0	0	0	0		0	
32															
33		Very soft medium grey CLAY.		P22/D22	33.000 33.450	100%	0	0	0	0	0	0		0	
34															
		Very soft medium grey CLAY.		P23/D23	34.500 34.950	100%	0	0	0	0	0	0		0	
35	36.00														
36		Very soft medium grey sandy CLAY.		P24/D24	36.000 36.450	100%	0	0	0	0	0	0		0	
37															
		Very soft medium grey sandy CLAY.		P25/D25	37.500 37.950	100%	0	0	0	0	0	0		0	
38															
39		Very soft medium grey sandy CLAY.		P26/D26	39.000 39.450	100%	0	0	0	0	0	0		0	
40															

Rec = Recovery

SPT PLOT

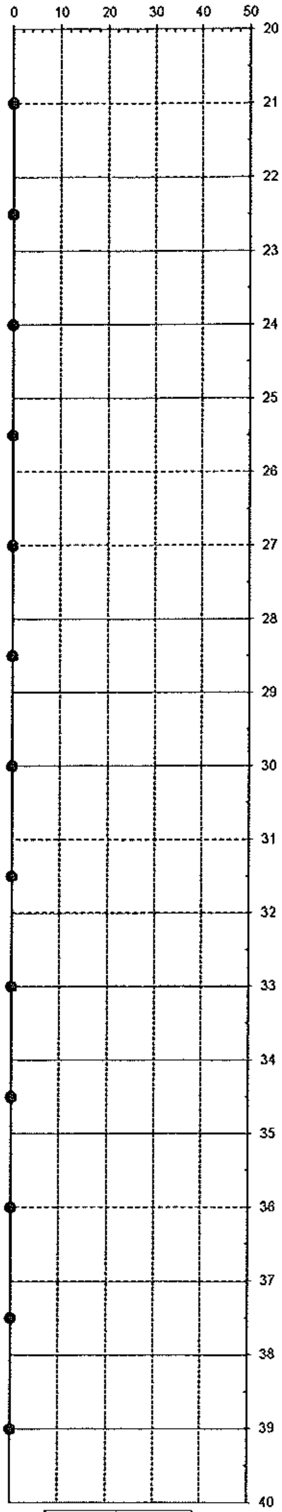

SPT PLOT

Legend:

- D — Disturbed Sample
- P — Standard Penetration Test
- UD — Undisturbed Sample
- MZ — Mazier Sample
- VS — Vane Shear Test
- C — Rock Coring
- W — Water Sample
- N - No. of Blows/300mm

NOTE:

** Existing ground level

Example:

50 | 120 = 50 Blows/120 mm

Cohesive Soil (N)	0	2	4	8	15	30	
	V.Soft,	Soft,	Firm,	Stiff,	V.Stiff,	Hard	
Non-cohesive Soil (N)	0	4	10	30	50		
	V.Loose	Loose	Med Dense	Dense	V.Dense		

HSD ENGINEERING SERVICES

ENGINEERING BOREHOLE LOG

Project :	SOIL INVESTIGATION WORKS	Borehole No :	**BH 4**
Location :	BAGAN DATUK WATER CITY PHASE 1, PERAK DARUL RIDZUAN	Ground Level:	0.160 m
Client :	PERBADANAN KEMAJUAN NEGERI PERAK	Water Level :	0.60 m
Consultant:	INFRA TECH GEO SOULTIONS (M) SDN. BHD.	Date Start :	07.05.2017
Maincon :	INFRA TECH PROJECTS MALAYSIA SDN. BHD.	Date Finish :	11.05.2017

Rig Type :	YWE D-90R	Driller :	SHAH
Drill Method:	Rotary Wash	Supervisor:	REDZA
Casing Type :	NW		

Depth m	Strata Thickness	Description of Strata	Log	Sample No	Depth m	Rec Ratio	75	75	75	75	75	75	For <75	N Value	For <300
40															
		Very soft medium grey sandy CLAY.		P27/D27	40.500<br>40.950	100%	0	0	0	0	0	0		0	
41															
42		Very soft dark grey sandy CLAY.		P28/D28	42.000<br>42.450	100%	0	0	0	0	0	0		0	
43		Very soft dark grey sandy CLAY.		P29/D29	43.500<br>43.950	100%	0	0	0	0	0	0		0	
44															
45	45.45	Very soft medium grey sandy CLAY.		P30/D30	45.000<br>45.450	100%	0	0	0	0	0	0		0	
46		End of BH 4 at 45.45 m depth.													
47															
48															
49															
50															
51															
52															
53															
54															
55															
56															
57															
58															
59															
60															

Rec = Recovery

SPT PLOT

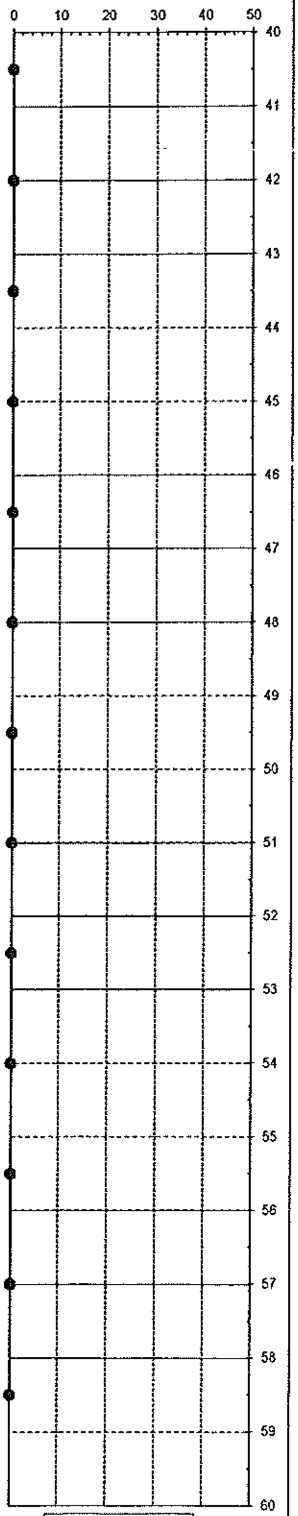

SPT PLOT

Legend:

- D – Disturbed Sample
- P – Standard Penetration Test
- UD – Undisturbed Sample
- MZ – Mazier Sample
- VS – Vane Shear Test
- C – Rock Coring
- W – Water Sample
- N - No. of Blows/300mm

NOTE:

** Existing ground level

Example:

50 | 120 = 50 Blows/120 mm

Cohesive Soil (N)	0	2	4	8	15	30
	V.Soft,	Soft,	Firm,	Stiff,	V.Stiff,	Hard
Non cohesive Soil (N)	0	4	10	30	50	
	V.Loose	Loose	Med Dense	Dense	V.Dense	

HSD ENGINEERING SERVICES

ENGINEERING BOREHOLE LOG

Project :	SOIL INVESTIGATION WORKS	Borehole No :	BH 5
Location :	BAGAN DATUK WATER CITY PHASE 1, PERAK DARUL RIDZUAN	Ground Level:	0.150 m
Client :	PERBADANAN KEMAJUAN NEGERI PERAK	Rig Type : YWE D-90R	Driller : SHAH
Consultant:	INFRA TECH GEO SOULTIONS (M) SDN. BHD.	Drill Method: Rotary Wash	Supervisor: REDZA
Maincon :	INFRA TECH PROJECTS MALAYSIA SDN. BHD.	Casing Type : NW	
Water Level :	0.32 m	Date Start :	11.05.2017
Date Finish :	18.05.2017		

Depth m	Strata Thickness	Description of Strata	Sample No	Depth m	Rec Ratio	75	75	75	75	75	75	For <75	N Value	For <300
0														
		Top soil												
	0.30		D	0.300										
1														
		Very soft dark brown CLAY.	P1/D1	1.500 1.950	100%	0	0	0	0	0	0		0	
2														
3		Very soft dark brown CLAY.	P2/D2	3.000 3.450	100%	0	0	0	0	0	0		0	
		Dark brown CLAY of very high plasticity.	UD 1	3.500 4.000	100%									
4														
		Very soft dark brown CLAY.	P3/D3	4.500 4.950	100%	0	0	0	0	0	0		0	
5														
6		Very soft medium grey sandy CLAY.	P4/D4	6.000 6.450	100%	0	0	0	0	0	0		0	
7														
		Very soft medium grey CLAY.	P5/D5	7.500 7.950	100%	0	0	0	0	0	0		0	
8														
		Medium grey CLAY of extremely high plasticity.	UD 2	8.000 8.500	100%									
9		Very soft medium grey CLAY.	P6/D6	9.000 9.450	100%	0	0	0	0	0	0		0	
10														
		Very soft medium grey CLAY.	P7/D7	10.500 10.950	100%	0	0	0	0	0	0		0	
11														
12		Very soft dark grey CLAY.	P8/D8	12.000 12.450	100%	0	0	0	0	0	0		0	
		Dark grey CLAY of extremely high plasticity.	UD 3	12.500 13.000	100%									
13														
		Very soft dark grey CLAY.	P9/D9	13.500 13.950	100%	0	0	0	0	0	0		0	
14														
15		Very soft medium grey CLAY.	P10/D10	15.000 15.450	100%	0	0	0	0	0	0		0	
16														
		Very soft medium grey CLAY.	P11/D11	16.500 16.950	100%	0	0	0	0	0	0		0	
17														
		Medium grey CLAY of very high plasticity.	UD 4	17.000 17.500	100%									
	18.00													
18		Very soft medium grey sandy CLAY.	P12/D12	18.000 18.450	100%	0	0	0	0	0	0		0	
19														
		Very soft medium grey sandy CLAY.	P13/D13	19.500 19.950	100%	0	0	0	0	0	0		0	
20														

Rec = Recovery

SPT PLOT

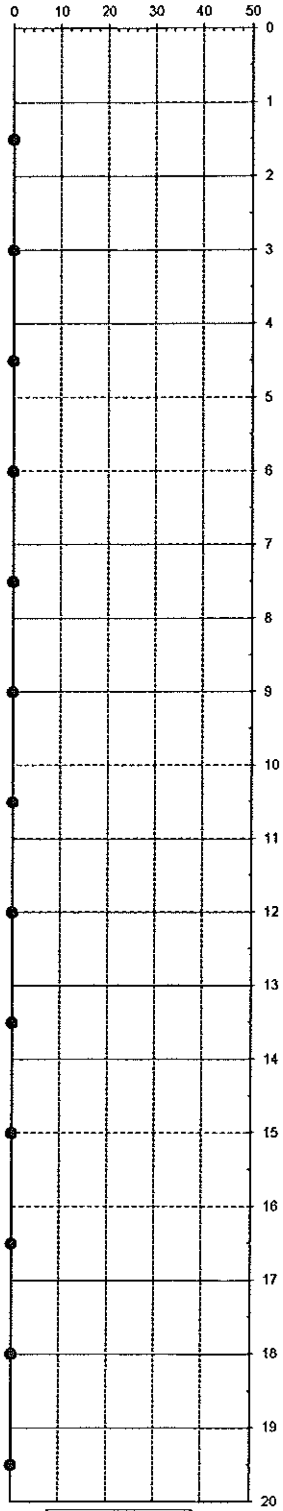

SPT PLOT

Legend:

- D — Disturbed Sample
- P — Standard Penetration Test
- UD — Undisturbed Sample
- MZ — Mazier Sample
- VS — Vane Shear Test
- C — Rock Coring
- W — Water Sample
- N - No. of Blows/300mm

NOTE:

** Existing ground level

Example:

50 | 120 = 50 Blows/120 mm

Cohesive Soil (N)	0	2	4	8	15	30
	V.Soft,	Soft,	Firm,	Stiff,	V.Stiff,	Hard

Non-cohesive Soil (N)	0	4	10	30	50
	V.Loose	Loose	Med Dense	Dense	V.Dense

HSD ENGINEERING SERVICES

ENGINEERING BOREHOLE LOG

Project : SOIL INVESTIGATION WORKS			Borehole No : BH 5
Location : BAGAN DATUK WATER CITY PHASE 1, PERAK DARUL RIDZUAN			Ground Level: 0.150 m
Client : PERBADANAN KEMAJUAN NEGERI PERAK	Rig Type : YWE D-90R	Driller : SHAH	Water Level : 0.32 m
Consultant: INFRA TECH GEO SOULTIONS (M) SDN. BHD.	Drill Method: Rotary Wash	Supervisor: REDZA	Date Start : 11.05.2017
Maincon : INFRA TECH PROJECTS MALAYSIA SDN. BHD.	Casing Type : NW		Date Finish : 18.05.2017

Depth m	Strata Thickness	Description of Strata	Log	Sample No	Depth m	Rec Ratio	75	75	75	75	75	75	For <75	N Value	For <300
21	22.50	Very soft dark grey sandy CLAY.		P14/D14	21.000 21.450	100%	0	0	0	0	0	0		0	
22.5		Very soft dark grey CLAY.		P15/D15	22.500 22.950	100%	0	0	0	0	0	0		0	
24		Very soft dark grey CLAY.		P16/D16	24.000 24.450	100%	1	0	0	1	0	0		1	
25.5	27.00	Very soft to soft dark grey CLAY.		P17/D17	25.500 25.950	100%	1	0	0	1	0	1		2	
27		Very soft dark grey sandy CLAY.		P18/D18	27.000 27.450	100%	0	0	0	0	0	0		0	
28.5		Very soft dark grey sandy CLAY.		P19/D19	28.500 28.950	100%	0	0	0	0	0	0		0	
30		Very soft dark grey sandy CLAY.		P20/D20	30.000 30.450	100%	0	0	0	0	0	0		0	
31.5		Very soft dark grey sandy CLAY.		P21/D21	31.500 31.950	100%	0	0	0	0	0	0		0	
33		Stiff light grey sandy CLAY.		P22/D22	33.000 33.450	100%	1	2	2	2	3	3		10	
34.5		Stiff light grey sandy CLAY.		P23/D23	34.500 34.950	100%	2	2	3	3	3	3		12	
36		Very stiff medium grey sandy CLAY.		P24/D24	36.000 36.450	100%	5	6	4	3	5	5		17	
37.5		Very stiff dark grey sandy CLAY.		P25/D25	37.500 37.950	100%	4	5	5	7	6	7		25	
39		Firm medium grey sandy CLAY.		P26/D26	39.000 39.450	100%	1	2	1	1	2	1		5	

Rec = Recovery

SPT PLOT

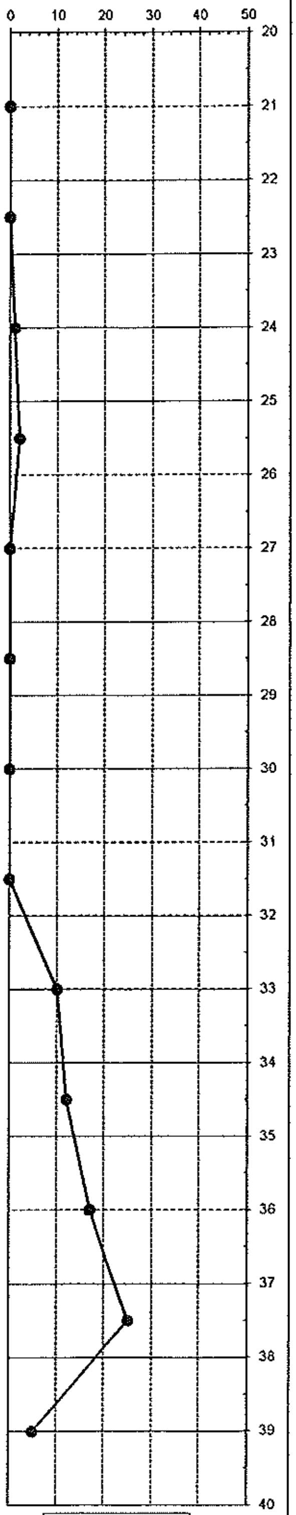

SPT PLOT

Legend:

- D – Disturbed Sample
- P – Standard Penetration Test
- UD – Undisturbed Sample
- MZ – Mazier Sample
- VS – Vane Shear Test
- C – Rock Coring
- W – Water Sample
- N - No. of Blows/300mm

NOTE:

** Existing ground level

Example:

50 | 120 = 50 Blows/120 mm

Cohesive Soil (N): 0, 2, 4, 8, 15, 30 — V.Soft, Soft, Firm, Stiff, V.Stiff, Hard

Non-cohesive Soil (N): 0, 4, 10, 30, 50 — V.Loose, Loose, Med Dense, Dense, V.Dense

HSD ENGINEERING SERVICES

ENGINEERING BOREHOLE LOG

Project :	SOIL INVESTIGATION WORKS	Borehole No :	BH 5
Location :	BAGAN DATUK WATER CITY PHASE 1, PERAK DARUL RIDZUAN	Ground Level:	0.150 m
Client :	PERBADANAN KEMAJUAN NEGERI PERAK	Rig Type : YWE D-90R	Driller : SHAH
Consultant:	INFRA TECH GEO SOULTIONS (M) SDN. BHD.	Drill Method: Rotary Wash	Supervisor: REDZA
Maincon :	INFRA TECH PROJECTS MALAYSIA SDN. BHD.	Casing Type : NW	
Water Level :	0.32 m	Date Start :	11.05.2017
Date Finish :	18.05.2017		

Depth m	Strata Thickness	Description of Strata	Log	Sample No	Depth m	Rec Ratio	75	75	75	75	75	75	For <75	N Value	For <300
				SAMPLING DETAIL			Penetration, P (mm) / SPT BLOW COUNT								
40 – 45.00	45.00	Firm medium grey sandy CLAY.		P27/D27	40.500<br>40.950	100%	1	2	2	1	1	2		6	
		Stiff medium grey sandy CLAY.		P28/D28	42.000<br>42.450	100%	1	2	2	3	2	3		10	
		Stiff medium grey sandy CLAY.		P29/D29	43.500<br>43.950	100%	1	3	2	3	3	3		11	
45.00 – 45.45	45.45	Stiff to very stiff medium grey CLAY with some fine sand.		P30/D30	45.000<br>45.450	100%	2	2	4	3	4	4		15	
		End of BH 5 at 45.45 m depth.													

Rec = Recovery

SPT PLOT

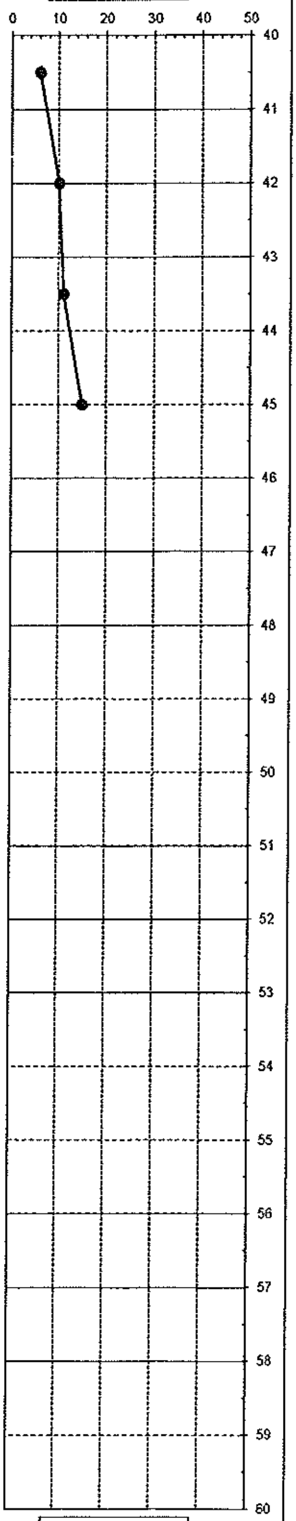

SPT PLOT

Legend:

- D Disturbed Sample
- P Standard Penetration Test
- UD Undisturbed Sample
- MZ Mazier Sample
- VS Vane Shear Test
- C Rock Coring
- W Water Sample
- N - No. of Blows/300mm

NOTE:

** Existing ground level

Example:

50 | 120 = 50 Blows/120 mm

Cohesive Soil (N)	0	2	4	8	15	30	
	V.Soft	Soft	Firm	Stiff	V.Stiff	Hard	
Non-cohesive Soil (N)	0	4	10	30	50		
	V.Loose	Loose	Med Dense	Dense	V.Dense		

HSD ENGINEERING SERVICES

ENGINEERING BOREHOLE LOG

Project :	SOIL INVESTIGATION WORKS	Borehole No :	BH 6
Location :	BAGAN DATUK WATER CITY PHASE 1, PERAK DARUL RIDZUAN	Ground Level:	0.227 m
Client :	PERBADANAN KEMAJUAN NEGERI PERAK	Rig Type : YWE D-90R	Driller : SHAH	Water Level : 1.00 m
Consultant:	INFRA TECH GEO SOULTIONS (M) SDN. BHD.	Drill Method: Rotary Wash	Supervisor: REDZA	Date Start : 04.06.2017
Maincon :	INFRA TECH PROJECTS MALAYSIA SDN. BHD.	Casing Type : NW		Date Finish : 06.06.2017

Depth m	Strata Thickness	Description of Strata	Log	Sample No	Depth m	Rec Ratio	75	75	75	75	75	75	For <75	N Value	For <300
				SAMPLING DETAIL			Penetration, P (mm) / SPT BLOW COUNT							N	
0	0.30	Top soil		D	0.300										
1, 2		Very soft dark grey CLAY.		P1/D1	1.500 1.950	100%	0	0	0	0	0	0		0	
3, 4	4.50	Very soft dark grey CLAY.		P2/D2	3.000 3.450	100%	0	0	0	0	0	0		0	
5	6.00	Dark grey sandy CLAY of intermediate plasticity.		UD 1	4.500 5.000	100%									
6, 7		Very soft dark grey CLAY.		P3/D3	6.000 6.450	100%	0	0	0	0	0	0		0	
8		Very soft dark grey CLAY.		P4/D4	7.500 7.950	100%	0	0	0	0	0	0		0	
9, 10		Very soft dark grey CLAY.		P5/D5	9.000 9.450	100%	0	0	0	0	0	0		0	
11	12.00	Dark grey CLAY of extremely high plasticity.		UD 2	10.500 11.000	100%									
12, 13		Very soft medium grey sandy CLAY.		P6/D6	12.000 12.450	100%	0	0	0	0	0	0		0	
14	15.00	Medium grey sandy CLAY of intermediate plasticity.		UD 3	13.500 14.000	100%								0	
15, 16		Very soft dark grey CLAY.		P7/D7	15.000 15.450	100%	0	0	0	0	0	0		0	
17	18.00	Very soft dark grey CLAY.		P8/D8	16.500 16.950	100%	0	0	0	0	0	0		0	
18, 19	19.50	No recovery.		P9	18.000 18.450	0%	0	0	0	1	1	0		2	
20		Dark grey sandy CLAY of intermediate plasticity.		UD 4	19.500 20.000	100%									

Rec = Recovery

SPT PLOT

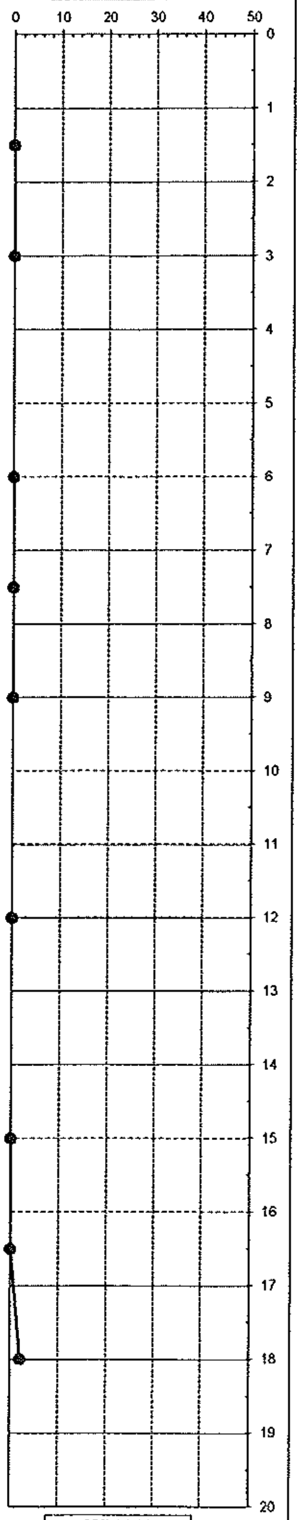

SPT PLOT

Legend:

- D — Disturbed Sample
- P — Standard Penetration Test
- UD — Undisturbed Sample
- MZ — Mazier Sample
- VS — Vane Shear Test
- C — Rock Coring
- W — Water Sample
- N - No. of Blows/300mm

NOTE:

** Existing ground level

Example:

50 | 120 = 50 Blows/120 mm

Cohesive Soil (N): 0, 2, 4, 8, 15, 30 — V.Soft, Soft, Firm, Stiff, V.Stiff, Hard

Non-cohesive Soil (N): 0, 4, 10, 30, 50 — V.Loose, Loose, Med Dense, Dense, V.Dense

HSD ENGINEERING SERVICES

ENGINEERING BOREHOLE LOG

Project :	SOIL INVESTIGATION WORKS	Borehole No :	BH 6
Location :	BAGAN DATUK WATER CITY PHASE 1, PERAK DARUL RIDZUAN	Ground Level:	0.227 m
Client :	PERBADANAN KEMAJUAN NEGERI PERAK	Rig Type : YWE D-90R	Driller : SHAH
Consultant:	INFRA TECH GEO SOULTIONS (M) SDN. BHD.	Drill Method: Rotary Wash	Supervisor: REDZA
Maincon :	INFRA TECH PROJECTS MALAYSIA SDN. BHD.	Casing Type : NW	
Water Level :	1.00 m	Date Start :	04.06.2017
Date Finish :	06.06.2017		

Depth m	Strata Thick-ness	Description of Strata	Log	Sample No	Depth m	Rec Ratio	75	75	75	75	75	75	For <75	N Value	For <300
20															
21		Firm dark grey CLAY.		P10/D9	21.000 21.450	96%	1	0	1	1	1	2		5	
22		Firm dark grey CLAY.		P11/D10	22.500 22.950	100%	1	1	1	1	2	2		6	
23															
24		Very soft to soft dark grey CLAY.		P12/D11	24.000 24.450	100%	1	0	0	1	0	1		2	
25		Very soft to soft dark grey CLAY.		P13/D12	25.500 25.950	100%	1	0	0	1	0	1		2	
26															
27		Soft dark grey CLAY..		P14/D13	27.000 27.450	100%	1	0	1	0	1	1		3	
28		Firm dark grey CLAY.		P15/D14	28.500 28.950	100%	1	1	1	1	1	2		5	
29															
30		Firm to stiff light grey CLAY.		P16/D15	30.000 30.450	100%	2	2	2	2	2	2		8	
31		Soft to firm dark grey CLAY.		P17/D16	31.500 31.950	100%	1	1	2	0	1	1		4	
32															
33	33.00	Hard medium grey sandy CLAY.		P18/D17	33.000 33.450	89%	3	6	6	9	11	11		37	
34		Very stiff dark grey sandy CLAY.		P19/D18	34.500 34.950	100%	3	3	4	6	7	7		24	
35															
36	36.00	Dense medium grey clayey SAND.		P20/D19	36.000 36.450	71%	3	7	8	11	8	8		35	
37		Dense medium grey clayey SAND.		P21/D20	37.500 37.950	56%	2	4	7	7	9	6		29	
38															
39		Loose to medium dense medium grey clayey SAND.		P22/D21	39.000 39.450	100%	2	2	2	2	3	3		10	
40															

SAMPLING DETAIL — Penetration, P (mm) — SPT BLOW COUNT — Rec = Recovery

SPT PLOT

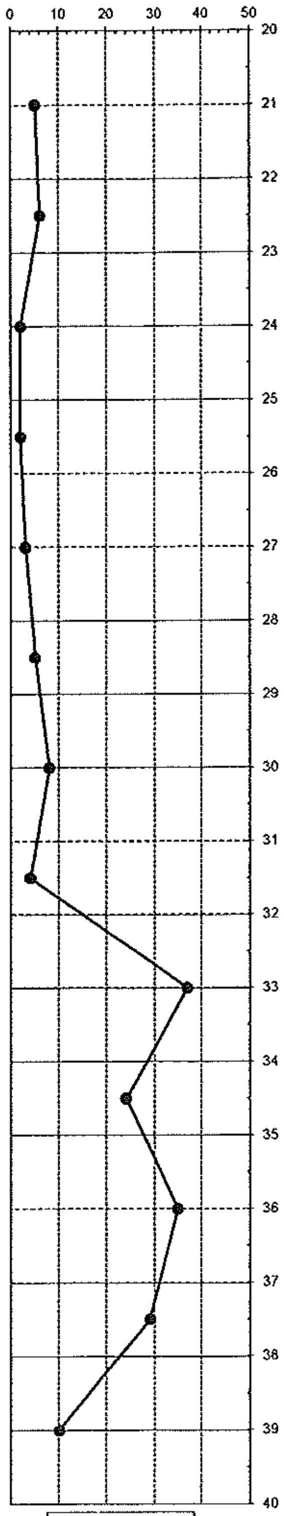

SPT PLOT

Legend:

- D — Disturbed Sample
- P — Standard Penetration Test
- UD — Undisturbed Sample
- MZ — Mazier Sample
- VS — Vane Shear Test
- C — Rock Coring
- W — Water Sample
- N - No. of Blows/300mm

NOTE:

** Existing ground level

Example:

50 | 120 = 50 Blows/120 mm

Cohesive Soil (N): 0, 2, 4, 8, 15, 30 — V.Soft, Soft, Firm, Stiff, V.Stiff, Hard

Non-cohesive Soil (N): 0, 4, 10, 30, 50 — V.Loose, Loose, Med Dense, Dense, V.Dense

HSD ENGINEERING SERVICES

ENGINEERING BOREHOLE LOG

Project : SOIL INVESTIGATION WORKS			Borehole No : BH 6
Location : BAGAN DATUK WATER CITY PHASE 1, PERAK DARUL RIDZUAN			Ground Level: 0.227 m
Client : PERBADANAN KEMAJUAN NEGERI PERAK	Rig Type : YWE D-90R	Driller : SHAH	Water Level : 1.00 m
Consultant: INFRA TECH GEO SOULTIONS (M) SDN. BHD.	Drill Method: Rotary Wash	Supervisor: REDZA	Date Start : 04.06.2017
Maincon : INFRA TECH PROJECTS MALAYSIA SDN. BHD.	Casing Type : NW		Date Finish : 06.06.2017

Depth m	Strata Thick-ness	Description of Strata	Log	Sample No		Depth m	Rec Ratio	75	75	75	75	75	75	For <75	N Value	For <300
				SAMPLING DETAIL				Penetration, P (mm) / SPT BLOW COUNT								
40																
41		Stiff dark grey CLAY.	---	P23/D22	P	40.500 40.950	100%	1	1	2	3	2	2		9	
42		Stiff medium grey CLAY.	---	P24/D23	P	42.000 42.450	98%	2	2	2	2	3	3		10	
43		Stiff medium grey CLAY.	---	P25/D24	P	43.500 43.950	89%	1	2	3	3	2	3		11	
44																
45	45.45	Stiff light grey CLAY.	---	P26/D25	P	45.000 45.450	71%	2	3	3	3	4	4		14	
46		End of BH 6 at 45.50m depth.														
47																
48																
49																
50																
51																
52																
53																
54																
55																
56																
57																
58																
59																
60																

Rec = Recovery

SPT PLOT

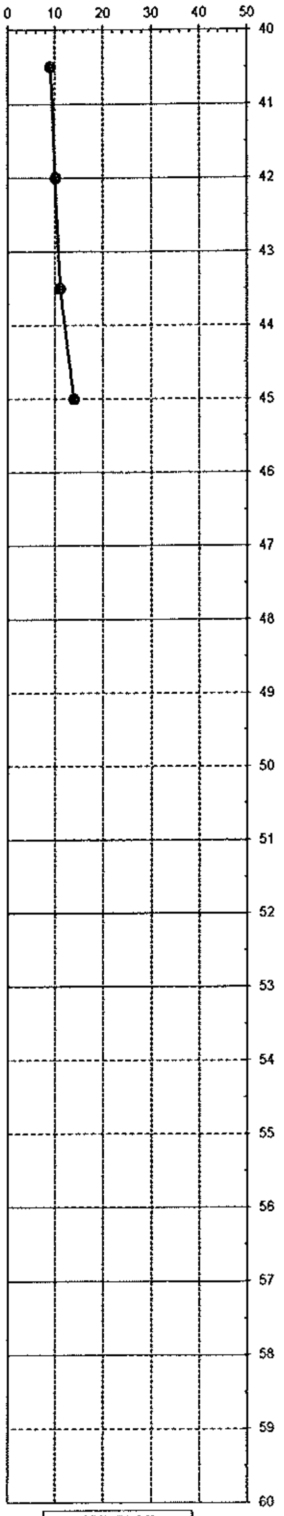

SPT PLOT

Legend:

- D – Disturbed Sample
- P – Standard Penetration Test
- UD – Undisturbed Sample
- MZ – Mazier Sample
- VS – Vane Shear Test
- C – Rock Coring
- W – Water Sample
- N - No. of Blows/300mm

NOTE:

** Existing ground level

HSD ENGINEERING SERVICES

Example:

50 | 120 = 50 Blows/120 mm

Cohesive Soil (N): 0, 2, 4, 8, 15, 30 — V.Soft, Soft, Firm, Stiff, V.Stiff, Hard

Non-cohesive Soil (N): 0, 4, 10, 30, 50 — V.Loose, Loose, Med Dense, Dense, V.Dense

ENGINEERING BOREHOLE LOG

Project :	SOIL INVESTIGATION WORKS
Location :	BAGAN DATUK WATER CITY PHASE 1, PERAK DARUL RIDZUAN
Client :	PERBADANAN KEMAJUAN NEGERI PERAK
Consultant:	INFRA TECH GEO SOULTIONS (M) SDN. BHD.
Maincon :	INFRA TECH PROJECTS MALAYSIA SDN. BHD.
Rig Type :	YWE D-90R
Drill Method:	Rotary Wash
Casing Type :	NW
Driller :	SHAH
Supervisor:	REDZA
Borehole No :	BH 7
Ground Level:	0.261 m
Water Level :	0.80 m
Date Start :	28.05.2017
Date Finish :	01.06.2017

Depth m	Strata Thick-ness	Description of Strata	Log	SAMPLING DETAIL Sample No	Depth m	Rec Ratio	Penetration, P (mm) 75	75	75	75	75	75	For <75	N Value	For <300
							SPT BLOW COUNT								
0	0.30	Top soil.		D	0.300										
1.5	3.00	Very soft dark grey CLAY.		P1/D1	1.500 / 1.950	100%	0	0	0	0	0	0		0	
3	4.50	Dark grey sandy CLAY of intermediate plasticity.		UD 1	3.000 / 3.500	100%									
4.5		Very soft medium grey CLAY.		P2/D2	4.500 / 4.950	100%	0	0	0	0	0	0		0	
6		Very soft medium grey CLAY.		P3/D3	6.000 / 6.450	100%	0	0	0	0	0	0		0	
7.5	9.00	Medium grey CLAY of high plasticity.		UD 2	7.500 / 8.000	100%									
9	10.50	Very soft medium grey sandy CLAY.		P4/D4	9.000 / 9.450	100%	0	0	0	0	0	0		0	
10.5		Very soft medium grey CLAY.		P5/D5	10.500 / 10.950	100%	0	0	0	0	0	0		0	
12		Medium grey CLAY of very high plasticity.		UD 3	12.000 / 12.500	100%									
13.5		Very soft medium grey CLAY.		P6/D6	13.500 / 13.950	100%	0	0	0	0	0	0		0	
15		Very soft medium grey CLAY.		P7/D7	15.000 / 15.450	100%	0	0	0	0	0	0		0	
16.5		Medium grey CLAY of high plasticity.		UD 4	16.500 / 17.000	100%								0	
18		Very soft dark grey CLAY.		P8/D8	18.000 / 18.450	100%	0	0	0	0	0	0		0	
19.5		Very soft dark grey CLAY.		P9/D9	19.500 / 19.950	100%	0	0	0	0	0	0		0	
20						Rec = Recovery									

SPT PLOT

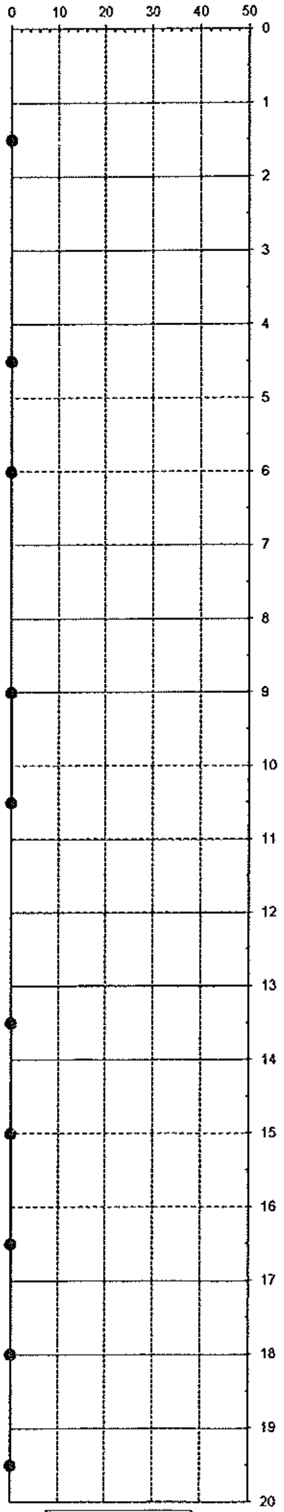

Legend:

- D — Disturbed Sample
- P — Standard Penetration Test
- UD — Undisturbed Sample
- MZ — Mazier Sample
- VS — Vane Shear Test
- C — Rock Coring
- W — Water Sample
- N - No. of Blows/300mm

NOTE:

** Existing ground level

Example:

50 | 120 = 50 Blows/120 mm

Cohesive Soil (N)	0	2	4	8	15	30
	V.Soft	Soft	Firm	Stiff	V.Stiff	Hard
Non-cohesive Soil (N)	0	4	10	30	50	
	V.Loose	Loose	Med Dense	Dense	V.Dense	

HSD ENGINEERING SERVICES

ENGINEERING BOREHOLE LOG

Project :	SOIL INVESTIGATION WORKS	Borehole No :	BH 7
Location :	BAGAN DATUK WATER CITY PHASE 1, PERAK DARUL RIDZUAN	Ground Level:	0.261 m
Client :	PERBADANAN KEMAJUAN NEGERI PERAK; Rig Type : YWE D-90R; Driller : SHAH	Water Level :	0.80 m
Consultant:	INFRA TECH GEO SOULTIONS (M) SDN. BHD.; Drill Method: Rotary Wash; Supervisor: REDZA	Date Start :	28.05.2017
Maincon :	INFRA TECH PROJECTS MALAYSIA SDN. BHD.; Casing Type : NW	Date Finish :	01.06.2017

Depth m	Strata Thickness	Description of Strata	Log	Sample No	Depth m	Rec Ratio	75	75	75	75	75	75	For <75	N Value	For <300
20															
21		Very soft dark grey CLAY.		P10/D10	21.000<br>21.450	100%	0	0	0	0	0	0		0	
22		Very soft dark grey CLAY.		P11/D11	22.500<br>22.950	100%	0	0	0	0	0	0		0	
23															
24		Soft to very soft dark grey CLAY.		P12/D12	24.000<br>24.450	100%	0	0	0	0	0	0		0	
25		Very soft medium grey CLAY.		P13/D13	25.500<br>25.950	100%	0	0	0	0	0	0		0	
26															
27		Very soft medium grey CLAY.		P14/D14	27.000<br>27.450	100%	0	0	0	0	0	0		0	
28		Very soft dark grey CLAY.		P15/D15	28.500<br>28.950	100%	0	0	0	0	0	0		0	
29															
30		Very soft dark grey CLAY with decayed wood.		P16/D16	30.000<br>30.450	100%	0	0	0	0	0	0		0	
31		Very soft dark grey CLAY.		P17/D17	31.500<br>31.950	100%	1	0	0	0	1	0		1	
32	33.00														
33		Hard light grey sandy CLAY.		P18/D18	33.000<br>33.450	98%	2	3	7	9	17	17		50	
34	34.50	Firm light grey CLAY.		P19/D19	34.500<br>34.950	100%	2	1	1	2	1	1		5	
35															
36		Hard medium grey CLAY with traces of coarse sand.		P20/D20	36.000<br>36.450	100%	11	14	20	10	8	5		43	
37	37.50	Very stiff to hard light grey sandy CLAY.		P21/D21	37.500<br>37.950	100%	2	4	4	4	3	4		15	
38	39.00														
39		Stiff light grey mottled with white CLAY.		P22/D22	39.000<br>39.450	100%	1	2	2	2	3	4		11	
40															

Penetration, P (mm) — SPT BLOW COUNT. Rec = Recovery

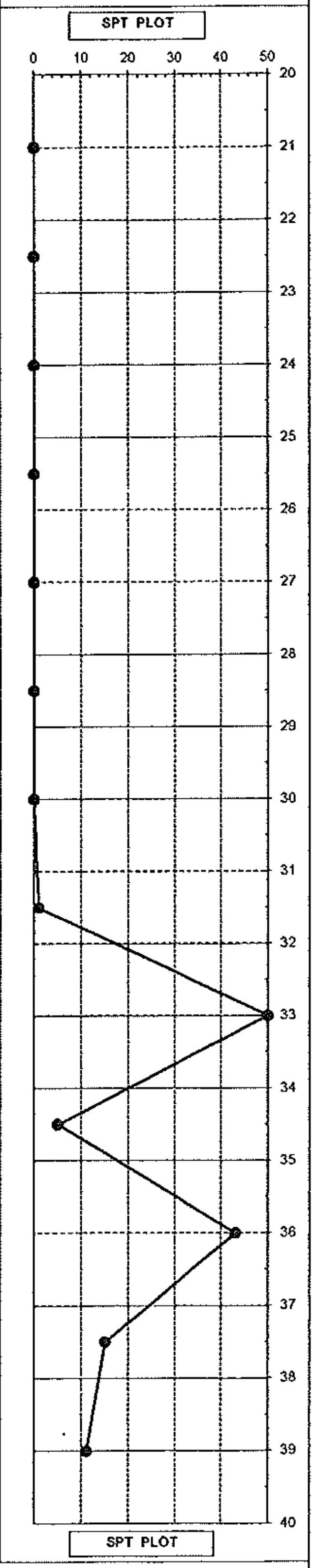

Legend:

- D — Disturbed Sample
- P — Standard Penetration Test
- UD — Undisturbed Sample
- MZ — Mazier Sample
- VS — Vane Shear Test
- C — Rock Coring
- W — Water Sample
- N - No. of Blows/300mm

NOTE:

** Existing ground level

Example:

50 | 120 = 50 Blows/120 mm

Cohesive Soil (N)	0	2	4	8	15	30	
	V.Soft,	Soft,	Firm,	Stiff,	V.Stiff,	Hard	
Non-cohesive Soil (N)	0	4	10	30	50		
	V.Loose	Loose	Med Dense	Dense	V.Dense		

HSD ENGINEERING SERVICES

ENGINEERING BOREHOLE LOG

Project :	SOIL INVESTIGATION WORKS
Location :	BAGAN DATUK WATER CITY PHASE 1, PERAK DARUL RIDZUAN
Client :	PERBADANAN KEMAJUAN NEGERI PERAK
Consultant:	INFRA TECH GEO SOULTIONS (M) SDN. BHD.
Maincon :	INFRA TECH PROJECTS MALAYSIA SDN. BHD.
Rig Type :	YWE D-90R
Drill Method:	Rotary Wash
Casing Type :	NW
Driller :	SHAH
Supervisor:	REDZA
Borehole No :	BH 7
Ground Level:	0.261 m
Water Level :	0.80 m
Date Start :	28.05.2017
Date Finish :	01.06.2017

Depth m	Strata Thickness	Description of Strata	Sample No	Depth m	Rec Ratio	75	75	75	75	75	75	For <75	N Value	For <300
40.500–40.950		Stiff light grey mottled with brown CLAY.	P23/D23	40.500 / 40.950	100%	1	2	3	3	4	4		14	
42.000–42.450		Stiff light grey mottled with dark grey CLAY.	P24/D24	42.000 / 42.450	100%	2	3	3	3	3	4		13	
43.500–43.950		Very stiff dark grey CLAY.	P25/D25	43.500 / 43.950	100%	2	2	4	4	3	5		16	
45.000–45.450	45.45	Very stiff light grey mottled with dark grey CLAY.	P26/D26	45.000 / 45.450	100%	3	3	3	4	5	5		17	
		End of BH 7 at 45.45 m depth.												

Depth scale: 40 – 60 m. SAMPLING DETAIL; Penetration, P (mm); SPT BLOW COUNT. Rec = Recovery

SPT PLOT

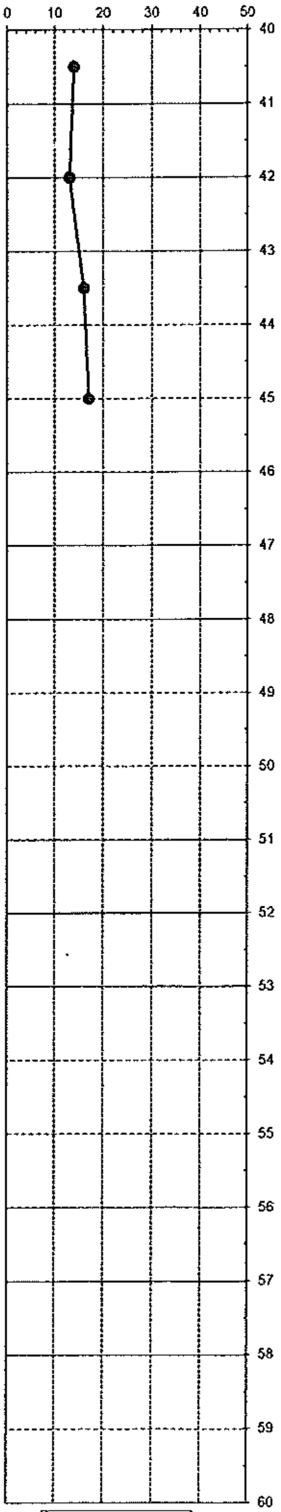

Legend:

- D – Disturbed Sample
- P – Standard Penetration Test
- UD – Undisturbed Sample
- MZ – Mazier Sample
- VS – Vane Snear Test
- C – Rock Coring
- W – Water Sample
- N - No. of Blows/300mm

NOTE:

** Existing ground level

Example:

50 | 120 = 50 Blows/120 mm

Cohesive Soil (N): 0, 2, 4, 8, 15, 30 — V.Soft, Soft, Firm, Stiff, V.Stiff, Hard

Non-cohesive Soil (N): 0, 4, 10, 30, 50 — V.Loose, Loose, Med Dense, Dense, V.Dense

HSD ENGINEERING SERVICES

ENGINEERING BOREHOLE LOG

Project : SOIL INVESTIGATION WORKS			Borehole No : BH 8
Location : BAGAN DATUK WATER CITY PHASE 1, PERAK DARUL RIDZUAN			Ground Level: 0.012 m
Client : PERBADANAN KEMAJUAN NEGERI PERAK	Rig Type : YWE D-90R	Driller : SHAH	Water Level : 0.39 m
Consultant: INFRA TECH GEO SOULTIONS (M) SDN. BHD.	Drill Method: Rotary Wash	Supervisor: REDZA	Date Start : 18.05.2017
Maincon : INFRA TECH PROJECTS MALAYSIA SDN. BHD.	Casing Type : NW		Date Finish : 24.05.2017

Depth m	Strata Thick-ness	Description of Strata	Log	Sample No	Depth m	Rec Ratio	75	75	75	75	75	75	For <75	N Value	For <300
0	0.30	Top soil		D	0.300										
1.5		Very soft dark grey CLAY.		P1/D1	1.500 / 1.950	100%	0	0	0	0	0	0		0	
3.0		Very soft dark grey CLAY.		P2/D2	3.000 / 3.450	100%	0	0	0	0	0	0		0	
4.5		Very soft dark grey CLAY.		P3/D3	4.500 / 4.950	100%	0	0	0	0	0	0		0	
5.0		dark grey CLAY of extremely high plasticity.		UD 1	5.000 / 5.500	100%									
6.0		Very soft dark grey CLAY.		P4/D4	6.000 / 6.450	100%	0	0	0	0	0	0		0	
7.5		Very soft medium grey CLAY.		P5/D5	7.500 / 7.950	100%	0	0	0	0	0	0		0	
9.0		Very soft medium grey CLAY.		P6/D6	9.000 / 9.450	100%	0	0	0	0	0	0		0	
9.5		Medium grey CLAY of extremely high plasticity.		UD 2	9.500 / 10.000	100%									
10.5		Very soft medium grey CLAY.		P7/D7	10.500 / 10.950	100%	0	0	0	0	0	0		0	
12.0		Very soft dark grey CLAY.		P8/D8	12.000 / 12.450	100%	0	0	0	0	0	0		0	
13.5		Very soft dark grey CLAY.		P9/D9	13.500 / 13.950	100%	0	0	0	0	0	0		0	
14.0		Dark grey CLAY of extremely high plasticity.		UD 3	14.000 / 14.500	100%									
15.0		Very soft dark grey CLAY.		P10/D10	15.000 / 15.450	100%	0	0	0	0	0	0		0	
16.5		Very soft dark grey CLAY.		P11/D11	16.500 / 16.950	100%	0	0	0	0	0	0		0	
18.0		Very soft dark grey CLAY.		P12/D12	18.000 / 18.450	100%	0	0	0	0	0	0		0	
18.5	19.50	Dark grey CLAY of very high plasticity.		UD 4	18.500 / 19.000	100%									
19.5		Very soft dark grey sandy CLAY.		P13/D13	19.500 / 19.950	100%	0	0	0	0	0	0		0	

SAMPLING DETAIL — Penetration, P (mm) — SPT BLOW COUNT — Rec = Recovery

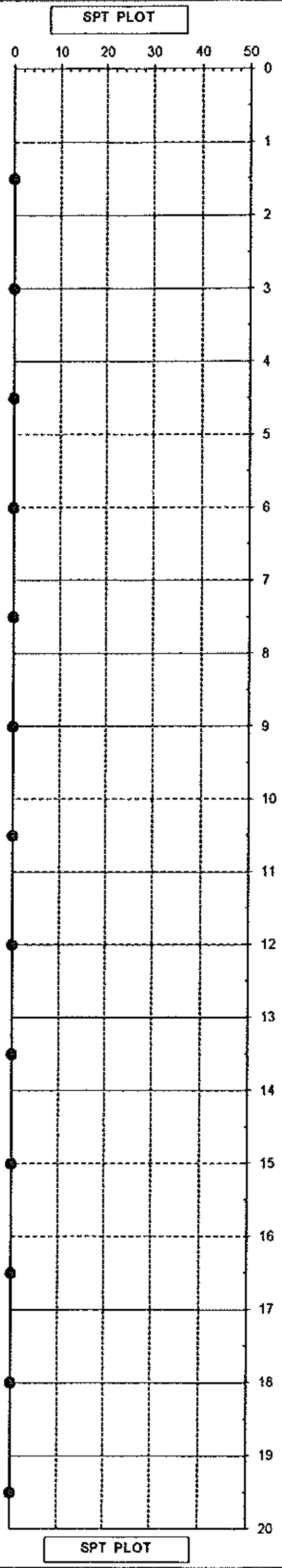

Legend:

- D — Disturbed Sample
- P — Standard Penetration Test
- UD — Undisturbed Sample
- MZ — Mazier Sample
- VS — Vane Shear Test
- C — Rock Coring
- W — Water Sample
- N - No. of Blows/300mm

NOTE:

** Existing ground level

Example:

50 | 120 = 50 Blows/120 mm

Cohesive Soil (N)	0	2	4	8	15	30	
	V.Soft	Soft	Firm	Stiff	V.Stiff	Hard	
Non-cohesive Soil (N)	0	4	10	30	50		
	V.Loose	Loose	Med Dense	Dense	V.Dense		

HSD ENGINEERING SERVICES

ENGINEERING BOREHOLE LOG

Project :	SOIL INVESTIGATION WORKS	Borehole No :	BH 8
Location :	BAGAN DATUK WATER CITY PHASE 1, PERAK DARUL RIDZUAN	Ground Level:	0.012 m

Client :	PERBADANAN KEMAJUAN NEGERI PERAK	Rig Type :	YWE D-90R	Driller :	SHAH	Water Level :	0.39 m
Consultant:	INFRA TECH GEO SOULTIONS (M) SDN. BHD.	Drill Method:	Rotary Wash	Supervisor:	REDZA	Date Start :	18.05.2017
Maincon :	INFRA TECH PROJECTS MALAYSIA SDN. BHD.	Casing Type :	NW			Date Finish :	24.05.2017

Depth m	Strata Thickness	Description of Strata	Log	Sample No	Depth m	Rec Ratio	75	75	75	75	75	75	For <75	N Value	For <300
20															
21		Very soft dark grey sandy CLAY.		P14/D14	21.000 21.450	100%	0	0	0	0	0	0		0	
22		Very soft dark grey sandy CLAY with traces of gravels.		P15/D15	22.500 22.950	100%	0	0	0	0	0	0		0	
24	24.00	Very soft dark grey CLAY.		P16/D16	24.000 24.450	100%	1	0	0	1	0	1		2	
25		Soft dark grey CLAY.		P17/D17	25.500 25.950	100%	1	0	1	1	0	1		3	
27		Very soft dark grey CLAY.		P18/D18	27.000 27.450	100%	0	0	0	0	0	0		0	
28		Very soft dark grey CLAY.		P19/D19	28.500 28.950	100%	0	0	0	0	0	0		0	
30		Very soft dark grey CLAY.		P20/D20	30.000 30.450	100%	0	0	0	0	0	0		0	
31		Very soft dark grey CLAY.		P21/D21	31.500 31.950	100%	0	0	0	0	0	0		0	
33		Hard medium grey sandy CLAY.		P22/D22	33.000 33.450	89%	2	6	7	13	18	12	75	50	300
34		Hard medium grey sandy CLAY.		P23/D23	34.500 34.850	78%	4	7	9	19	22		50	50	200
36	36.00	No recovery.		P24	36.000 36.000	0%	HAMMER REBOUND							50	
37	37.50	Stiff light grey streaked with black CLAY.		P25/D24	37.500 37.950	100%	2	3	2	3	3	4		12	
39	39.00	Stiff medium grey streaked with black sandy CLAY.		P26/D25	39.000 39.450	100%	1	2	3	4	4	4		15	
40															

Rec = Recovery

SPT PLOT

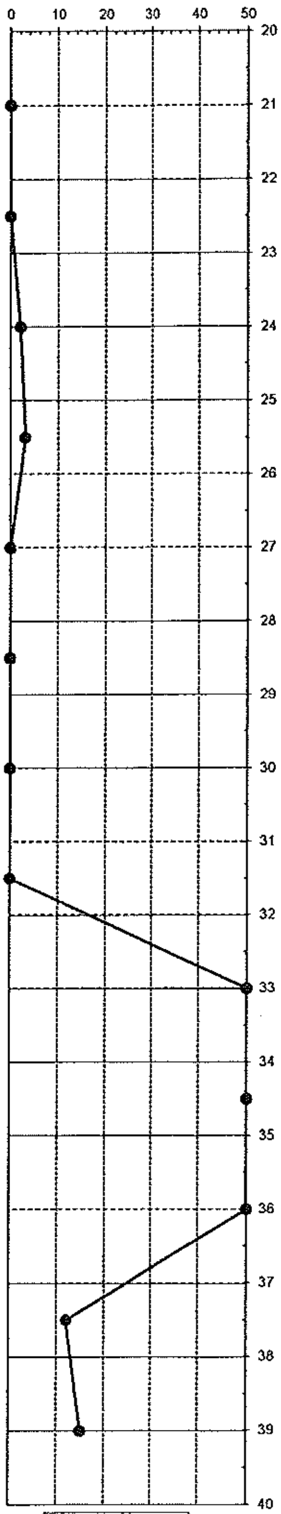

SPT PLOT

Legend:

- D — Disturbed Sample
- P — Standard Penetration Test
- UD — Undisturbed Sample
- MZ — Mazier Sample
- VS — Vane Shear Test
- C — Rock Coring
- W — Water Sample
- N - No. of Blows/300mm

NOTE:

** Existing ground level

Example:

50 | 120 = 50 Blows/120 mm

Cohesive Soil (N): 0 V.Soft, 2 Soft, 4 Firm, 8 Stiff, 15 V.Stiff, 30 Hard

Non-cohesive Soil (N): 0 V.Loose, 4 Loose, 10 Med Dense, 30 Dense, 50 V.Dense

HSD ENGINEERING SERVICES

ENGINEERING BOREHOLE LOG

Project :	SOIL INVESTIGATION WORKS		Borehole No : BH 8
Location :	BAGAN DATUK WATER CITY PHASE 1, PERAK DARUL RIDZUAN		Ground Level: 0.012 m
Client :	PERBADANAN KEMAJUAN NEGERI PERAK	Rig Type : YWE D-90R / Driller : SHAH	Water Level : 0.39 m
Consultant:	INFRA TECH GEO SOULTIONS (M) SDN. BHD.	Drill Method: Rotary Wash / Supervisor: REDZA	Date Start : 18.05.2017
Maincon :	INFRA TECH PROJECTS MALAYSIA SDN. BHD.	Casing Type : NW	Date Finish : 24.05.2017

Depth m	Strata Thick-ness	Description of Strata	Log	Sample No		Depth m	Rec Ratio	75	75	75	75	75	75	For <75	N Value	For <300
40																
41		Stiff medium grey CLAY.	---	P27/D26	P	40.500 / 40.950	100%	1	2	2	3	3	2		10	
42		Stiff light grey CLAY.	---	P28/D27	P	42.000 / 42.450	100%	1	2	3	2	2	3		10	
43		Stiff medium grey CLAY.	---	P29/D28	P	43.500 / 43.950	100%	1	2	3	2	3	3		11	
44																
45	45.45	Stiff medium grey CLAY.	---	P30/D29	P	45.000 / 45.450	100%	1	2	2	3	3	3		11	
46		End of BH 8 at 45.45 m depth.														
47–60																

SAMPLING DETAIL — Penetration, P (mm) — SPT BLOW COUNT — Rec = Recovery

SPT PLOT

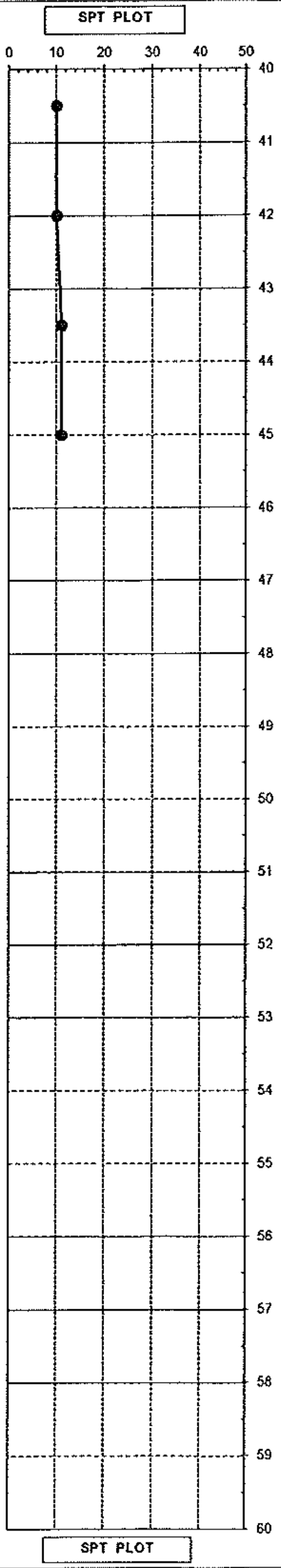

Legend:

- D — Disturbed Sample
- P — Standard Penetration Test
- UD — Undisturbed Sample
- MZ — Mazier Sample
- VS — Vane Shear Test
- C — Rock Coring
- W — Water Sample
- N - No. of Blows/300mm

NOTE:

** Existing ground level

Example:

50 | 120 = 50 Blows/120 mm

Cohesive Soil (N): 0, 2, 4, 8, 15, 30 — V.Soft, Soft, Firm, Stiff, V.Stiff, Hard

Non cohesive Soil (N): 0, 4, 10, 30, 50 — V.Loose, Loose, Med Dense, Dense, V.Dense

HSD ENGINEERING SERVICES

ENGINEERING BOREHOLE LOG

Project : SOIL INVESTIGATION WORKS			Borehole No : **BH 9**
Location : BAGAN DATUK WATER CITY PHASE 1, PERAK DARUL RIDZUAN			Ground Level: 0.159 m
Client : PERBADANAN KEMAJUAN NEGERI PERAK	Rig Type : YWE D-90R	Driller : SHAH	Water Level : 0.57 m
Consultant: INFRA TECH GEO SOULTIONS (M) SDN. BHD.	Drill Method: Rotary Wash	Supervisor: REDZA	Date Start : 14.05.2017
Maincon : INFRA TECH PROJECTS MALAYSIA SDN. BHD.	Casing Type : NW		Date Finish : 17.05.2017

Depth m	Strata Thick-ness	Description of Strata	Log	Sample No	Depth m	Rec Ratio	75	75	75	75	75	75	For <75	N Value	For <300
				SAMPLING DETAIL			Penetration, P (mm) / SPT BLOW COUNT							N	
0	0.30	Top soil			0.300										
1.5		Very soft dark grey CLAY.		P1/D1	1.500<br>1.950	100%	0	0	0	0	0	0		0	
3		Very soft dark grey CLAY.		P2/D2	3.000<br>3.450	100%	0	0	0	0	0	0		0	
4.5		Very soft dark grey CLAY.		P3/D3	4.500<br>4.950	100%	0	0	0	0	0	0		0	
6	7.50	Very soft dark grey sandy CLAY.		P4/D4	6.000<br>6.450	100%	0	0	0	0	0	0		0	
7.5		Dark grey sandy CLAY of intermediate plasticity.		UD 1	7.500<br>8.000	100%									
9		Very soft dark grey sandy CLAY.		P5/D5	9.000<br>9.450	100%	0	0	0	0	0	0		0	
10.5		Dark grey sandy CLAY of Intermediate plasticity.		UD 2	10.500<br>11.000	100%									
12		Very soft dark grey sandy CLAY.		P6/D6	12.000<br>12.450	100%	0	0	1	0	0	1		2	
13.5	15.00	Very soft dark grey sandy CLAY.		P7/D7	13.500<br>13.950	100%	0	1	0	1	0	1		2	
15		Very soft dark grey CLAY.		P8/D8	15.000<br>15.450	100%	0	0	0	0	0	0		0	
16.5	18.00	Dark grey CLAY of high plasticity.		UD 3	16.500<br>17.000	100%									
18	19.50	Very soft dark grey sandy CLAY.		P11/D11	18.000<br>18.450	100%	0	0	0	0	0	0		0	
19.5		Dark grey slightly silty SAND.		UD 4	19.500<br>20.000	100%									
20						Rec = Recovery									

SPT PLOT

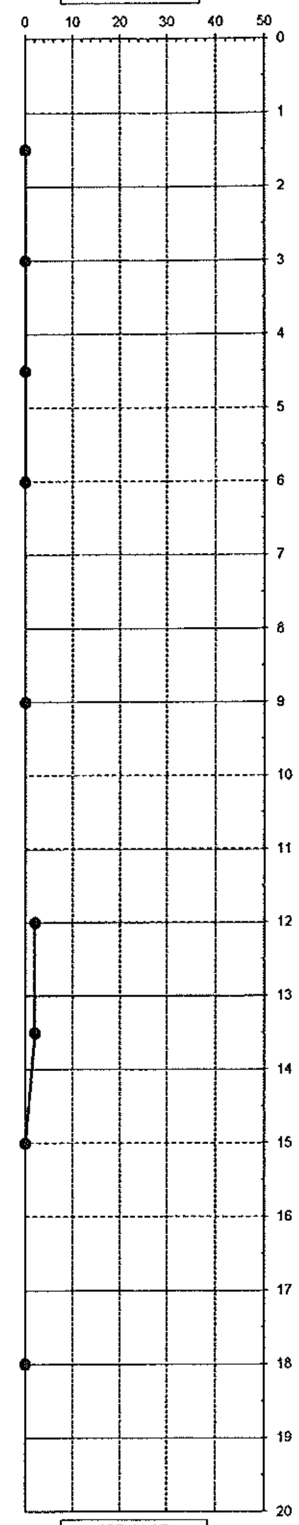

Legend:

- D — Disturbed Sample
- P — Standard Penetration Test
- UD — Undisturbed Sample
- MZ — Mazier Sample
- VS — Vane Shear Test
- C — Rock Coring
- W — Water Sample
- N - No. of Blows/300mm

NOTE:

** Existing ground level

Example:

50 | 120 = 50 Blows/120 mm

Cohesive Soil (N)	0	2	4	8	15	30	
	V.Soft,	Soft,	Firm,	Stiff,	V.Stiff,	Hard	

Non-cohesive Soil (N)	0	4	10	30	50
	V.Loose	Loose	Med Dense	Dense	V.Dense

HSD ENGINEERING SERVICES

ENGINEERING BOREHOLE LOG

Project : SOIL INVESTIGATION WORKS			Borehole No : BH 9
Location : BAGAN DATUK WATER CITY PHASE 1, PERAK DARUL RIDZUAN			Ground Level: 0.159 m
Client : PERBADANAN KEMAJUAN NEGERI PERAK	Rig Type : YWE D-90R	Driller : SHAH	Water Level : 0.57 m
Consultant: INFRA TECH GEO SOULTIONS (M) SDN. BHD.	Drill Method: Rotary Wash	Supervisor: REDZA	Date Start : 14.05.2017
Maincon : INFRA TECH PROJECTS MALAYSIA SDN. BHD.	Casing Type : NW		Date Finish : 17.05.2017

Depth m	Strata Thickness	Description of Strata	Log	Sample No	Depth m	Rec Ratio	75	75	75	75	75	75	For <75	N Value	For <300
20															
21		Very soft dark grey CLAY.		P10/D10	21.000 21.450	100%	0	0	0	0	0	0		0	
22		Very soft dark grey CLAY.		P11/D11	22.500 22.950	100%	0	0	0	0	0	0		0	
23															
24		Very soft dark grey CLAY.		P12/D12	24.000 24.450	100%	0	0	0	0	0	0		0	
25		Very soft dark grey CLAY.		P13/D13	25.500 25.950	100%	0	0	0	0	0	0		0	
26															
27		Very soft dark grey CLAY.		P14/D14	27.000 27.450	100%	0	0	0	0	0	0		0	
28		Very soft dark grey CLAY.		P15/D15	28.500 28.950	100%	0	0	0	0	0	0		0	
29															
30		Very soft medium grey streaked with black CLAY.		P16/D16	30.000 30.450	100%	0	0	0	0	0	0		0	
31		Very soft light grey CLAY.		P17/D17	31.500 31.950	100%	0	0	0	0	0	0		0	
32															
33		Very soft light grey CLAY.		P18/D18	33.000 33.450	100%	0	0	0	0	0	0		0	
34	34.50														
35		Very soft light grey sandy CLAY of intermediate plasticity.		P19/D19	34.500 34.950	100%	0	0	0	0	0	0		0	
36	36.00	Very soft light grey CLAY.		P20/D20	36.000 36.450	100%	3	3	4	7	5	7		23	
37	37.50	Very stiff light grey sandy CLAY with traces of coarse sand.		P21/D21	37.500 37.950	100%	3	4	4	8	5	9		26	
38															
39	39.00	Very soft medium grey streaked with black CLAY.		P22/D22	39.000 39.450	100%	0	0	0	0	0	0		0	
40															

Rec = Recovery

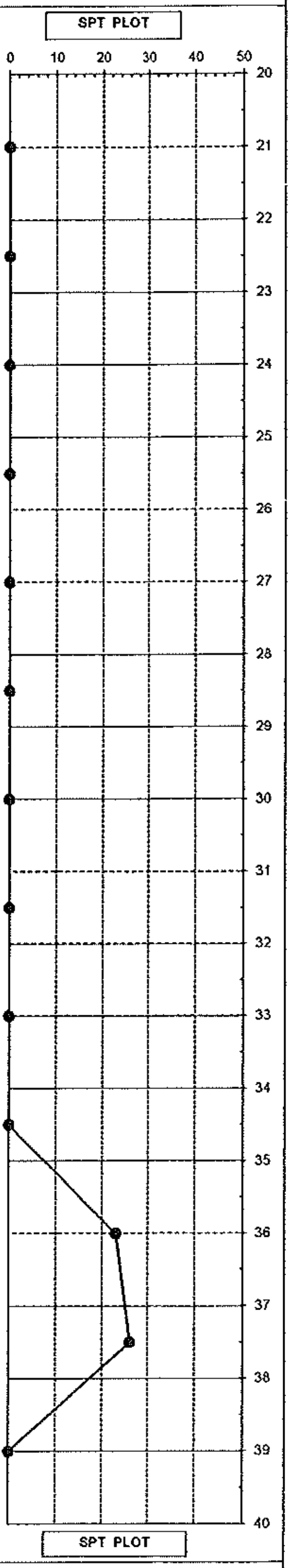

Legend:

- D Disturbed Sample
- P Standard Penetration Test
- UD Undisturbed Sample
- MZ Mazier Sample
- VS Vane Shear Test
- C Rock Coring
- W Water Sample
- N · No. of Blows/300mm

NOTE:

** Existing ground level

Example:

50 | 120 = 50 Blows/120 mm

Cohesive Soil (N): 0, 2, 4, 8, 15, 30 — V.Soft, Soft, Firm, Stiff, V.Stiff, Hard

Non-cohesive Soil (N): 0, 4, 10, 30, 50 — V.Loose, Loose, Med Dense, Dense, V.Dense

HSD ENGINEERING SERVICES

ENGINEERING BOREHOLE LOG

Project : SOIL INVESTIGATION WORKS			Borehole No : BH 9
Location : BAGAN DATUK WATER CITY PHASE 1, PERAK DARUL RIDZUAN			Ground Level: 0.159 m
Client : PERBADANAN KEMAJUAN NEGERI PERAK	Rig Type : YWE D-90R	Driller : SHAH	Water Level : 0.57 m
Consultant: INFRA TECH GEO SOULTIONS (M) SDN. BHD.	Drill Method: Rotary Wash	Supervisor: REDZA	Date Start : 14.05.2017
Maincon : INFRA TECH PROJECTS MALAYSIA SDN. BHD.	Casing Type : NW		Date Finish : 17.05.2017

Depth m	Strata Thickness	Description of Strata	Sample No	Depth m	Rec Ratio	75	75	75	75	75	75	For <75	N Value	For <300
40 – 42	42.00	Very soft dark grey CLAY.	P23/D23	40.500 / 40.950	100%	0	0	0	0	0	0		0	
42 – 43.5	43.50	Very stiff dark grey sandy CLAY.	P24/D24	42.000 / 42.450	100%	3	4	5	5	9	9		28	
43.5 – 45		Very dense medium grey clayey SAND.	P25/D25	43.500 / 43.860	100%	5	6	12	15	23		60	50	210
45 – 45.45	45.45	Very dense medium grey clayey SAND with some gravels.	P26/D26	45.000 / 45.340	71%	7	7	15	27	8		40	50	190
		End of BH 9 at 45.45 m depth.												

Penetration, P (mm) — SPT BLOW COUNT; Rec = Recovery

SPT PLOT

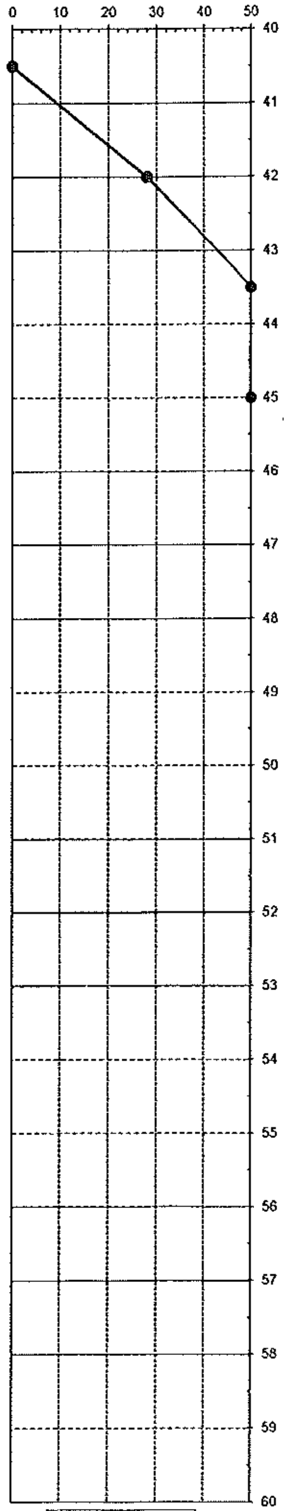

SPT PLOT

Legend:

- D — Disturbed Sample
- P — Standard Penetration Test
- UD — Undisturbed Sample
- MZ — Mazier Sample
- VS — Vane Shear Test
- C — Rock Coring
- W — Water Sample
- N - No. of Blows/300mm

NOTE:

** Existing ground level

Example:

50 | 120 = 50 Blows/120 mm

Cohesive Soil (N)	0	2	4	8	15	30
	V.Soft,	Soft,	Firm,	Stiff,	V.Stiff,	Hard

Non-cohesive Soil (N)	0	4	10	30	50
	V.Loose	Loose	Med Dense	Dense	V.Dense

HSD ENGINEERING SERVICES

ENGINEERING BOREHOLE LOG

Project :	SOIL INVESTIGATION WORKS	Borehole No :	BH 10
Location :	BAGAN DATUK WATER CITY PHASE 1, PERAK DARUL RIDZUAN	Ground Level:	**
Client :	PERBADANAN KEMAJUAN NEGERI PERAK	Rig Type : YWE D-90R	Driller : SHAH
Consultant:	INFRA TECH GEO SOULTIONS (M) SDN. BHD.	Drill Method: Rotary Wash	Supervisor: REOZA
Maincon :	INFRA TECH PROJECTS MALAYSIA SDN. BHD.	Casing Type : NW	
Water Level :	FULL	Date Start :	15.06.2017
Date Finish :	20.06.2017		

Depth m	Strata Thickness	Description of Strata	Sample No	Depth m	Rec Ratio	75	75	75	75	75	75	For <75	N Value	For <300
0	0.30	Top soil.	D	0.300										
1		Very soft grey silty CLAY.	P1/D1	1.500<br>1.950	100%	0	0	0	0	0	0		0	
2														
3		Very soft grey silty CLAY with some seashell.	P2/D2	3.000<br>3.450	100%	0	0	0	0	0	0		0	
			VS 1	3.500										
4	4.50													
		Very soft grey sandy CLAY with some seashell.	P3/D3	4.500<br>4.950	100%	0	0	0	0	0	0		0	
5		Grey sandy CLAY of intermediate plasticity.	UD 1	5.000<br>5.500	100%									
6		Very loose grey clayey fine SAND with some decayed wood and seashell.	P4/D4	6.000<br>6.450	100%	0	0	0	0	0	0		0	
7	7.50													
		Very soft grey CLAY with seashell.	P5/D5	7.500<br>7.950	100%	0	0	0	0	0	0		0	
8			VS 2	8.000										
9		Very soft grey CLAY with seashell.	P6/D6	9.000<br>9.450	100%	0	0	0	0	0	0		0	
		Grey CLAY of very high plasticity.	UD 2	9.500<br>10.000	100%									
10		Very soft grey CLAY with seashell.	P7/D7	10.500<br>10.950	100%	0	0	0	0	0	0		0	
11			VS 3	11.000										
12		Very soft grey CLAY.	P8/D8	12.000<br>12.450	100%	0	0	0	0	0	0		0	
13		Very soft grey CLAY with seashell.	P9/D9	13.500<br>13.950	100%	0	0	0	0	0	0		0	
14		Grey CLAY of extremely high plasticity.	UD 3	14.000<br>14.500	100%									
	15.00													
15		Very soft grey sandy CLAY. Sand is fine grained.	P10/D10	15.000<br>15.450	100%	0	0	0	0	0	0		0	
16	16.50													
		Very soft grey CLAY with lenses of fine grained sand.	P11/D11	16.500<br>16.950	100%	0	0	0	0	0	0		0	
17			VS 4	17.000										
18		Very soft grey CLAY with lenses of fine grained sand.	P12/D12	18.000<br>18.450	100%	0	0	0	0	0	0		0	
		Grey CLAY of extremely high plasticity.	UD 4	18.500<br>19.000	100%									
19		Very soft grey CLAY with some decayed wood.	P13/D13	19.500<br>19.950	100%	0	0	0	0	0	0		0	
20														

Rec = Recovery

SPT PLOT

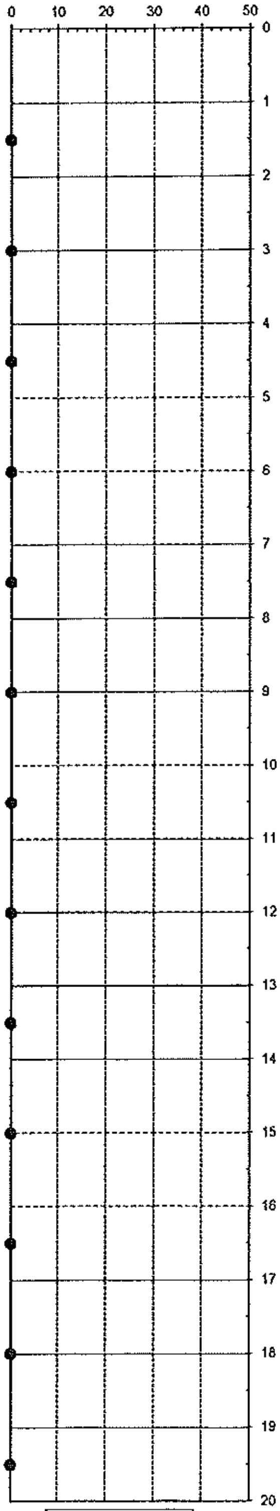

SPT PLOT

Legend:

- D — Disturbed Sample
- P — Standard Penetration Test
- UD — Undisturbed Sample
- MZ — Mazier Sample
- VS — Vane Shear Test
- C — Rock Coring
- W — Water Sample
- N - No. of Blows/300mm

NOTE:

** Existing ground level

Example:

50 | 120 = 50 Blows/120 mm

Cohesive Soil (N)	0	2	4	8	15	30	
	V.Soft,	Soft,	Firm,	Stiff,	V.Stiff,	Hard	
Non-cohesive Soil (N)	0	4	10	30	50		
	V.Loose	Loose	Med Dense	Dense	V.Dense		

HSD ENGINEERING SERVICES

ENGINEERING BOREHOLE LOG

Project :	SOIL INVESTIGATION WORKS	Borehole No :	BH 10
Location :	BAGAN DATUK WATER CITY PHASE 1, PERAK DARUL RIDZUAN	Ground Level:	**
Client :	PERBADANAN KEMAJUAN NEGERI PERAK	Rig Type : YWE D-90R	Driller : SHAH
Consultant:	INFRA TECH GEO SOULTIONS (M) SDN. BHD.	Drill Method: Rotary Wash	Supervisor: REDZA
Maincon :	INFRA TECH PROJECTS MALAYSIA SDN. BHD.	Casing Type : NW	
Water Level :	FULL	Date Start :	15.06.2017
Date Finish :	20.06.2017		

Depth m	Strata Thick-ness	Description of Strata	Log	Sampling Detail: Sample No	Depth m	Rec Ratio	Penetration, P (mm) 75	75	75	75	75	75	For <75	N Value	For <300
20				VS 5	20.000										
21		Very soft grey silty CLAY with some decayed wood.		P14/D14	21.000 21.450	100%	0	0	0	0	0	0		0	
22		Very soft grey silty CLAY with some decayed wood.		P15/D15	22.500 22.950	100%	0	0	0	0	0	0		0	
24		Very soft grey silty CLAY with some decayed wood.		P16/D16	24.000 24.450	100%	0	0	0	0	0	0		0	
25		Very soft grey silty CLAY with some decayed wood.		P17/D17	25.500 25.950	100%	0	0	0	0	0	0		0	
27		Very soft grey silty CLAY with some decayed wood.		P18/D18	27.000 27.450	100%	0	0	0	0	0	0		0	
28		Very soft grey silty CLAY with some decayed wood.		P19/D19	28.500 28.950	100%	0	0	0	0	0	0		0	
30		Very soft grey silty CLAY with seashell.		P20/D20	30.000 30.450	100%	0	0	0	0	0	0		0	
31		Very soft light grey silty CLAY.		P21/D21	31.500 31.950	100%	0	0	0	0	0	0		0	
33		Very soft light grey silty CLAY with lenses of fine grained sand.		P22/D22	33.000 33.450	100%	0	0	0	0	0	0		0	
34	34.50	Dense light grey silty fine SAND		P23/D23	34.500 34.950	67%	6	6	8	7	8	12		35	
36		Medium dense light grey silty fine to medium grained SAND.		P24/D24	36.000 36.450	67%	1	0	2	6	8	11		27	
37	37.50	Very soft light grey silty CLAY with lenses of fine to medium grained sand interbedded with silty CLAY.		P25/D25	37.500 37.950	100%	0	0	0	0	0	0		0	
39		Very soft dark grey silty CLAY with lenses of fine grained sand.		P26/D26	39.000 39.450	100%	0	0	0	0	0	0		0	
40															

Rec = Recovery

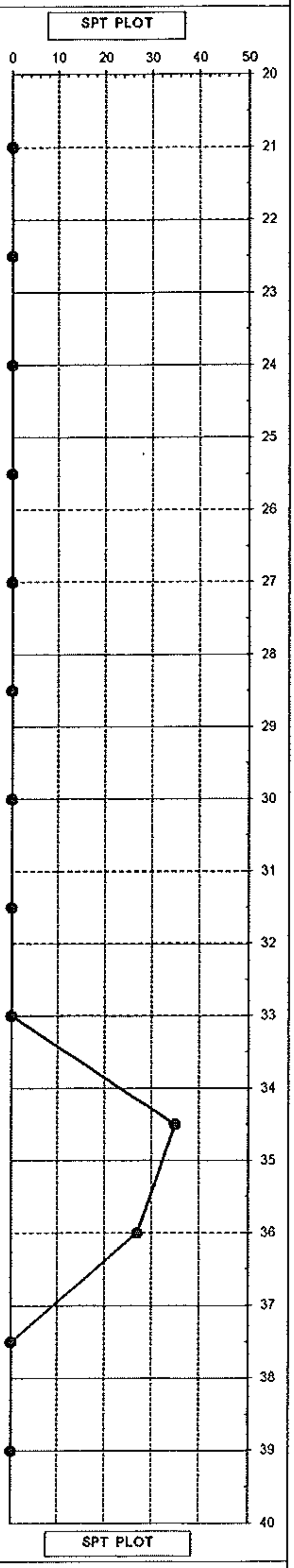

Legend:

- D Disturbed Sample
- P Standard Penetration Test
- UD Undisturbed Sample
- MZ Mazier Sample
- VS Vane Shear Test
- C Rock Coring
- W Water Sample
- N - No. of Blows/300mm

NOTE:

** Existing ground level

Example:

50 | 120 = 50 Blows/120 mm

Cohesive Soil (N)	0	2	4	8	15	30	
	V.Soft	Soft	Firm	Stiff	V.Stiff	Hard	
Non-cohesive Soil (N)	0	4	10	30	50		
	V.Loose	Loose	Med Dense	Dense	V.Dense		

HSD ENGINEERING SERVICES

ENGINEERING BOREHOLE LOG

Project : SOIL INVESTIGATION WORKS			Borehole No : BH 10
Location : BAGAN DATUK WATER CITY PHASE 1, PERAK DARUL RIDZUAN			Ground Level: **
Client : PERBADANAN KEMAJUAN NEGERI PERAK	Rig Type : YWE D-90R	Driller : SHAH	Water Level : FULL
Consultant: INFRA TECH GEO SOULTIONS (M) SDN. BHD.	Drill Method: Rotary Wash	Supervisor: REDZA	Date Start : 15.06.2017
Maincon : INFRA TECH PROJECTS MALAYSIA SDN. BHD.	Casing Type : NW		Date Finish : 20.06.2017

Depth m	Strata Thickness	Description of Strata	Sample No	Depth m	Rec Ratio	75	75	75	75	75	75	For <75	N Value	For <300
40														
40.5		Very soft dark grey silty CLAY with decayed wood interbedded with silty CLAY.	P27/D27	40.500 / 40.950	100%	0	0	0	0	0	0		0	
42		Very soft light grey silty CLAY with traces of fine grained sand.	P28/D28	42.000 / 42.450	100%	0	0	0	0	0	0		0	
43.5	43.50	Medium dense light grey silty fine to coarse SAND and some gravels.	P29/D29	43.500 / 43.950	91%	2	5	5	6	6	6		23	
45	45.00	Medium dense light grey fine to coarse SAND with some gravels.	P30/D30	45.000 / 45.450	100%	2	3	4	5	8	7		24	
48	48.00	Very stiff light grey silty CLAY with lenses of fine grained sand interbedded with decayed wood.	P31/D31	48.000 / 48.450	100%	2	4	3	5	5	6		19	
51	51.00	Stiff light grey sandy CLAY. Sand is fine to medium corase grained.	P32/D32	51.000 / 51.450	100%	1	2	2	2	2	3		9	
54	54.00	Firm to stiff light grey silty CLAY.	P33/D33	54.000 / 54.450	100%	1	2	2	2	2	2		8	
57	57.00	Very loose light grey to milky white clayey fine to coarse grained SAND.	P34/D34	57.000 / 57.450	100%	0	0	0	0	0	0		0	
60														

Rec = Recovery

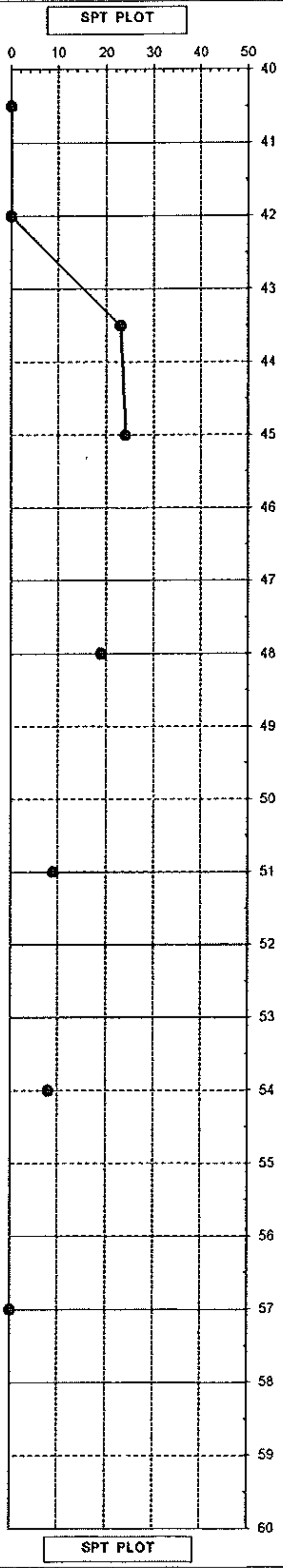

Legend:

- D – Disturbed Sample
- P – Standard Penetration Test
- UD – Undisturbed Sample
- MZ – Mazier Sample
- VS – Vane Shear Test
- C – Rock Coring
- W – Water Sample
- N - No. of Blows/300mm

NOTE:

** Existing ground level

Example:

50 | 120 = 50 Blows/120 mm

Cohesive Soil (N): 0, 2, 4, 8, 15, 30 — V.Soft, Soft, Firm, Stiff, V.Stiff, Hard

Non-cohesive Soil (N): 0, 4, 10, 30, 50 — V.Loose, Loose, Med Dense, Dense, V.Dense

HSD ENGINEERING SERVICES

ENGINEERING BOREHOLE LOG

Sheet 4 Of 4

Project : SOIL INVESTIGATION WORKS			Borehole No : BH 10
Location : BAGAN DATUK WATER CITY PHASE 1, PERAK DARUL RIDZUAN			Ground Level: **
Client : PERBADANAN KEMAJUAN NEGERI PERAK	Rig Type : YWE D-90R	Driller : SHAH	Water Level : FULL
Consultant: INFRA TECH GEO SOULTIONS (M) SDN. BHD.	Drill Method: Rotary Wash	Supervisor: REDZA	Date Start : 15.06.2017
Maincon : INFRA TECH PROJECTS MALAYSIA SDN. BHD.	Casing Type : NW		Date Finish : 20.06.2017

Depth m	Strata Thickness	Description of Strata	Log	Sample No	Depth m	Rec Ratio	75	75	75	75	75	75	For <75	N Value	For <300
60															
	60.45	Medium dense light grey silty fine to coarse SAND with decayed wood and some gravels.		P35/D35	60.000<br>60.450	100%	1	3	2	3	4	5		14	
61		End of BH 10 at 60.45 m depth.													

Depth scale: 60 – 80 m. Penetration, P (mm) / SPT BLOW COUNT. Rec = Recovery

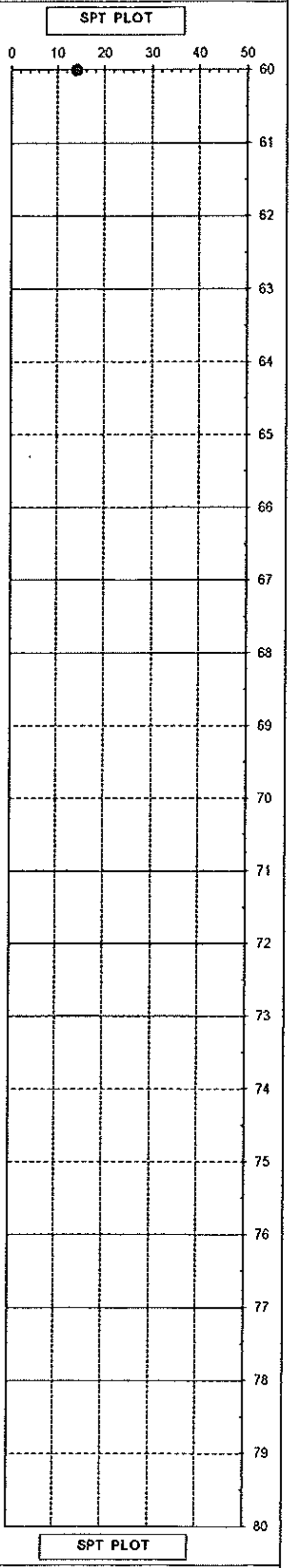

Legend:

- D — Disturbed Sample
- P — Standard Penetration Test
- UD — Undisturbed Sample
- MZ — Mazier Sample
- VS — Vane Shear Test
- C — Rock Coring
- W — Water Sample
- N - No. of Blows/300mm

NOTE:

** Existing ground level

Example:

50 | 120 = 50 Blows/120 mm

Cohesive Soil (N)	0	2	4	8	15	30	
	V.Soft	Soft	Firm	Stiff	V.Stiff	Hard	
Non-cohesive Soil (N)	0	4	10	30	50		
	V.Loose	Loose	Med Dense	Dense	V.Dense		

HSD ENGINEERING SERVICES

ENGINEERING BOREHOLE LOG

Project : SOIL INVESTIGATION WORKS			Borehole No : BH 11
Location : BAGAN DATUK WATER CITY PHASE 1, PERAK DARUL RIDZUAN			Ground Level: 0.238 m
Client : PERBADANAN KEMAJUAN NEGERI PERAK	Rig Type : YWE D-90R	Driller : SHAH	Water Level : 0.50 m
Consultant: INFRA TECH GEO SOULTIONS (M) SDN. BHD.	Drill Method: Rotary Wash	Supervisor: REDZA	Date Start : 30.05.2017
Maincon : INFRA TECH PROJECTS MALAYSIA SDN. BHD.	Casing Type : NW		Date Finish : 02.06.2017

Depth m	Strata Thickness	Description of Strata	Log	Sample No	Depth m	Rec Ratio	75	75	75	75	75	75	For <75	N Value	For <300
0	0.30	Top soil.		D	0.300										
1		Very soft dark grey CLAY with some organic material.		P1/D1	1.500<br>1.950	100%	0	0	0	0	0	0		0	
2		Dark grey CLAY of intermediate plasticity.		UD 1	4.000<br>4.500	100%									
3		Very soft dark grey CLAY with some organic material.		P2/D2	3.000<br>3.450	100%	0	0	0	0	0	0		0	
4–5	6.00	Very soft dark grey CLAY with lenses of sand and organic material.		P3/D3	4.500<br>4.950	100%	0	0	0	0	0	0		0	
6		Very soft dark grey sandy CLAY.		P4/D4	6.000<br>6.450	100%	0	0	0	0	0	0		0	
7	8.00	Very soft dark grey sandy CLAY with some organic material.		P5/D5	7.500<br>7.950	100%	0	1	0	0	0	0		0	
8		Dark grey CLAY of high plasticity.		UD 2	8.000<br>8.500	100%									
9		Very soft dark grey CLAY.		P6/D6	9.000<br>9.450	100%	0	0	0	0	0	0		0	
10		Very soft dark grey CLAY with lenses of fine grained sand.		P7/D7	10.500<br>10.950	100%	0	0	0	0	0	0		0	
11	12.00	Dark grey CLAY of very high plasticity.		UD 3	11.000<br>11.500	100%									
12		Very soft dark grey sandy CLAY with some seashell.		P8/D8	12.000<br>12.450	100%	0	0	0	0	0	0		0	
13–14		Very soft dark grey sandy CLAY with some seashell.		P9/D9	13.500<br>13.950	100%	0	0	0	0	0	0		0	
15	15.50	Very soft dark grey sandy CLAY with some seashell.		P10/D10	15.000<br>15.450	100%	0	0	0	0	0	0		0	
16		Dark grey CLAY of extremely high plasticity.		UD 4	15.500<br>16.000	100%									
17		Very soft dark grey CLAY interbedded with fine to medium coarse sand and seashell.		P11/D11	16.500<br>16.950	100%	0	0	0	0	0	0		0	
18		Very soft dark grey CLAY with some seashell.		P12/D12	18.000<br>18.450	100%	0	0	1	0	0	0		1	
19–20		Very soft dark grey CLAY with some decayed wood.		P13/D13	19.500<br>19.950	100%	0	0	0	0	0	0		0	

Rec = Recovery

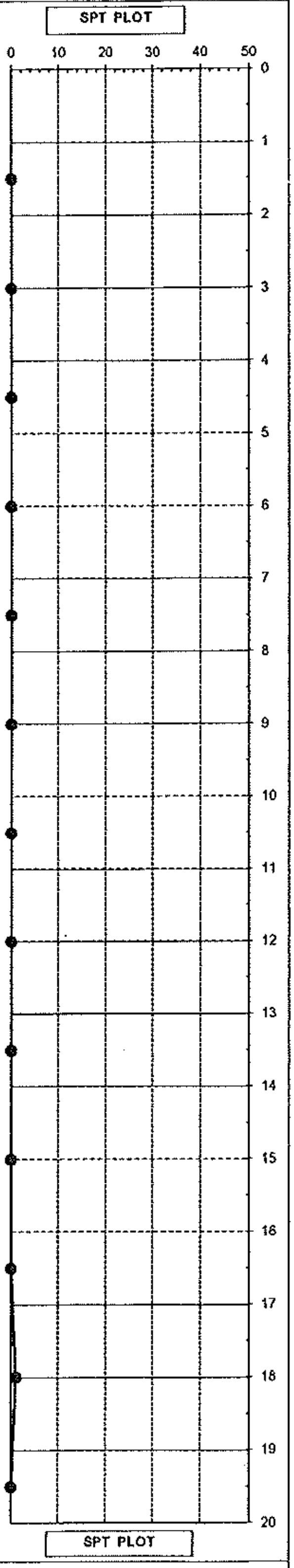

Legend:

- D — Disturbed Sample
- P — Standard Penetration Test
- UD — Undisturbed Sample
- MZ — Mazier Sample
- VS — Vane Shear Test
- C — Rock Coring
- W — Water Sample
- N - No. of Blows/300mm

NOTE:

** Existing ground level

Example:

50 | 120 = 50 Blows/120 mm

Cohesive Soil (N)	0	2	4	8	15	30
	V.Soft	Soft	Firm	Stiff	V.Stiff	Hard
Non-cohesive Soil (N)	0	4	10	30	50	
	V.Loose	Loose	Med Dense	Dense	V.Dense	

HSD ENGINEERING SERVICES

ENGINEERING BOREHOLE LOG

Project :	SOIL INVESTIGATION WORKS	Borehole No :	**BH 11**
Location :	BAGAN DATUK WATER CITY PHASE 1, PERAK DARUL RIDZUAN	Ground Level:	0.238 m
Client :	PERBADANAN KEMAJUAN NEGERI PERAK	Water Level :	0.50 m
Consultant:	INFRA TECH GEO SOULTIONS (M) SDN. BHD.	Date Start :	30.05.2017
Maincon :	INFRA TECH PROJECTS MALAYSIA SDN. BHD.	Date Finish :	02.06.2017

Rig Type :	YWE D-90R	Driller :	SHAH
Drill Method:	Rotary Wash	Supervisor:	REDZA
Casing Type :	NW		

Depth m	Strata Thick-ness	Description of Strata	Log	Sample No	Depth m	Rec Ratio	75	75	75	75	75	75	For <75	N Value	For <300
20		Dark grey CLAY of very high plasticity.		UD 5	20.000 20.500	100%									
21		Very soft dark grey CLAY with some decayed wood.		P14/D14	21.000 21.450	100%	0	0	0	0	0	0		0	
22		Very soft dark grey CLAY with some decayed wood.		P15/D15	22.500 22.950	100%	0	0	0	0	0	0		0	
24		Very soft dark grey silty CLAY with some decayed wood.		P16/D16	24.000 24.450	100%	0	0	0	0	0	0		0	
25	25.50														
		Very soft dark grey silty CLAY with some decayed wood.		P17/D17	25.500 25.950	100%	0	0	0	0	0	0		0	
27		Very soft dark grey silty CLAY with some decayed wood.		P18/D18	27.000 27.450	100%	0	0	0	0	0	0		0	
28		Very soft dark grey silty CLAY with some decayed wood.		P19/D19	28.500 28.950	100%	0	0	0	0	0	0		0	
30		Very soft dark grey silty CLAY with lenses of fine grained sand and decayed wood.		P20/D20	30.000 30.450	100%	0	0	0	0	0	0		0	
31		Very soft dark grey silty CLAY with some decayed wood.		P21/D21	31.500 31.950	100%	0	0	0	0	0	0		0	
33		Firm light grey to brownish silty CLAY with lenses of fine to medium coarse sand and organic material.		P22/D22	33.000 33.450	100%	0	0	1	2	1	2		6	
34		Firm greenish grey to brownish silty CLAY with some organic material.		P23/D23	34.500 34.950	100%	0	0	1	1	1	1		4	
36		Soft to firm light grey silty CLAY with lenses of fine sand and decayed wood.		P24/D24	36.000 36.450	100%	0	0	1	1	1	1		4	
37		Firm light grey silty CLAY interbedded with fine to coarse sand.		P25/D25	37.500 37.950	100%	1	3	2	2	2	1		7	
39		Soft to firm dark grey silty CLAY with decayed wood.		P26/D26	39.000 39.450	100%	1	0	1	1	1	1		4	
40															

Rec = Recovery

SPT PLOT

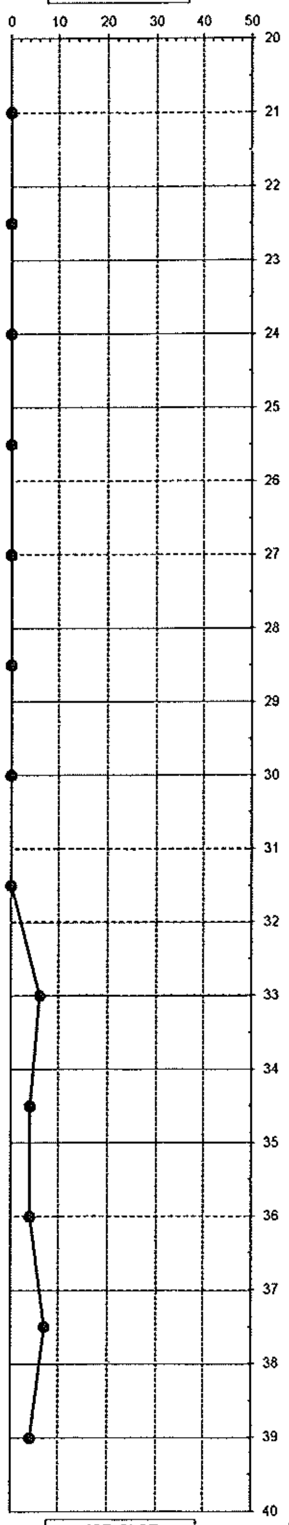

Legend:

- D Disturbed Sample
- P Standard Penetration Test
- UD Undisturbed Sample
- MZ Mazier Sample
- VS Vane Shear Test
- C Rock Coring
- W Water Sample
- N - No. of Blows/300mm

NOTE:

** Existing ground level

Example:

50 | 120 = 50 Blows/120 mm

Cohesive Soil (N): 0, 2, 4, 8, 15, 30 — V.Soft, Soft, Firm, Stiff, V.Stiff, Hard

Non-cohesive Soil (N): 0, 4, 10, 30, 50 — V.Loose, Loose, Med Dense, Dense, V.Dense

HSD ENGINEERING SERVICES

ENGINEERING BOREHOLE LOG

Project : SOIL INVESTIGATION WORKS			Borehole No : **BH 11**
Location : BAGAN DATUK WATER CITY PHASE 1, PERAK DARUL RIDZUAN			Ground Level: 0.238 m
Client : PERBADANAN KEMAJUAN NEGERI PERAK	Rig Type : YWE D-90R	Driller : SHAH	Water Level : 0.50 m
Consultant: INFRA TECH GEO SOULTIONS (M) SDN. BHD.	Drill Method: Rotary Wash	Supervisor: REDZA	Date Start : 30.05.2017
Maincon : INFRA TECH PROJECTS MALAYSIA SDN. BHD.	Casing Type : NW		Date Finish : 02.06.2017

Depth m	Strata Thick-ness	Description of Strata	Log	Sample No	Depth m	Rec Ratio	75	75	75	75	75	75	For <75	N Value	For <300
40															
41	42.00	Soft light grey silty CLAY with decayed wood.		P27/D27	40.500<br>40.950	100%	0	0	0	0	1	2		3	
42–43	43.50	Very stiff light grey sandy CLAY with some sand.		P28/D28	42.000<br>42.450	0%	1	2	3	4	7	6		20	
44		Medium dense light grey fine to coarse SAND.		P29/D29	43.500<br>43.950	58%	1	3	3	4	6	6		19	
45	45.45	Medium dense light grey medium to coarse SAND.		P30/D30	45.000<br>45.450	49%	0	0	4	5	7	6		22	
46		End of BH 11 at 45.45 m depth.													

Penetration, P (mm) — SPT BLOW COUNT; SAMPLING DETAIL; Rec = Recovery

SPT PLOT

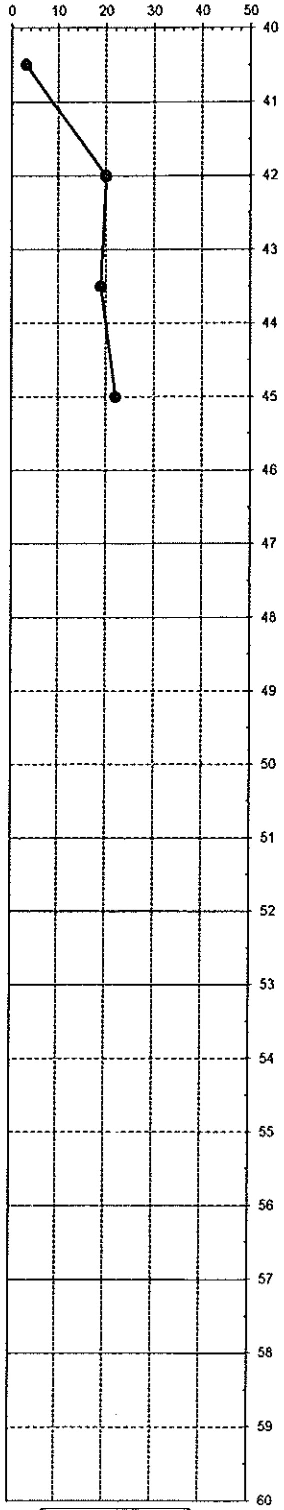

SPT PLOT

Legend:

- D – Disturbed Sample
- P – Standard Penetration Test
- UD – Undisturbed Sample
- MZ – Mazier Sample
- VS – Vane Shear Test
- C – Rock Coring
- W – Water Sample
- N - No. of Blows/300mm

NOTE:

** Existing ground level

Example:

50 | 120 = 50 Blows/120 mm

Cohesive Soil (N): 0, 2, 4, 8, 15, 30 — V.Soft, Soft, Firm, Stiff, V.Stiff, Hard

Non-cohesive Soil (N): 0, 4, 10, 30, 50 — V.Loose, Loose, Med Dense, Dense, V.Dense

HSD ENGINEERING SERVICES

ENGINEERING BOREHOLE LOG

Project :	SOIL INVESTIGATION WORKS	Borehole No :	BH 12
Location :	BAGAN DATUK WATER CITY PHASE 1, PERAK DARUL RIDZUAN	Ground Level:	0.098 m
Client :	PERBADANAN KEMAJUAN NEGERI PERAK	Rig Type : YWE D-90R	Driller : SHAH
Consultant:	INFRA TECH GEO SOULTIONS (M) SDN. BHD.	Drill Method: Rotary Wash	Supervisor: REDZA
Maincon :	INFRA TECH PROJECTS MALAYSIA SDN. BHD.	Casing Type : NW	
Water Level :	FULL	Date Start :	07.06.2017
Date Finish :	08.06.2017		

Depth m	Strata Thickness	Description of Strata	Log	Sample No	Depth m	Rec Ratio	75	75	75	75	75	75	For <75	N Value	For <300
0	0.30	Top soil.		D	0.300										
1–2		Very soft grey CLAY.		P1/D1	1.500<br>1.950	100%	0	0	0	0	0	0		0	
3		Very soft grey CLAY.		P2/D2	3.000<br>3.450	100%	0	0	0	0	0	0		0	
4–5		Very soft grey CLAY with lenses of fine grained sand and decayed wood.		P3/D3	4.500<br>4.950	100%	0	0	0	0	0	0		0	
5		Grey CLAY of high plasticity.		UD 1	5.000<br>5.500	100%									
6		Very soft grey CLAY with lenses of fine grained sand		P4/D4	6.000<br>6.450	100%	0	0	0	0	0	0		0	
7–8		Very soft grey CLAY with lenses of fine grained sand.		P5/D5	7.500<br>7.950	100%	0	0	0	0	0	0		0	
9		Very soft grey CLAY with lenses of fine grained sand and seashell.		P6/D6	9.000<br>9.450	100%	0	0	0	0	0	0		0	
10		Grey CLAY of very high plasticity.		UD 2	9.500<br>10.000	100%									
11		Very soft grey CLAY with lenses of fine grained sand.		P7/D7	10.500<br>10.950	100%	0	0	0	0	0	0		0	
12		Very soft grey CLAY with some seashell.		P8/D8	12.000<br>12.450	100%	0	0	0	0	0	0		0	
13–14		Very soft grey CLAY with some seashell.		P9/D9	13.500<br>13.950	100%	0	0	0	0	0	0		0	
14	15.00	Grey CLAY of intermediate plasticity.		UD 3	14.000<br>14.500	100%									
15–16	16.50	Very loose light grey silty fine SAND with some seashell.		P10/D10	15.000<br>15.450	100%	0	0	0	0	0	0		0	
17	18.00	No recovery.		P11	16.500<br>16.950	100%	0	0	0	0	0	0		0	
18		Very soft grey CLAY with lenses of fine to medium coarse sand.		P12/D11	18.000<br>18.450	100%	0	0	0	0	0	0		0	
19	19.50	Grey CLAY of intermediate plasticity.		UD 4	18.500<br>19.000	100%									
20		Blackish decayed wood.		P13/D12	19.500<br>19.950	100%	1	1	1	1	2	1		5	

Rec = Recovery

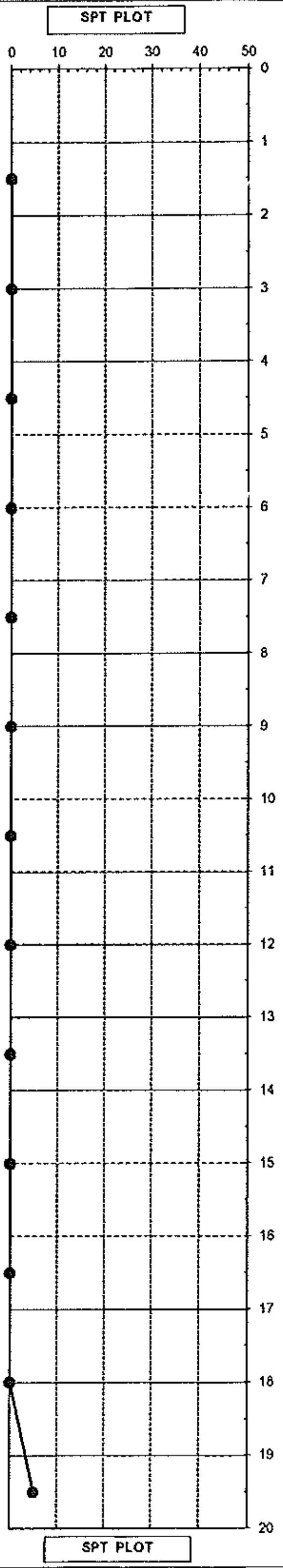

Legend:

- D — Disturbed Sample
- P — Standard Penetration Test
- UD — Undisturbed Sample
- MZ — Mazier Sample
- VS — Vane Shear Test
- C — Rock Coring
- W — Water Sample
- N - No. of Blows/300mm

NOTE:

** Existing ground level

Example:

50 | 120 = 50 Blows/120 mm

Cohesive Soil (N): 0 2 4 8 15 30 — V.Soft, Soft, Firm, Stiff, V.Stiff, Hard

Non-cohesive Soil (N): 0 4 10 30 50 — V.Loose Loose Med Dense Dense V.Dense

HSD ENGINEERING SERVICES

ENGINEERING BOREHOLE LOG

Project : SOIL INVESTIGATION WORKS			Borehole No : BH 12
Location : BAGAN DATUK WATER CITY PHASE 1, PERAK DARUL RIDZUAN			Ground Level: 0.098 m
Client : PERBADANAN KEMAJUAN NEGERI PERAK	Rig Type : YWE D-90R	Driller : SHAH	Water Level : FULL
Consultant: INFRA TECH GEO SOULTIONS (M) SDN. BHD.	Drill Method: Rotary Wash	Supervisor: REDZA	Date Start : 07.06.2017
Maincon : INFRA TECH PROJECTS MALAYSIA SDN. BHD.	Casing Type : NW		Date Finish : 08.06.2017

Depth m	Strata Thickness	Description of Strata	Log	Sample No	Depth m	Rec Ratio	75	75	75	75	75	75	For <75	N Value	For <300
20	21.00	Grey sandy CLAY of intermediate plasticity.		UD 5	20.000 20.500	100%									
21		Very soft grey silty CLAY with some decayed wood.		P14/D13	21.000 21.450	100%	0	0	0	0	0	0		0	
22		Very soft grey silty CLAY with some decayed wood.		P15/D14	22.500 22.950	100%	1	0	0	0	1	0		1	
24		Very soft grey silty CLAY with some decayed wood.		P16/D15	24.000 24.450	100%	0	0	0	0	0	0		0	
25		Very soft grey silty CLAY with some decayed wood.		P17/D16	25.500 25.950	100%	0	0	0	0	0	0		0	
27		Very soft grey silty CLAY with some decayed wood.		P18/D17	27.000 27.450	100%	0	0	0	0	0	0		0	
28		Very soft grey silty CLAY with some decayed wood.		P19/D18	28.500 28.950	100%	0	0	0	0	0	0		0	
30		Very soft grey silty CLAY with some decayed wood.		P20/D19	30.000 30.450	100%	0	0	0	0	0	0		0	
31	33.00	Very soft grey silty CLAY with lenses of fine grained sand and interbedded with silty clay and some decayed wood.		P21/D20	31.500 31.950	100%	0	0	0	0	0	0		0	
33	33.45	No recovery.		P22	33.000 33.450	0%	1	2	3	4	3	2		12	

Borehole terminated at 33.45 m due to methane gas flow out from the borehole. The possibility of continue drilling without proper borehole treatment may tend to high risk work.

* Borehole offset 10 m form the existing location.

Rec = Recovery

SPT PLOT

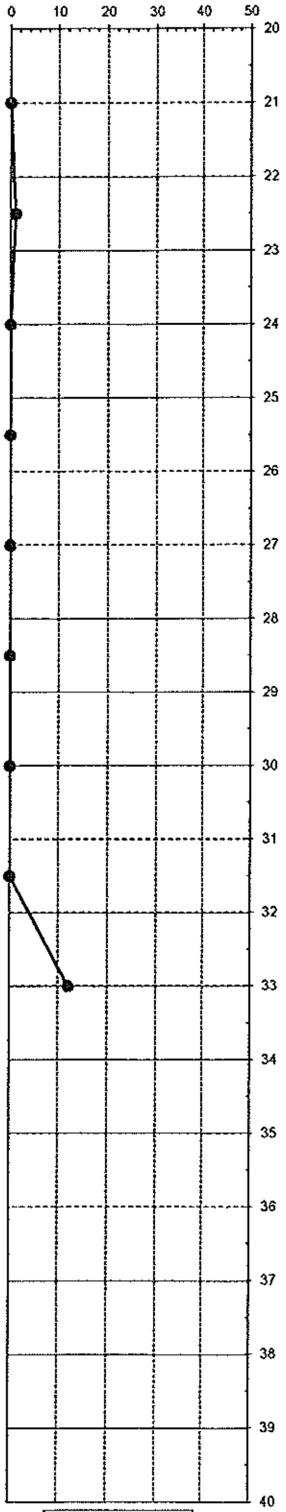

Legend:

- D – Disturbed Sample
- P – Standard Penetration Test
- UD – Undisturbed Sample
- MZ – Mazier Sample
- VS – Vane Shear Test
- C – Rock Coring
- W – Water Sample
- N – No. of Blows/300mm

NOTE:

** Existing ground level

Example:

50 | 120 = 50 Blows/120 mm

Cohesive Soil (N): 0, 2, 4, 8, 15, 30 — V.Soft, Soft, Firm, Stiff, V.Stiff, Hard

Non-cohesive Soil (N): 0, 4, 10, 30, 50 — V.Loose, Loose, Med Dense, Dense, V.Dense

HSD ENGINEERING SERVICES

ENGINEERING BOREHOLE LOG

Project :	SOIL INVESTIGATION WORKS	Borehole No :	BH 12 A
Location :	BAGAN DATUK WATER CITY PHASE 1, PERAK DARUL RIDZUAN	Ground Level:	0.098 m
Client :	PERBADANAN KEMAJUAN NEGERI PERAK	Rig Type : YWE D-90R	Driller : SHAH
Consultant:	INFRA TECH GEO SOULTIONS (M) SDN. BHD.	Drill Method: Rotary Wash	Supervisor: REDZA
Maincon :	INFRA TECH PROJECTS MALAYSIA SDN. BHD.	Casing Type : NW	

Water Level : 0.60 m
Date Start : 09.06.2017
Date Finish : 14.06.2017

Depth m	Strata Thickness	Description of Strata	Log	Sample No	Depth m	Rec Ratio	SPT Blow Count 75	75	75	75	75	75	For <75	N Value	For <300
0 – 20		Wash boring from 0.00 m until 34.50 m.		D	0.150										

SPT PLOT (N: 0, 10, 20, 30, 40, 50; Depth 0–20 m)

Rec = Recovery

Legend:

- D – Disturbed Sample
- P – Standard Penetration Test
- UD – Undisturbed Sample
- MZ – Mazier Sample
- VS – Vane Shear Test
- C – Rock Coring
- W – Water Sample
- N - No. of Blows/300mm

NOTE:

** Existing ground level

Example:
50 | 120 = 50 Blows/120 mm

Cohesive Soil (N): 0, 2, 4, 8, 15, 30 — V.Soft, Soft, Firm, Stiff, V.Stiff, Hard

Non-cohesive Soil (N): 0, 4, 10, 30, 50 — V.Loose, Loose, Med Dense, Dense, V.Dense

HSD ENGINEERING SERVICES

ENGINEERING BOREHOLE LOG

Project :	SOIL INVESTIGATION WORKS	Borehole No :	BH 12 A
Location :	BAGAN DATUK WATER CITY PHASE 1, PERAK DARUL RIDZUAN	Ground Level:	0.098 m
Client :	PERBADANAN KEMAJUAN NEGERI PERAK	Water Level :	0.60 m
Consultant:	INFRA TECH GEO SOULTIONS (M) SDN. BHD.	Date Start :	09.06.2017
Maincon :	INFRA TECH PROJECTS MALAYSIA SDN. BHD.	Date Finish :	14.06.2017

Rig Type : YWE D-90R | Drill Method: Rotary Wash | Casing Type : NW

Driller : SHAH | Supervisor: REDZA

Depth m	Strata Thickness	Description of Strata	Log	Sample No		Depth m	Rec Ratio	75	75	75	75	75	75	For <75	N Value	For <300
20 – 34.50		Wash boring from 0.00 m until 34.50 m.														
	34.50															
34.50		Very soft grey silty CLAY with some organic material.		P23/D21	P	34.500 34.950	100%	0	0	0	0	0	0		0	
36		Very soft grey silty CLAY		P24/D22	P	36.000 36.450	100%	0	0	0	0	0	0		0	
37.5		Soft grey silty CLAY with some organic material.		P25/D23	P	37.500 37.950	100%	0	0	0	1	1	1		3	
39		Very soft to soft dark grey silty CLAY with some organic material.		P26/D24	P	39.000 39.450	100%	0	0	0	1	0	1		2	

Rec = Recovery

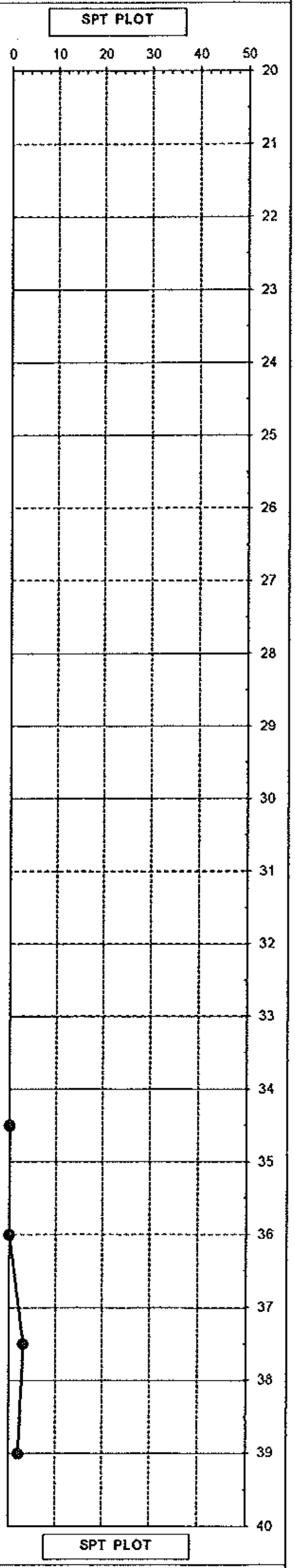

Legend:

- D – Disturbed Sample
- P – Standard Penetration Test
- UD – Undisturbed Sample
- MZ – Mazier Sample
- VS – Vane Shear Test
- C – Rock Coring
- W – Water Sample
- N - No. of Blows/300mm

NOTE:

** Existing ground level

Example:

50 | 120 = 50 Blows/120 mm

Cohesive Soil (N)	0	2	4	8	15	30
	V.Soft	Soft	Firm	Stiff	V.Stiff	Hard

Non-cohesive Soil (N)	0	4	10	30	50
	V.Loose	Loose	Med Dense	Dense	V.Dense

HSD ENGINEERING SERVICES

ENGINEERING BOREHOLE LOG

Project :	SOIL INVESTIGATION WORKS	Borehole No :	BH 12 A
Location :	BAGAN DATUK WATER CITY PHASE 1, PERAK DARUL RIDZUAN	Ground Level:	0.098 m
Client :	PERBADANAN KEMAJUAN NEGERI PERAK	Water Level :	0.60 m
Consultant:	INFRA TECH GEO SOULTIONS (M) SDN. BHD.	Date Start :	09.06.2017
Maincon :	INFRA TECH PROJECTS MALAYSIA SDN. BHD.	Date Finish :	14.06.2017

Rig Type : YWE D-90R; Drill Method: Rotary Wash; Casing Type : NW; Driller : SHAH; Supervisor: REDZA

Depth m	Strata Thickness	Description of Strata	Sample No	Depth m	Rec Ratio	75	75	75	75	75	75	For <75	N Value	For <300
40														
		Very soft dark grey silty CLAY with some organic material.	P27/D25	40.500 / 40.950	100%	0	0	0	0	0	0		0	
41	42.00													
42		Medium dense light grey silty fine SAND.	P28/D26	42.000 / 42.450	100%	1	5	5	7	8	6		26	
43		Medium dense light grey silty fine SAND.	P29/D27	43.500 / 43.950	82%	0	0	4	5	7	8		24	
44														
45	45.45	Medium dense light grey silty fine SAND.	P30/D28	45.000 / 45.450	78%	1	4	6	6	4	4		20	
46		End of BH 12A at 45.45 m depth. Standpipe installed at 12.00m depth.												

(Depth scale continues 47–60.) Rec = Recovery

SPT PLOT

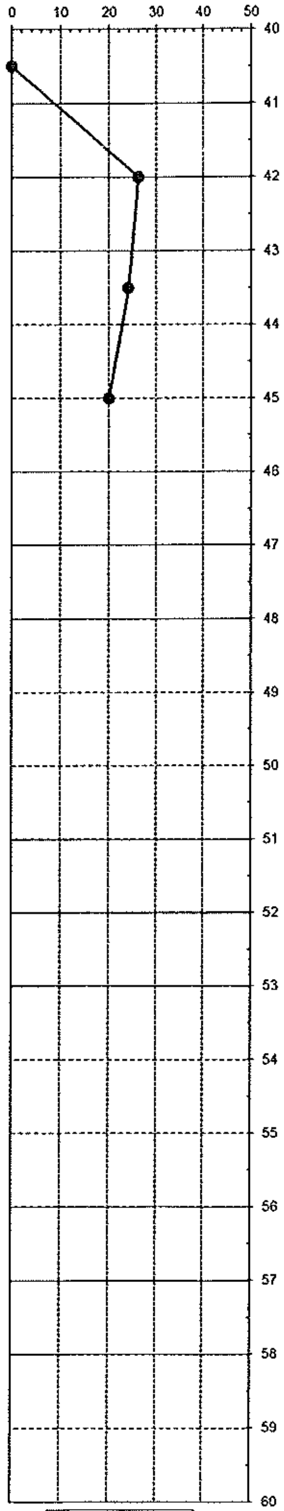

Legend:

- D ⊠ Disturbed Sample
- P Standard Penetration Test
- UD Undisturbed Sample
- MZ Mazier Sample
- VS Vane Shear Test
- C Rock Coring
- W Water Sample
- N - No. of Blows/300mm

NOTE:

** Existing ground level

Example: 50 | 120 = 50 Blows/120 mm

Cohesive Soil (N): 0, 2, 4, 8, 15, 30 — V.Soft, Soft, Firm, Stiff, V.Stiff, Hard

Non-cohesive Soil (N): 0, 4, 10, 30, 50 — V.Loose, Loose, Med Dense, Dense, V.Dense

HSD ENGINEERING SERVICES

ENGINEERING BOREHOLE LOG

Project : SOIL INVESTIGATION WORKS			Borehole No : BH 13
Location : BAGAN DATUK WATER CITY PHASE 1, PERAK DARUL RIDZUAN			Ground Level: 0.066 m
Client : PERBADANAN KEMAJUAN NEGERI PERAK	Rig Type : YWE D-90R	Driller : SHAH	Water Level : 0.50 m
Consultant: INFRA TECH GEO SOULTIONS (M) SDN. BHD.	Drill Method: Rotary Wash	Supervisor: REDZA	Date Start : 22.05.2017
Maincon : INFRA TECH PROJECTS MALAYSIA SDN. BHD.	Casing Type : NW		Date Finish : 24.05.2017

Depth m	Strata Thick-ness	Description of Strata	Log	SAMPLING DETAIL Sample No	Depth m	Rec Ratio	Penetration, P (mm) 75	75	75	75	75	75	For <75	N Value	For <300
0	0.30	Top soil.		D	0.300										
1.5		Very soft dark grey silty CLAY.		P1/D1	1.500<br>1.950	100%	0	0	0	0	0	0		0	
3		Very soft dark grey silty CLAY.		P2/D2	3.000<br>3.450	100%	0	0	0	0	0	0		0	
4.5	5.00	Very soft dark grey silty CLAY with some seashell and lenses of fine grained sand.		P3/D3	4.500<br>4.950	100%	0	0	0	0	0	0		0	
5	6.00	Dark grey sandy CLAY of intermediate plasticity.		UD 1	5.000<br>5.500	100%									
6	7.50	Very soft dark grey silty CLAY with some seashell and lenses of fine grained sand.		P4/D4	6.000<br>6.450	100%	0	0	0	0	0	0		0	
7.5	8.00	Very loose dark grey fine SAND.		P5/D5	7.500<br>7.950	58%	0	1	0	1	1	1		3	
8	9.00	Dark grey CLAY of high plasticity.		UD 2	8.000<br>8.500	100%									
9	10.50	Very loose dark grey fine SAND with lenses of clay and some organic.		P6/D6	9.000<br>9.450	89%	1	0	0	1	1	1		3	
10.5		Very soft grey CLAY with lenses of fine sand and some organic material.		P7/D7	10.500<br>10.950	100%	1	0	0	0	0	0		1	
12		Very soft dark grey CLAY with organic material.		P8/D8	12.000<br>12.450	100%	0	0	0	0	0	0		0	
13.5		Very soft dark grey CLAY with some organic material.		P9/D9	13.500<br>13.950	100%	0	0	0	0	0	0		0	
14		Dark grey CLAY of high plasticity.		UD 3	14.000<br>14.500	100%									
15	16.50	Very soft dark grey CLAY with lenses of fine grained sand and some organic material.		P10/D10	15.000<br>15.450	100%	0	0	0	0	0	0		0	
16.5	18.00	Very soft dark grey sandy CLAY with some seashell.		P11/D11	16.500<br>16.950	100%	0	0	0	0	0	0		0	
18		Very soft dark grey silty CLAY with lenses of fine sand and decayed wood and seashell.		P12/D12	18.000<br>18.450	100%	0	0	0	0	1	0		1	
19.5		Very soft dark grey silty CLAY with decayed wood.		P13/D13	19.500<br>19.950	100%	0	0	0	0	0	0		0	
20						Rec = Recovery									

SPT PLOT

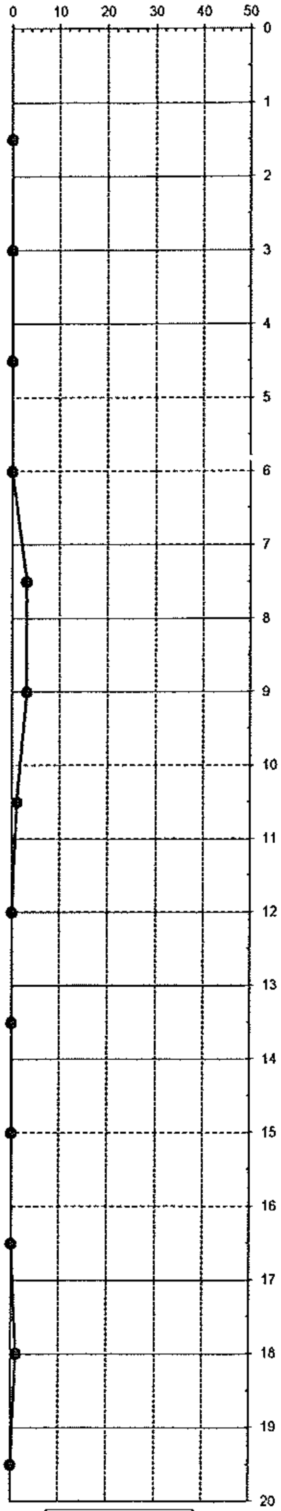

Legend:

- D — Disturbed Sample
- P — Standard Penetration Test
- UD — Undisturbed Sample
- MZ — Mazier Sample
- VS — Vane Shear Test
- C — Rock Coring
- W — Water Sample
- N - No. of Blows/300mm

NOTE:

** Existing ground level

Example:

50 | 120 = 50 Blows/120 mm

Cohesive Soil (N)	0	2	4	8	15	30	
	V.Soft	Soft	Firm	Stiff	V.Stiff	Hard	
Non-cohesive Soil (N)	0	4	10	30	50		
	V.Loose	Loose	Med Dense	Dense	V.Dense		

HSD ENGINEERING SERVICES

ENGINEERING BOREHOLE LOG

Project :	SOIL INVESTIGATION WORKS	Borehole No :	BH 13
Location :	BAGAN DATUK WATER CITY PHASE 1, PERAK DARUL RIDZUAN	Ground Level:	0.066 m
Client :	PERBADANAN KEMAJUAN NEGERI PERAK	Water Level :	0.50 m
Consultant:	INFRA TECH GEO SOULTIONS (M) SDN. BHD.	Date Start :	22.05.2017
Maincon :	INFRA TECH PROJECTS MALAYSIA SDN. BHD.	Date Finish :	24.05.2017

Rig Type : YWE D-90R | Drill Method: Rotary Wash | Casing Type : NW | Driller : SHAH | Supervisor: REDZA

Depth m	Strata Thickness	Description of Strata	Log	Sample No	Depth m	Rec Ratio	75	75	75	75	75	75	For <75	N Value	For <300
20	21.00	Dark grey silty SAND.		UD 4	20.000 / 20.500	100%									
21		Very soft dark grey silty CLAY with decayed wood and some seashell.		P14/D14	21.000 / 21.450	100%	0	0	0	0	0	0		0	
22		Very soft dark grey silty CLAY with decayed wood.		P15/D15	22.500 / 22.950	100%	0	0	0	0	0	0		0	
24		Very soft dark grey silty CLAY with decayed wood.		P16/D16	24.000 / 24.450	100%	0	0	0	0	0	0		0	
25		Very soft dark grey silty CLAY with decayed wood.		P17/D17	25.500 / 25.950	100%	0	0	0	0	0	0		0	
27		Very soft dark grey silty CLAY with decayed wood.		P18/D18	27.000 / 27.450	100%	0	0	0	0	0	0		0	
28		Soft dark grey silty CLAY with decayed wood.		P19/D19	28.500 / 28.950	100%	0	0	0	1	1	1		3	
30		Soft dark grey silty CLAY with decayed wood.		P20/D20	30.000 / 30.450	100%	0	0	0	1	1	1		3	
31		Very soft dark grey silty CLAY with decayed wood.		P21/D21	31.500 / 31.950	100%	0	0	0	0	0	0		0	
33		Firm light grey silty CLAY with decayed wood.		P22/D22	33.000 / 33.450	67%	0	1	1	2	1	2		6	
34		Very stiff greenish grey silty CLAY.		P23/D23	34.500 / 34.950	62%	2	4	9	6	6	5		26	
36	37.50	Very soft to soft greenish grey silty CLAY with decayed wood.		P24/D24	36.000 / 36.450	100%	0	1	0	1	0	1		2	
37	39.00	Very loose light grey fine to medium coarse SAND.		P25/D25	37.500 / 37.950	100%	1	0	0	1	0	1		2	
39		Soft dark grey silty CLAY with lenses of fine grained sand.		P26/D26	39.000 / 39.450	100%	0	0	0	0	1	1		2	
40															

Rec = Recovery

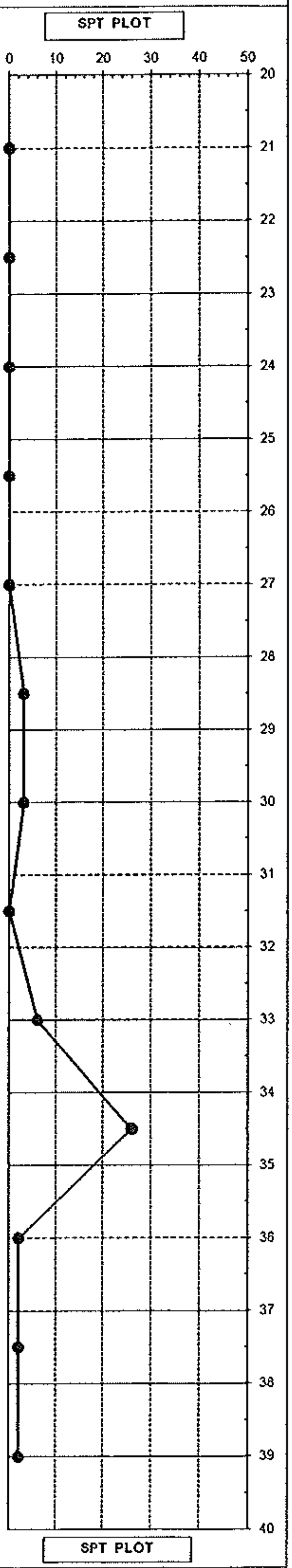

Legend:

- D — Disturbed Sample
- P — Standard Penetration Test
- UD — Undisturbed Sample
- MZ — Mazier Sample
- VS — Vane Shear Test
- C — Rock Coring
- W — Water Sample
- N - No. of Blows/300mm

NOTE:

** Existing ground level

Example:

50 | 120 = 50 Blows/120 mm

Cohesive Soil (N): 0, 2, 4, 8, 15, 30 — V.Soft, Soft, Firm, Stiff, V.Stiff, Hard

Non-cohesive Soil (N): 0, 4, 10, 30, 50 — V.Loose, Loose, Med Dense, Dense, V.Dense

HSD ENGINEERING SERVICES

ENGINEERING BOREHOLE LOG

Project :	SOIL INVESTIGATION WORKS	Borehole No :	BH 13
Location :	BAGAN DATUK WATER CITY PHASE 1, PERAK DARUL RIDZUAN	Ground Level:	0.068 m
Client :	PERBADANAN KEMAJUAN NEGERI PERAK	Water Level :	0.50 m
Consultant:	INFRA TECH GEO SOULTIONS (M) SDN. BHD.	Date Start :	22.05.2017
Maincon :	INFRA TECH PROJECTS MALAYSIA SDN. BHD.	Date Finish :	24.05.2017

Rig Type :	YWE D-90R	Driller :	SHAH
Drill Method:	Rotary Wash	Supervisor:	REDZA
Casing Type :	NW		

Depth m	Strata Thickness	Description of Strata	Sample No	Depth m	Rec Ratio	75	75	75	75	75	75	For <75	N Value	For <300
40–42	42.00	Soft to firm light grey silty CLAY with lenses of fine grained sand.	P27/D27	40.500 / 40.950	100%	0	0	0	1	2	1		4	
42–43.50	43.50	Very soft to soft darj grey sandy CLAY.	P28/D28	42.000 / 42.450	100%	0	1	0	1	0	1		2	
43.50–45		Dense grey coarse SAND.	P29/D29	43.500 / 43.950	71%	4	5	5	7	10	9		31	
45–45.45	45.45	Dense grey medium to coarse grained SAND.	P30/D30	45.000 / 45.450	42%	5	6	7	10	12	7		36	

End of BH 13 at 45.45 m depth.

Standpipe installed at 18.00m depth.

Rec = Recovery

SPT PLOT

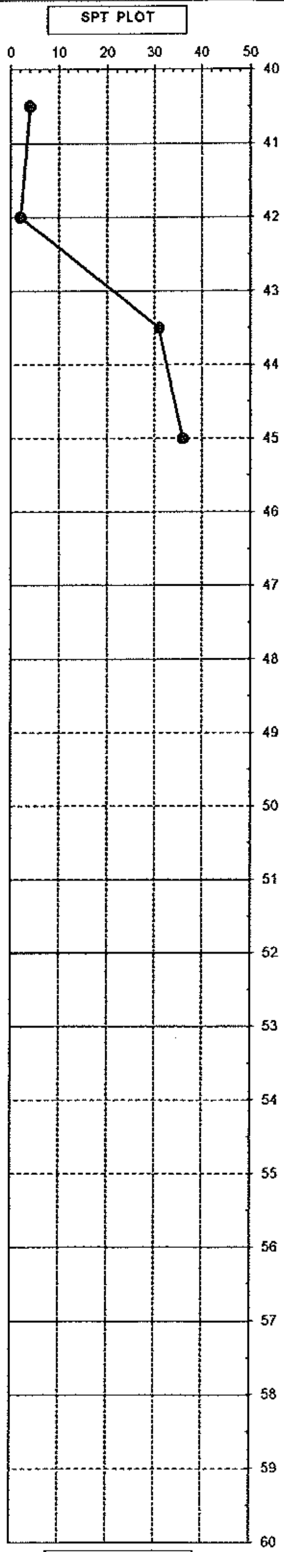

SPT PLOT

Legend:

- D — Disturbed Sample
- P — Standard Penetration Test
- UD — Undisturbed Sample
- MZ — Mazier Sample
- VS — Vane Shear Test
- C — Rock Coring
- W — Water Sample
- N - No. of Blows/300mm

NOTE:

** Existing ground level

Example:

50 | 120 = 50 Blows/120 mm

Cohesive Soil (N): 0, 2, 4, 8, 15, 30 — V.Soft, Soft, Firm, Stiff, V.Stiff, Hard

Non-cohesive Soil (N): 0, 4, 10, 30, 50 — V.Loose, Loose, Med Dense, Dense, V.Dense

HSD ENGINEERING SERVICES

ENGINEERING BOREHOLE LOG

Project :	SOIL INVESTIGATION WORKS	Borehole No :	BH 14
Location :	BAGAN DATUK WATER CITY PHASE 1, PERAK DARUL RIDZUAN	Ground Level:	0.257 m
Client :	PERBADANAN KEMAJUAN NEGERI PERAK	Water Level :	
Consultant:	INFRA TECH GEO SOULTIONS (M) SDN. BHD.	Date Start :	13.05.2017
Maincon :	INFRA TECH PROJECTS MALAYSIA SDN. BHD.	Date Finish :	17.05.2017

Rig Type : YWE D-90R | Drill Method: Rotary Wash | Casing Type : NW

Driller : SHAH | Supervisor: REDZA

Depth m	Strata Thickness	Description of Strata	Log	Sample No		Depth m	Rec Ratio	75	75	75	75	75	75	For <75	N Value	For <300
0	0.30	Top soil.			D	0.300										
1.5		Very soft grey CLAY.		P1/D1	P	1.500<br>1.950	100%	0	0	0	0	0	0		0	
3		Very soft grey CLAY.		P2/D2	P	3.000<br>3.450	100%	0	0	0	0	0	0		0	
3.5	4.50	Grey CLAY of intermediate plasticity.		UD 1	UD	3.500<br>4.000	100%									
4.5	6.00	No recovery.		P3	P	4.500<br>4.950	0%	0	0	0	0	0	0		0	
6	7.50	Very loose dark grey clayey SAND with some seashell.		P4/D3	P	6.000<br>6.450	100%	1	0	0	0	0	0		0	
7.5		Very soft grey CLAY with some seashell.		P5/D4	P	7.500<br>7.950	100%	0	0	0	0	0	0		0	
8		Grey CLAY of intermediate plasticity.		UD 2	UD	8.000<br>8.500	100%									
9		Very soft grey CLAY with some seashell.		P6/D5	P	9.000<br>9.450	100%	0	0	0	0	0	0		0	
10.5		Very soft grey CLAY with some seashell and lenses of fine sand.		P7/D6	P	10.500<br>10.950	100%	0	0	0	0	0	0		0	
12		Very soft grey CLAY.		P8/D7	P	12.000<br>12.450	100%	0	0	0	0	0	0		0	
12.5		Grey CLAY of extremely plasticity.		UD 3	UD	12.500<br>13.000	100%									
13.5		Very soft grey CLAY.		P9/D8	P	13.500<br>13.950	100%	0	0	0	0	0	0		0	
15		Very soft grey CLAY.		P10/D9	P	15.000<br>15.450	100%	0	0	0	0	0	0		0	
16.5		Very soft grey CLAY with decayed wood.		P11/D10	P	16.500<br>16.950	100%	0	0	0	0	0	0		0	
18		Soft light grey CLAY with lenses of fine sand and organic material.		P12/D11	P	18.000<br>18.450	100%	0	0	0	1	1	0		2	
18.5		Grey CLAY of extremely plasticity.		UD 4	UD	17.000<br>17.500	100%									
19.5		Very soft grey CLAY.		P13/D12	P	19.500<br>19.950	100%	0	0	0	0	0	0		0	
20																

Rec = Recovery

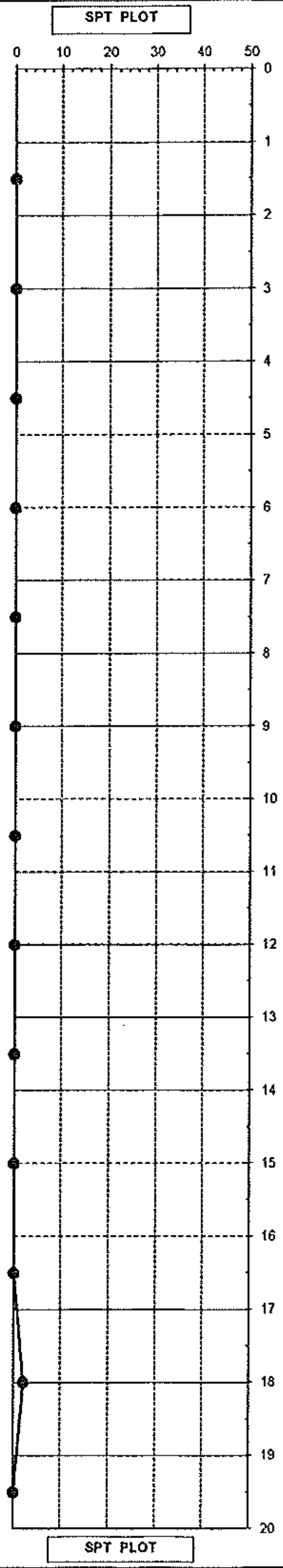

Legend:

- D — Disturbed Sample
- P — Standard Penetration Test
- UD — Undisturbed Sample
- MZ — Mazier Sample
- VS — Vane Shear Test
- C — Rock Coring
- W — Water Sample
- N - No. of Blows/300mm

NOTE:

** Existing ground level

Example:

50 | 120 = 50 Blows/120 mm

Cohesive Soil (N): 0 2 4 8 15 30 — V.Soft, Soft, Firm, Stiff, V.Stiff, Hard

Non-cohesive Soil (N): 0 4 10 30 50 — V.Loose, Loose, Med Dense, Dense, V.Dense

HSD ENGINEERING SERVICES

ENGINEERING BOREHOLE LOG

Project : SOIL INVESTIGATION WORKS			Borehole No : BH 14
Location : BAGAN DATUK WATER CITY PHASE 1, PERAK DARUL RIDZUAN			Ground Level: 0.257 m
Client : PERBADANAN KEMAJUAN NEGERI PERAK	Rig Type : YWE D-90R	Driller : SHAH	Water Level : 0.00 m
Consultant: INFRA TECH GEO SOULTIONS (M) SDN. BHD.	Drill Method: Rotary Wash	Supervisor: REDZA	Date Start : 13.05.2017
Maincon : INFRA TECH PROJECTS MALAYSIA SDN. BHD.	Casing Type : NW		Date Finish : 17.05.2017

Depth m	Strata Thickness	Description of Strata	Sample No	Depth m	Rec Ratio	75	75	75	75	75	75	For <75	N Value	For <300
20	21.00	Grey CLAY of very high plasticity.	UD 5	20.000<br>20.500	100%									
21		Very soft grey silty CLAY with decayed wood.	P14/D13	21.000<br>21.450	100%	0	0	0	0	0	0		0	
22		Very soft grey silty CLAY with decayed wood.	P15/D14	22.500<br>22.950	100%	0	0	0	0	0	0		0	
24		Very soft grey silty CLAY with decayed wood.	P16/D15	24.000<br>24.450	100%	0	0	0	0	0	0		0	
25		Very soft grey silty CLAY with decayed wood.	P17/D16	25.500<br>25.950	100%	0	0	0	0	0	0		0	
27		Very soft grey silty CLAY with decayed wood.	P18/D17	27.000<br>27.450	100%	0	0	0	0	0	0		0	
28		Very soft grey silty CLAY with decayed wood.	P19/D18	28.500<br>28.950	100%	0	0	0	0	1	0		1	
30		Very soft grey silty CLAY with decayed wood.	P20/D19	30.000<br>30.450	100%	0	0	0	0	0	0		0	
31		Very soft grey silty CLAY with decayed wood.	P21/D20	31.500<br>31.950	56%	0	0	0	0	0	0		0	
33		Stiff light grey silty CLAY with layering of fine sand and organic material.	P22/D21	33.000<br>33.450	100%	0	1	1	3	3	5		12	
34		Soft grey silty CLAY with some orgnic material.	P23/D22	34.500<br>34.950	100%	0	0	1	1	1	1		4	
36		Very soft grey silty CLAY.	P24/D23	36.000<br>36.450	100%	0	0	0	0	1	1		2	
37		Firm dark grey silty CLAY.	P25/D24	37.500<br>37.950	100%	0	0	1	1	2	1		5	
39		Firm dark grey silty CLAY.	P26/D25	39.000<br>39.450	100%	0	1	1	2	1	2		6	
40														

Rec = Recovery

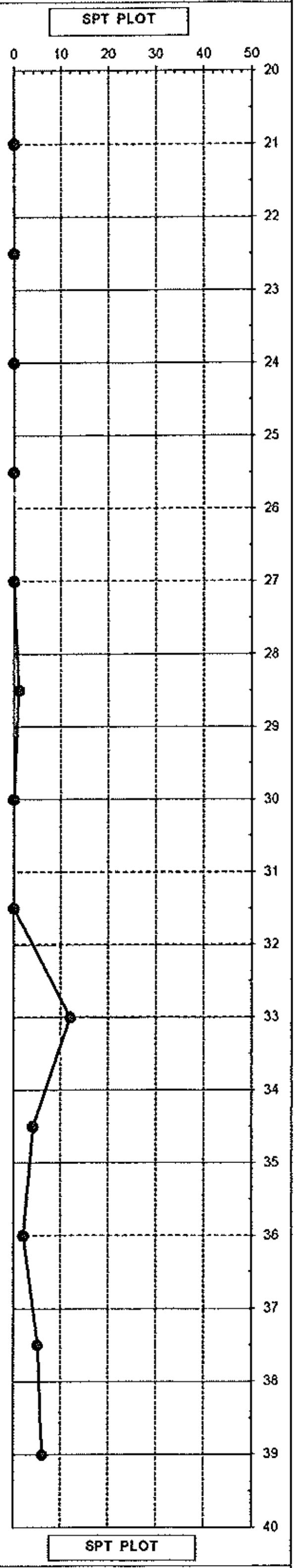

Legend:

- D Disturbed Sample
- P Standard Penetration Test
- UD Undisturbed Sample
- MZ Mazier Sample
- VS Vane Shear Test
- C Rock Coring
- W Water Sample
- N - No. of Blows/300mm

NOTE:

** Existing ground level

Example:

50 | 120 = 50 Blows/120 mm

Cohesive Soil (N)	0	2	4	8	15	30
	V.Soft,	Soft,	Firm,	Stiff,	V.Stiff,	Hard
Non-cohesive Soil (N)	0	4	10	30	50	
	V.Loose	Loose	Med Dense	Dense	V.Dense	

HSD ENGINEERING SERVICES

ENGINEERING BOREHOLE LOG

Project : SOIL INVESTIGATION WORKS			Borehole No : BH 14
Location : BAGAN DATUK WATER CITY PHASE 1, PERAK DARUL RIDZUAN			Ground Level: 0.257 m
Client : PERBADANAN KEMAJUAN NEGERI PERAK	Rig Type : YWE D-90R	Driller : SHAH	Water Level : 0.00 m
Consultant: INFRA TECH GEO SOULTIONS (M) SDN. BHD.	Drill Method: Rotary Wash	Supervisor: REDZA	Date Start : 13.05.2017
Maincon : INFRA TECH PROJECTS MALAYSIA SDN. BHD.	Casing Type : NW		Date Finish : 17.05.2017

Depth m	Strata Thickness	Description of Strata	Log	Sample No	Depth m	Rec Ratio	75	75	75	75	75	75	For <75	N Value	For <300
40 – 42	42.00	Firm dark grey silty CLAY with some decayed wood.		P27/D26	40.500 40.950	100%	0	1	1	1	2	2		6	
42 – 43.5	43.50	Very stiff light grey clayey fine SAND.		P28/D27	42.000 42.450	100%	0	1	3	5	6	6		20	
43.5 – 45	45.00	No recovery.		P29	43.500 43.950	0%	2	3	4	5	6	8		23	
45 – 45.45	45.45	Dense light grey clayey fine to coarse grained SAND.		P30/D28	45.000 45.450	100%	5	7	6	8	10	9		33	
		End of BH 14 at 45.45m depth. Standpipe installed at 18.00 m depth.													

(Columns 75 × 6 = Penetration, P (mm) / SPT BLOW COUNT; Rec = Recovery)

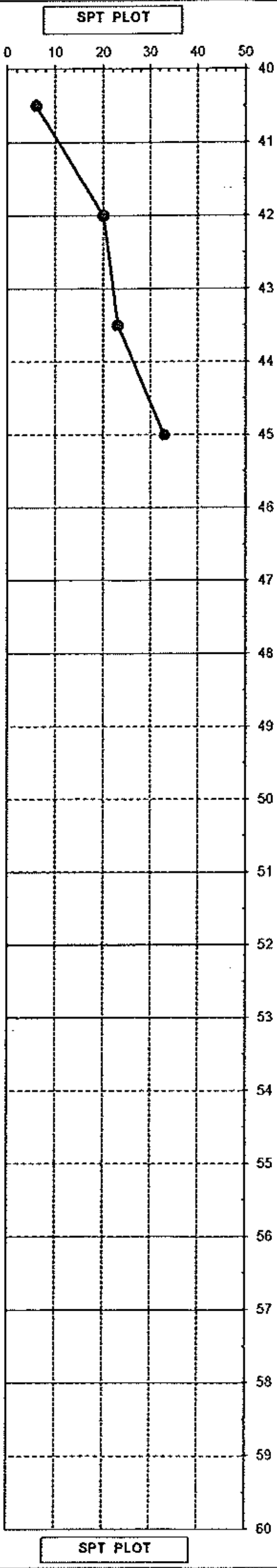

Legend:

- D – Disturbed Sample
- P – Standard Penetration Test
- UD – Undisturbed Sample
- MZ – Mazier Sample
- VS – Vane Shear Test
- C – Rock Coring
- W – Water Sample
- N - No. of Blows/300mm

NOTE:

** Existing ground level

Example:

50 | 120 = 50 Blows/120 mm

Cohesive Soil (N): 0 V.Soft, 2 Soft, 4 Firm, 8 Stiff, 15 V.Stiff, 30 Hard

Non-cohesive Soil (N): 0 V.Loose, 4 Loose, 10 Med Dense, 30 Dense, 50 V.Dense

HSD ENGINEERING SERVICES

ENGINEERING BOREHOLE LOG

Project : SOIL INVESTIGATION WORKS			Borehole No : BH 15
Location : BAGAN DATUK WATER CITY PHASE 1, PERAK DARUL RIDZUAN			Ground Level: 0.162 m
Client : PERBADANAN KEMAJUAN NEGERI PERAK	Rig Type : YWE D-90R	Driller : SHAH	Water Level :
Consultant: INFRA TECH GEO SOULTIONS (M) SDN. BHD.	Drill Method: Rotary Wash	Supervisor: REDZA	Date Start : 18.05.2017
Maincon : INFRA TECH PROJECTS MALAYSIA SDN. BHD.	Casing Type : NW		Date Finish : 22.05.2017

Depth m	Strata Thickness	Description of Strata	Sample No	Sample Type	Depth m	Rec Ratio	75	75	75	75	75	75	For <75	N Value	For <300
0	0.30	Top soil.		D	0.300										
1.5		Very soft dark grey CLAY.	P1/D1	P	1.500 / 1.950	100%	0	0	0	0	0	0		0	
3.0		Very soft dark grey CLAY.	P2/D2	P	3.000 / 3.450	100%	0	0	0	0	0	0		0	
3.5		Dark grey CLAY of intermediate plasticity.	UD 1	UD	3.500 / 4.000	74%									
4.5		Very soft dark grey CLAY.	P3/D3	P	4.500 / 4.950	100%	0	0	0	0	0	0		0	
6.0		Very soft dark grey CLAY.	P4/D4	P	6.000 / 6.450	100%	0	0	0	0	0	0		0	
7.5	8.00	Very soft dark grey CLAY of extremely high plasticity.	P5/D5	P	7.500 / 7.950	100%	0	0	0	0	0	0		0	
8.0	9.00	No recovery.	UD 2	UD	8.000 / 8.500	0%									
9.0		Very soft dark grey CLAY with lenses of fine sand.	P6/D6	P	9.000 / 9.450	100%	0	0	0	0	0	0		0	
10.5		Very soft greu CLAY with some organic seashell.	P7/D7	P	10.500 / 10.950	100%	0	0	0	0	0	0		0	
11.0		No recovery.	UD 3	UD	11.000 / 11.500	0%									
12		Casing falls from 11.00 m until 12.40 m due to very soft layer.													
13.5		Very soft grey CLAY with some seashell.	P8/D8	P	13.500 / 13.950	100%	0	0	0	0	0	0		0	
14.0		Grey CLAY of very high plasticity.	UD 4	UD	14.000 / 14.500	100%									
15.0		Very soft dark grey CLAY with some seashell and lenses of fine grained sand.	P9/D9	P	15.000 / 15.450	100%	0	0	0	0	0	0		0	
16.5	17.00	Very soft dark grey CLAY with some seashell.	P10/D10	P	16.500 / 16.950	100%	0	0	1	0	0	0		1	
17.0	18.00	Dark grey sandy CLAY of intermediate plasticity.	UD 5	UD	17.000 / 17.500	100%									
18.0		Very soft grey CLAY with lenses of fine sand and decayed wood.	P11/D11	P	18.000 / 18.450	100%	0	1	0	0	1	0		1	
19.5		Very soft grey CLAY with some seashell.	P12/D12	P	19.500 / 19.950	100%	1	0	0	1	0	1		2	
20															

Rec = Recovery

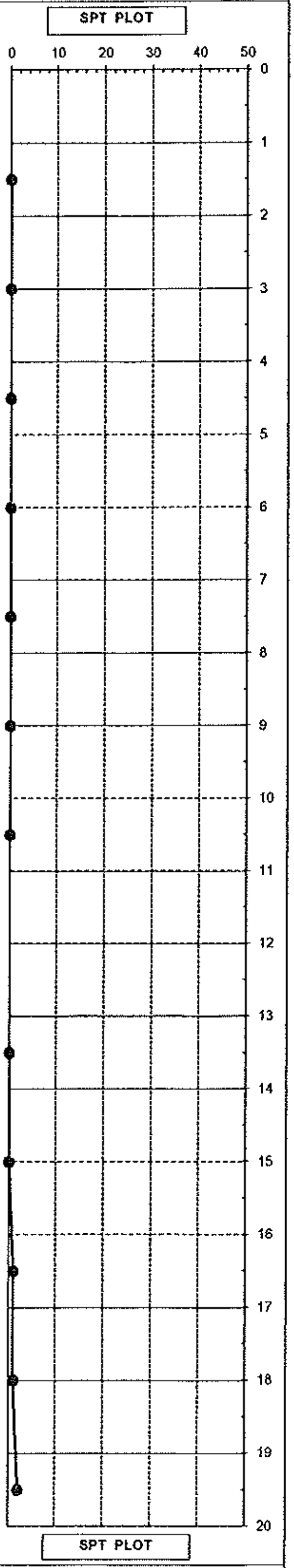

Legend:

- D – Disturbed Sample
- P – Standard Penetration Test
- UD – Undisturbed Sample
- MZ – Mazier Sample
- VS – Vane Shear Test
- C – Rock Coring
- W – Water Sample
- N – No. of Blows/300mm

NOTE:

** Existing ground level

Example:

50 | 120 = 50 Blows/120 mm

Cohesive Soil (N)	0	2	4	8	15	30	
	V.Soft,	Soft,	Firm,	Stiff,	V.Stiff,	Hard	
Non-cohesive Soil (N)	0	4	10	30	50		
	V.Loose	Loose	Med Dense	Dense	V.Dense		

HSD ENGINEERING SERVICES

ENGINEERING BOREHOLE LOG

Project : SOIL INVESTIGATION WORKS	Borehole No : BH 15
Location : BAGAN DATUK WATER CITY PHASE 1, PERAK DARUL RIDZUAN	Ground Level: 0.162 m
Client : PERBADANAN KEMAJUAN NEGERI PERAK / Rig Type : YWE D-90R / Driller : SHAH	Water Level : 0.00 m
Consultant: INFRA TECH GEO SOULTIONS (M) SDN. BHD. / Drill Method: Rotary Wash / Supervisor: REDZA	Date Start : 18.05.2017
Maincon : INFRA TECH PROJECTS MALAYSIA SDN. BHD. / Casing Type : NW	Date Finish : 22.05.2017

Depth m	Strata Thickness	Description of Strata	Log	Sample No	Depth m	Rec Ratio	75	75	75	75	75	75	For <75	N Value	For <300
20															
21		Very soft grey silty CLAY with some seashell.		P13/D13	21.000 21.450	89%	1	0	0	0	0	0		1	
22		Very soft grey silty CLAY with some decayed wood.		P14/D14	22.500 22.950	100%	0	0	0	0	0	0		0	
24		Firm grey silty CLAY with some decayed wood.		P15/D15	24.000 24.450	100%	1	0	0	1	2	2		5	
25		Very soft to soft grey silty CLAY with some decayed wood.		P16/D16	25.500 25.950	100%	1	0	1	0	0	1		2	
27		Very soft to soft grey silty CLAY with some decayed wood.		P17/D17	27.000 27.450	100%	0	0	0	0	0	0		0	
28		Very soft to soft grey silty CLAY with some decayed wood.		P18/D18	28.500 28.950	100%	0	0	0	0	0	0		0	
30		Very soft to soft grey silty CLAY with some decayed wood.		P19/D19	30.000 30.450	100%	0	0	0	0	1	1		2	
31	33.00	Very soft brownish grey silty CLAY with some organic material.		P20/D20	31.500 31.950	100%	0	0	0	0	0	0		0	
33	34.50	Very soft light grey sandy CLAY of intermediate plasticity.		P21/D21	33.000 33.450	100%	0	0	0	0	0	0		0	
34		Very soft dark grey silty CLAY.		P22/D22	34.500 34.950	84%	0	0	1	1	1	1		4	
36		Very soft light grey silty CLAY.		P23/D23	36.000 36.450	100%	0	0	0	0	0	0		0	
37		Very soft light grey silty CLAY interbedded with fine to medium coarse graned sand.		P24/D24	37.500 37.950	100%	0	0	0	0	0	0		0	
39		Very soft to soft light grey silty CLAY.		P25/D25	39.000 39.450	100%	0	0	0	1	0	1		2	
40															

Rec = Recovery

SPT PLOT

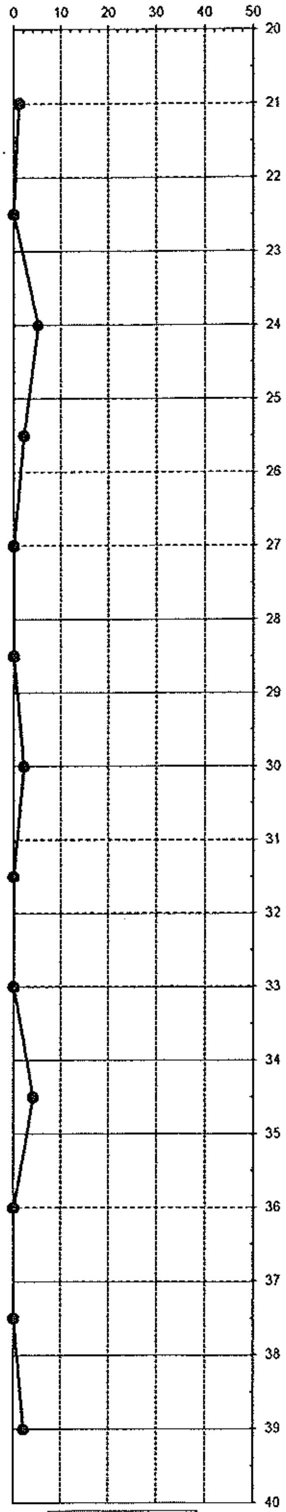

SPT PLOT

Legend:

- D — Disturbed Sample
- P — Standard Penetration Test
- UD — Undisturbed Sample
- MZ — Mazier Sample
- VS — Vane Shear Test
- C — Rock Coring
- W — Water Sample
- N - No. of Blows/300mm

NOTE:

** Existing ground level

Example:

50 | 120 = 50 Blows/120 mm

Cohesive Soil (N): 0 2 4 8 15 30 — V.Soft, Soft, Firm, Stiff, V.Stiff, Hard

Non-cohesive Soil (N): 0 4 10 30 50 — V.Loose, Loose, Med Dense, Dense, V.Dense

HSD ENGINEERING SERVICES

ENGINEERING BOREHOLE LOG

Project : SOIL INVESTIGATION WORKS			Borehole No : BH 15
Location : BAGAN DATUK WATER CITY PHASE 1, PERAK DARUL RIDZUAN			Ground Level: 0.162 m
Client : PERBADANAN KEMAJUAN NEGERI PERAK	Rig Type : YWE D-90R	Driller : SHAH	Water Level : 0.00 m
Consultant: INFRA TECH GEO SOULTIONS (M) SDN. BHD.	Drill Method: Rotary Wash	Supervisor: REDZA	Date Start : 18.05.2017
Maincon : INFRA TECH PROJECTS MALAYSIA SDN. BHD.	Casing Type : NW		Date Finish : 22.05.2017

Depth m	Strata Thick-ness	Description of Strata	Log	Sample No	Depth m	Rec Ratio	75	75	75	75	75	75	For <75	N Value	For <300
40															
40.5		Soft dark grey silty CLAY.	x	P26/D26	40.500 40.950	100%	0	0	1	1	1	1		4	
42		Soft dark grey silty CLAY.	x	P27/D27	42.000 42.450	100%	0	0	1	1	1	1		4	
43.5	43.50	Medium dense light grey silty medium to coarse SAND with some gravels.	::::	P28/D28	43.500 43.950	100%	0	1	1	2	3	4		10	
45	45.45	Very dense light grey silty medium to coarse SAND and some gravels.	::::	P29/D29	45.000 45.400	53%	2	9	12	17	15	6	25	50	250
46		End of BH 15 at 45.45 m depth.													
60															

SPT PLOT

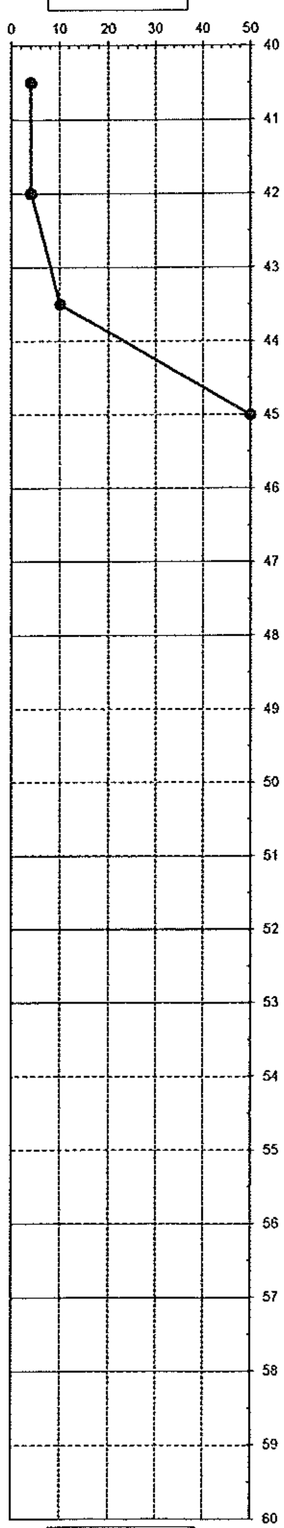

SPT PLOT

Rec = Recovery

Legend:

D – Disturbed Sample
P – Standard Penetration Test
UD – Undisturbed Sample
MZ – Mazier Sample
VS – Vane Shear Test
C – Rock Coring
W – Water Sample
N - No. of Blows/300mm

NOTE:

** Existing ground level

Example:

50 | 120 = 50 Blows/120 mm

Cohesive Soil (N): 0 2 4 8 15 30 — V.Soft, Soft, Firm, Stiff, V.Stiff, Hard

Non-cohesive Soil (N): 0 4 10 30 50 — V.Loose, Loose, Med Dense, Dense, V.Dense

HSD ENGINEERING SERVICES

ENGINEERING BOREHOLE LOG

Project :	SOIL INVESTIGATION WORKS	Borehole No :	BH 16
Location :	BAGAN DATUK WATER CITY PHASE 1, PERAK DARUL RIDZUAN	Ground Level:	0.060 m
Client :	PERBADANAN KEMAJUAN NEGERI PERAK	Rig Type : YWE D-90R	Driller : SHAH
Consultant:	INFRA TECH GEO SOULTIONS (M) SDN. BHD.	Drill Method: Rotary Wash	Supervisor: REDZA
Maincon :	INFRA TECH PROJECTS MALAYSIA SDN. BHD.	Casing Type : NW	
Water Level :	1.20 m	Date Start :	23.05.2017
Date Finish :	03.06.2017		

Depth m	Strata Thick-ness	Description of Strata	Log	Sample No	Depth m	Rec Ratio	75	75	75	75	75	75	For <75	N Value	For <300
0	0.30	Top soil.		D1	0.300										
		Very soft dark grey CLAY.		P1/D2	1.500 1.950	100%	0	1	0	0	0	0		0	
	3.50	Very soft dark grey CLAY.		P2/D3	3.000 3.450	100%	0	0	0	0	0	0		0	
	4.50	Dark grey silty SAND.		UD 1	3.500 4.000	100%									
		Very soft dark grey CLAY.		P3/D4	4.500 4.950	100%	1	0	0	0	0	0		0	
		Very soft to soft dark grey CLAY.		P4/D5	6.000 6.450	100%	1	0	1	0	0	1		2	
	8.00	Very soft dark grey CLAY.		P5/D6	7.500 7.950	100%	1	0	0	0	0	1		1	
	9.00	Dark grey silty SAND.		UD 2	8.000 8.500	100%									
		Very soft to soft dark grey CLAY.		P6/D7	9.000 9.450	100%	1	0	0	1	0	1		2	
		Very soft dark grey CLAY.		P7/D8	10.500 10.950	100%	0	0	0	1	0	0		1	
		Very soft dark grey CLAY.		P8/D9	12.000 12.450	100%	0	0	0	0	0	0		0	
		Very soft dark grey CLAY.		P9/D10	13.500 13.950	100%	0	0	0	0	0	0		0	
		Dark grey CLAY of extremely high plasticity.		UD 3	14.000 14.500	100%									
		Very soft dark grey CLAY.		P10/D11	15.000 15.450	100%	1	1	0	1	0	0		1	
		Soft dark grey CLAY.		P11/D12	16.500 16.950	100%	1	1	0	1	1	1		3	
		Firm dark grey CLAY.		P12/D13	18.000 18.450	100%	1	0	2	1	1	1		5	
		Dark grey CLAY of extremely high plasticity.		UD 4	18.500 19.000	100%									
20		Soft dark grey CLAY.		P13/D14	19.500 19.950	100%	0	0	1	1	0	1		3	

Rec = Recovery

SPT PLOT

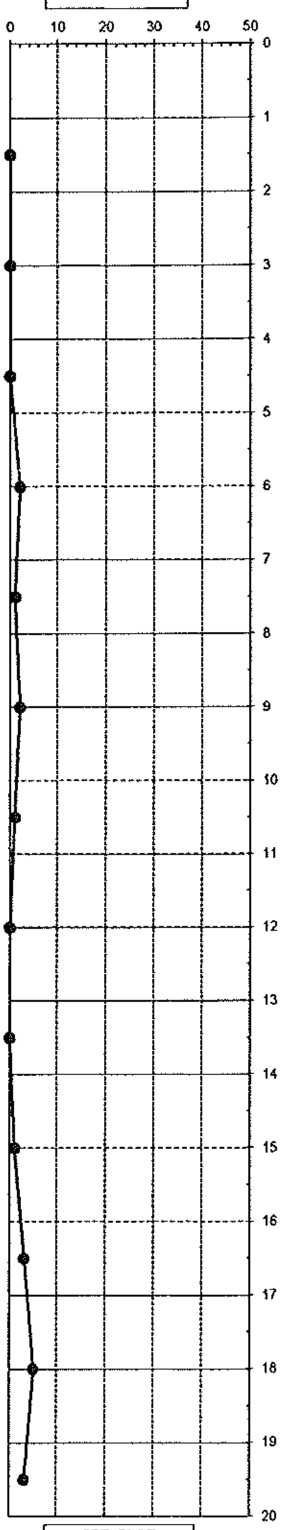

SPT PLOT

Legend:

- D — Disturbed Sample
- P — Standard Penetration Test
- UD — Undisturbed Sample
- MZ — Mazier Sample
- VS — Vane Shear Test
- C — Rock Coring
- W — Water Sample
- N - No. of Blows/300mm

NOTE:

** Existing ground level

Example:

50 | 120 = 50 Blows/120 mm

Cohesive Soil (N): 0, 2, 4, 8, 15, 30 — V.Soft, Soft, Firm, Stiff, V.Stiff, Hard

Non-cohesive Soil (N): 0, 4, 10, 30, 50 — V.Loose, Loose, Med Dense, Dense, V.Dense

HSD ENGINEERING SERVICES

ENGINEERING BOREHOLE LOG

Project : SOIL INVESTIGATION WORKS			Borehole No : BH 16
Location : BAGAN DATUK WATER CITY PHASE 1, PERAK DARUL RIDZUAN			Ground Level: 0.060 m
Client : PERBADANAN KEMAJUAN NEGERI PERAK	Rig Type : YWE D-90R	Driller : SHAH	Water Level : 1.20 m
Consultant: INFRA TECH GEO SOULTIONS (M) SDN. BHD.	Drill Method: Rotary Wash	Supervisor: REDZA	Date Start : 23.05.2017
Maincon : INFRA TECH PROJECTS MALAYSIA SDN. BHD.	Casing Type : NW		Date Finish : 03.06.2017

Depth m	Strata Thick-ness	Description of Strata	Log	SAMPLING DETAIL Sample No	Depth m	Rec Ratio	Penetration, P (mm) 75	75	75	75	75	75	For <75	N Value	For <300
20															
21		Soft dark grey CLAY.		P14/D15	21.000 21.450	100%	1	1	0	1	1	1		3	
22		Soft dark grey CLAY.		P15/D16	22.500 22.950	100%	1	1	0	1	1	1		3	
23															
24		Soft dark grey CLAY.		P16/D17	24.000 24.450	100%	1	0	1	1	1	0		3	
25		Soft dark grey CLAY.		P17/D18	25.500 25.950	100%	1	0	0	1	1	1		3	
26															
27		Soft dark grey CLAY.		P18/D19	27.000 27.450	100%	1	0	1	0	1	1		3	
28		Soft dark grey CLAY.		P19/D20	28.500 28.950	100%	1	0	0	1	1	1		3	
29															
30		Soft dark grey CLAY.		P20/D21	30.000 30.450	100%	1	0	1	0	1	1		3	
31		Very soft dark grey CLAY.		P21/D22	31.500 31.950	100%	1	0	0	1	0	0		1	
32															
33		Very soft to soft dark grey CLAY with traces of decayed wood.		P22/D23	33.000 33.450	100%	1	1	0	1	0	1		2	
34		Soft to firm dark grey CLAY.		P23/D24	34.500 34.950	100%	1	1	1	1	1	1		4	
35	36.00														
36		Firm dark grey sandy CLAY.		P24/D25	36.000 36.450	100%	1	2	1	1	2	1		5	
37		Firm dark grey sandy CLAY.		P25/D26	37.500 37.950	100%	1	1	1	2	1	1		5	
38															
39		Soft to firm dark grey sandy CLAY.		P26/D27	39.000 39.450	100%	1	1	2	1	0	1		4	
40						Rec = Recovery									

SPT PLOT

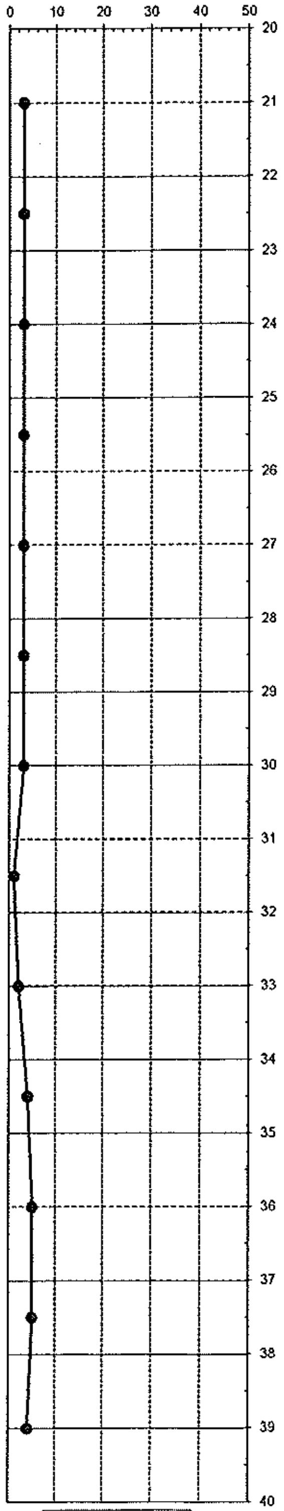

Legend:

- D — Disturbed Sample
- P — Standard Penetration Test
- UD — Undisturbed Sample
- MZ — Mazier Sample
- VS — Vane Shear Test
- C — Rock Coring
- W — Water Sample
- N - No. of Blows/300mm

NOTE:

** Existing ground level

Example:

50 | 120 = 50 Blows/120 mm

Cohesive Soil (N)	0	2	4	8	15	30
	V.Soft,	Soft,	Firm,	Stiff,	V.Stiff,	Hard

Non-cohesive Soil (N)	0	4	10	30	50
	V.Loose	Loose	Med Dense	Dense	V.Dense

HSD ENGINEERING SERVICES

ENGINEERING BOREHOLE LOG

Project :	SOIL INVESTIGATION WORKS		Borehole No : BH 16
Location :	BAGAN DATUK WATER CITY PHASE 1, PERAK DARUL RIDZUAN		Ground Level: 0.060 m
Client :	PERBADANAN KEMAJUAN NEGERI PERAK	Rig Type : YWE D-90R / Driller : SHAH	Water Level : 1.20 m
Consultant:	INFRA TECH GEO SOULTIONS (M) SDN. BHD.	Drill Method: Rotary Wash / Supervisor: REDZA	Date Start : 23.05.2017
Maincon :	INFRA TECH PROJECTS MALAYSIA SDN. BHD.	Casing Type : NW	Date Finish : 03.06.2017

Depth m	Strata Thickness	Description of Strata	Log	Sample No	Depth m	Rec Ratio	75	75	75	75	75	75	For <75	N Value	For <300
40															
40.5		Soft to firm dark grey sandy CLAY.		P27/D28	40.500 / 40.950	100%	1	2	1	1	1	1		4	
42		Soft to firm dark grey sandy CLAY.		P28/D29	42.000 / 42.450	100%	1	1	0	1	2	1		4	
43.5	43.50	Soft to firm dark grey CLAY with traces of sand.		P29/D30	43.500 / 43.950	100%	1	2	1	1	1	1		4	
45	45.45	Soft to firm dark grey CLAY with traces of sand.		P30/D31	45.000 / 45.450	100%	1	1	2	0	1	1		4	
46		End of BH 16 at 45.45 m depth.													
		Standpipe installed at 18.00 m depth.													

Penetration, P (mm) / SPT BLOW COUNT; Rec = Recovery

SPT PLOT

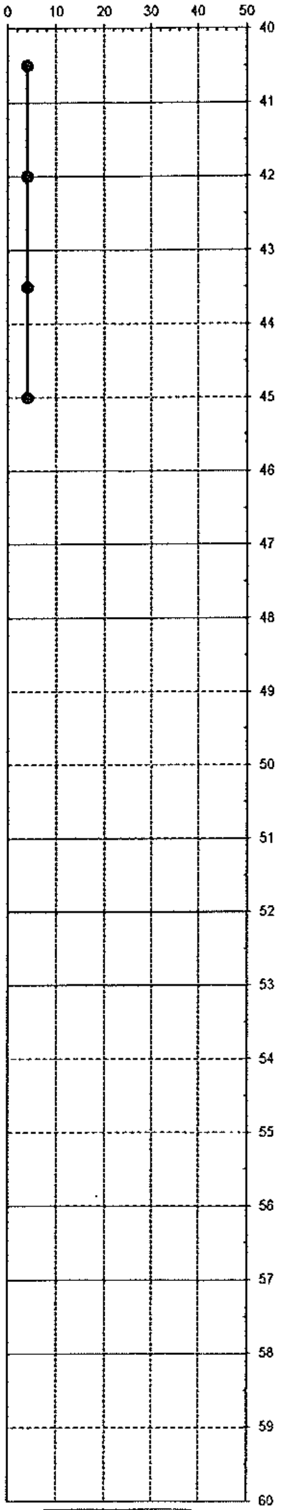

SPT PLOT

Legend:

- D – Disturbed Sample
- P – Standard Penetration Test
- UD – Undisturbed Sample
- MZ – Mazier Sample
- VS – Vane Shear Test
- C – Rock Coring
- W – Water Sample
- N - No. of Blows/300mm

NOTE:

** Existing ground level

Example:

50 | 120 = 50 Blows/120 mm

Cohesive Soil (N): 0, 2, 4, 8, 15, 30 — V.Soft, Soft, Firm, Stiff, V.Stiff, Hard

Non-cohesive Soil (N): 0, 4, 10, 30, 50 — V.Loose, Loose, Med Dense, Dense, V.Dense

HSD ENGINEERING SERVICES

ENGINEERING BOREHOLE LOG

Project :	SOIL INVESTIGATION WORKS	Borehole No :	BH 17
Location :	BAGAN DATUK WATER CITY PHASE 1, PERAK DARUL RIDZUAN	Ground Level:	0.151 m
Client :	PERBADANAN KEMAJUAN NEGERI PERAK	Rig Type : YWE D-90R	Driller : SHAH
Consultant:	INFRA TECH GEO SOULTIONS (M) SDN. BHD.	Drill Method: Rotary Wash	Supervisor: REDZA
Maincon :	INFRA TECH PROJECTS MALAYSIA SDN. BHD.	Casing Type : NW	
Water Level :	1.50 m	Date Start :	03.06.2017
Date Finish :	09.06.2017		

Depth m	Strata Thickness	Description of Strata	Log	Sample No	Depth m	Rec Ratio	75	75	75	75	75	75	For <75	N Value	For <300
0															
	0.30	Top soil.		D1	0.300										
1															
		Very soft dark grey CLAY.		P1/D2	1.500<br>1.950	100%	0	0	0	0	0	0		0	
2															
3		Very soft dark grey CLAY.		P2/D3	3.000<br>3.450	100%	0	0	0	0	0	0		0	
		Dark grey CLAY of extremely high plasticity.		UD 1	3.500<br>4.000	100%									
4															
		Very soft dark grey CLAY.		P3/D4	4.500<br>4.950	100%	1	0	1	0	0	0		1	
5															
6		Very soft dark grey CLAY.		P4/D5	6.000<br>6.450	100%	1	1	0	1	0	0		1	
		Dark grey CLAY of very high plasticity.		UD 2	6.500<br>7.000	100%									
7															
		Very soft dark grey CLAY.		P5/D6	7.500<br>7.950	100%	1	1	0	0	0	0		0	
8															
9		Very soft dark grey CLAY.		P6/D7	9.000<br>9.450	100%	1	0	0	0	0	0		0	
10															
		Soft dark grey CLAY.		P7/D8	10.500<br>10.950	100%	0	1	1	0	1	1		3	
11		Dark grey CLAY of extremely high plasticity.		UD 3	11.000<br>11.500	100%									
12		Veru soft dark grey CLAY.		P8/D9	12.000<br>12.450	100%	0	0	0	0	1	0		1	
13															
		Very soft dark grey CLAY.		P9/D10	13.500<br>13.950	100%	1	0	0	0	0	1		1	
14															
15	15.50	Very soft to soft dark grey CLAY.		P10/D11	15.000<br>15.450	100%	1	0	1	1	0	0		2	
16	16.50	Dark grey sandy CLAY of intermediate plasticity.		UD 4	15.500<br>16.000	100%									
		Very soft to soft dark grey CLAY.		P11/D12	16.500<br>16.950	100%	1	0	0	0	1	1		2	
17															
18		Very soft to soft dark grey CLAY.		P12/D13	18.000<br>18.450	100%	1	1	0	1	1	0		2	
19															
		Very soft to soft dark grey CLAY.		P13/D14	19.500<br>19.950	100%	1	0	0	1	0	1		2	
20															

Sampling Detail; Penetration, P (mm); SPT Blow Count; Rec = Recovery

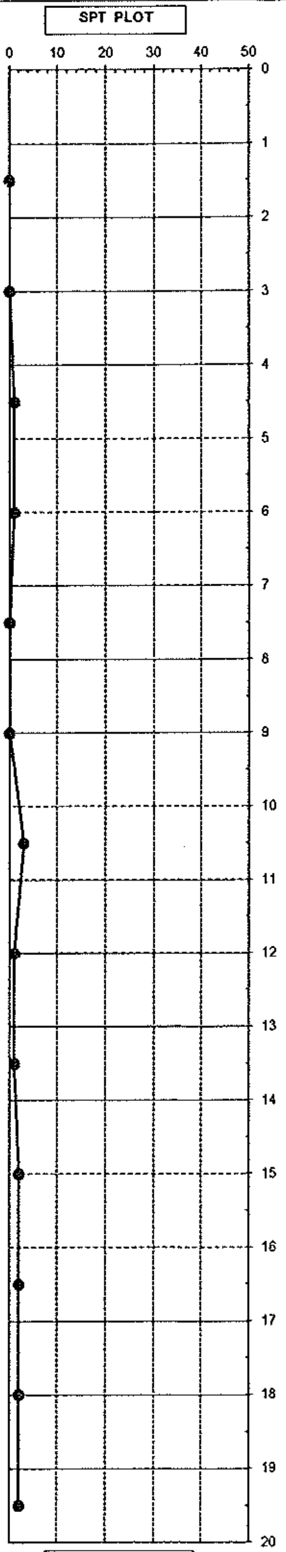

SPT PLOT

Legend:

- D — Disturbed Sample
- P — Standard Penetration Test
- UD — Undisturbed Sample
- MZ — Mazier Sample
- VS — Vane Shear Test
- C — Rock Coring
- W — Water Sample
- N - No. of Blows/300mm

NOTE:

** Existing ground level

Example: 50 | 120 = 50 Blows/120 mm

Cohesive Soil (N): 0, 2, 4, 8, 15, 30 — V.Soft, Soft, Firm, Stiff, V.Stiff, Hard

Non-cohesive Soil (N): 0, 4, 10, 30, 50 — V.Loose, Loose, Med Dense, Dense, V.Dense

HSD ENGINEERING SERVICES

ENGINEERING BOREHOLE LOG

Project :	SOIL INVESTIGATION WORKS		Borehole No : BH 17
Location :	BAGAN DATUK WATER CITY PHASE 1, PERAK DARUL RIDZUAN		Ground Level: 0.151 m
Client :	PERBADANAN KEMAJUAN NEGERI PERAK	Rig Type : YWE D-90R / Driller : SHAH	Water Level : 1.50 m
Consultant:	INFRA TECH GEO SOULTIONS (M) SDN. BHD.	Drill Method: Rotary Wash / Supervisor: REDZA	Date Start : 03.06.2017
Maincon :	INFRA TECH PROJECTS MALAYSIA SDN. BHD.	Casing Type : NW	Date Finish : 09.06.2017

Depth m	Strata Thick-ness	Description of Strata	Log	Sample No	Depth m	Rec Ratio	75	75	75	75	75	75	For <75	N Value	For <300
20		Dark grey CLAY of very high plasticity.		UD 5	20.000 20.500	100%									
21		Very soft to soft dark grey CLAY.		P14/D15	21.000 21.450	100%	1	0	0	0	1	1		2	
22		Soft dark grey CLAY.		P15/D16	22.500 22.950	100%	0	0	1	1	0	1		3	
24		Very soft to soft dark grey CLAY.		P16/D17	24.000 24.450	100%	1	0	0	1	1	0		2	
25		Very soft to soft dark grey CLAY.		P17/D18	25.500 25.950	100%	1	1	1	0	1	0		2	
27		Soft to firm dark grey CLAY.		P18/D19	27.000 27.450	100%	1	1	1	1	1	1		4	
28		Soft dark grey CLAY.		P19/D20	28.500 28.950	100%	1	1	1	1	0	1		3	
30		Soft to firm dark grey CLAY.		P20/D21	30.000 30.450	100%	1	0	1	1	1	1		4	
31		Very soft to soft dark grey CLAY.		P21/D22	31.500 31.950	100%	2	1	1	0	0	1		2	
33	33.00	Soft to firm dark grey sandy CLAY.		P22/D23	33.000 33.450	100%	1	1	2	0	1	1		4	
34		Very soft to soft dark grey sandy CLAY.		P23/D24	34.500 34.950	100%	1	1	1	1	0	0		2	
36		Firm dark grey sandy CLAY.		P24/D25	36.000 36.450	100%	2	2	2	1	1	1		5	
37		Very soft to soft dark grey sandy CLAY.		P25/D26	37.500 37.950	100%	1	1	0	1	0	1		2	
39		Firm dark grey sandy CLAY.		P26/D27	39.000 39.450	100%	2	1	2	1	1	1		5	
40															

SAMPLING DETAIL; Penetration, P (mm); SPT BLOW COUNT; Rec = Recovery

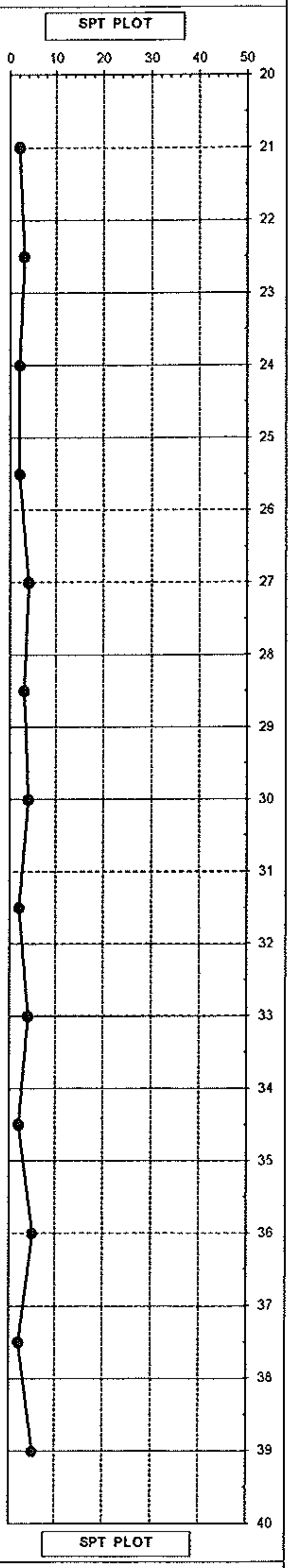

Legend:

- D – Disturbed Sample
- P – Standard Penetration Test
- UD – Undisturbed Sample
- MZ – Mazier Sample
- VS – Vane Shear Test
- C – Rock Coring
- W – Water Sample
- N - No. of Blows/300mm

NOTE:

** Existing ground level

Example:

50 | 120 = 50 Blows/120 mm

Cohesive Soil (N): 0, 2, 4, 8, 15, 30 — V.Soft, Soft, Firm, Stiff, V.Stiff, Hard

Non-cohesive Soil (N): 0, 4, 10, 30, 50 — V.Loose, Loose, Med Dense, Dense, V.Dense

HSD ENGINEERING SERVICES

ENGINEERING BOREHOLE LOG

Project : SOIL INVESTIGATION WORKS			Borehole No : BH 17
Location : BAGAN DATUK WATER CITY PHASE 1, PERAK DARUL RIDZUAN			Ground Level: 0.151 m
Client : PERBADANAN KEMAJUAN NEGERI PERAK	Rig Type : YWE D-90R	Driller : SHAH	Water Level : 1.50 m
Consultant: INFRA TECH GEO SOULTIONS (M) SDN. BHD.	Drill Method: Rotary Wash	Supervisor: REDZA	Date Start : 03.06.2017
Maincon : INFRA TECH PROJECTS MALAYSIA SDN. BHD.	Casing Type : NW		Date Finish : 09.06.2017

Depth m	Strata Thickness	Description of Strata	Log	Sample No	Depth m	Rec Ratio	75	75	75	75	75	75	For <75	N Value	For <300
40															
41		Soft to firm dark grey CLAY.		P27/D28	40.500 / 40.950	100%	1	1	1	1	1	1		4	
42		Stiff dark grey CLAY with traces of fine sand.		P28/D29	42.000 / 42.450	100%	3	2	2	4	4	2		12	
43	43.50														
44		Very stiff dark grey sandy CLAY.		P29/D30	43.500 / 43.950	100%	4	3	3	4	5	4		16	
45	45.45	Stiff dark grey sandy CLAY.		P30/D31	45.000 / 45.450	100%	4	3	3	1	2	5		11	
46		End of BH 17 at 45.45 m depth. Standpipe installed at 18.00 m depth.													
47															
48															
49															
50															
51															
52															
53															
54															
55															
56															
57															
58															
59															
60															

(Column groups: SAMPLING DETAIL; Penetration, P (mm) — SPT BLOW COUNT; N)

Rec = Recovery

SPT PLOT

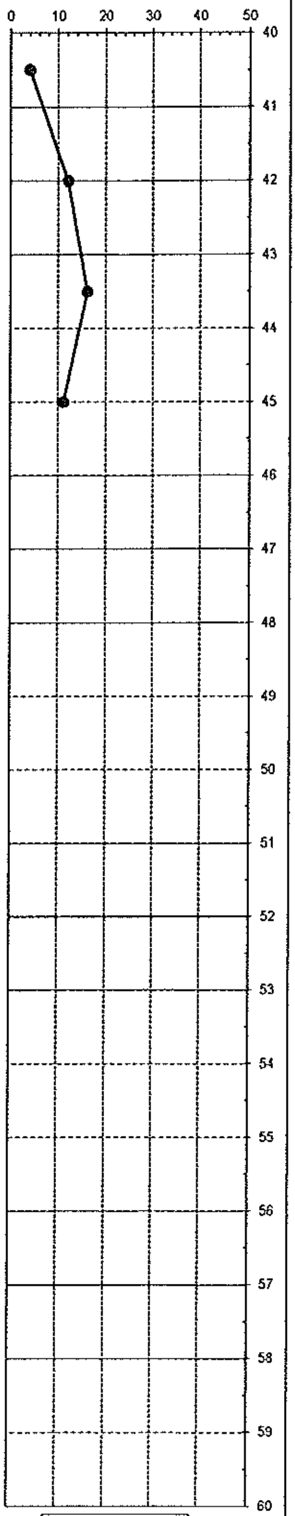

SPT PLOT

Legend:

- D — Disturbed Sample
- P — Standard Penetration Test
- UD — Undisturbed Sample
- MZ — Mazier Sample
- VS — Vane Shear Test
- C — Rock Coring
- W — Water Sample
- N - No. of Blows/300mm

NOTE:

** Existing ground level

Example:

50 | 120 = 50 Blows/120 mm

Cohesive Soil (N): 0, 2, 4, 8, 15, 30 — V.Soft, Soft, Firm, Stiff, V.Stiff, Hard

Non-cohesive Soil (N): 0, 4, 10, 30, 50 — V.Loose, Loose, Med Dense, Dense, V.Dense

HSD ENGINEERING SERVICES

ENGINEERING BOREHOLE LOG

Project :	SOIL INVESTIGATION WORKS	Borehole No :	BH 18
Location :	BAGAN DATUK WATER CITY PHASE 1, PERAK DARUL RIDZUAN	Ground Level:	0.096 m

Client :	PERBADANAN KEMAJUAN NEGERI PERAK	Rig Type :	YWE D-90R	Driller :	SHAH	Water Level :	0.90 m
Consultant:	INFRA TECH GEO SOULTIONS (M) SDN. BHD.	Drill Method:	Rotary Wash	Supervisor:	REOZA	Date Start :	17.05.2017
Maincon :	INFRA TECH PROJECTS MALAYSIA SDN. BHD.	Casing Type :	NW			Date Finish :	22.05.2017

Depth m	Strata Thick-ness	Description of Strata	Log	Sampling Detail: Sample No	Sampling Detail: Depth m	Rec Ratio	Penetration, P (mm) / SPT Blow Count: 75	75	75	75	75	75	For <75	N Value	For <300
0 – 0.30	0.30	Top soil.		D	0.300										
1.5		Very soft medium grey CLAY.		P1/D1	1.500<br>1.950	100%	1	0	0	0	1	0		1	
3		Very soft medium grey CLAY with traces of gravels.		P2/D2	3.000<br>3.450	100%	0	0	0	0	1	0		1	
4.5		Very soft medium grey CLAY with traces of gravels.		P3/D3	4.500<br>4.950	100%	0	0	0	0	0	0		0	
6		Very soft medium grey CLAY.		P4/D4	6.000<br>6.450	100%	0	0	0	0	0	0		0	
7.5		Very soft medium grey CLAY.		P5/D5	7.500<br>7.950	100%	0	0	0	0	0	0		0	
9		Very soft medium grey CLAY with traces of gravels.		P6/D6	9.000<br>9.450	100%	0	0	0	0	0	0		0	
9.5		Medium grey CLAY of extremely high plasticity.		UD 1	9.500<br>10.000	100%									
10.5		Very soft medium grey CLAY.		P7/D7	10.500<br>10.950	100%	0	0	0	0	0	0		0	
11		Medium grey CLAY of extremely high plasticity.		UD 2	11.000<br>11.500	100%									
12		Very soft medium grey CLAY.		P8/D8	12.000<br>12.450	100%	0	0	0	0	0	0		0	
12.5		Medium grey CLAY of very high plasticity.		UD 3	12.500<br>13.000	100%									
13.5		Very soft medium grey CLAY.		P9/D9	13.500<br>13.950	100%	0	0	0	0	0	0		0	
15		Very soft dark grey CLAY.		P10/D10	15.000<br>15.450	100%	0	0	0	0	0	0		0	
16.5		Soft dark grey CLAY.		P11/D11	16.500<br>16.950	100%	1	1	0	1	1	1		3	
18		Soft to firm dark grey CLAY.		P12/D12	18.000<br>18.450	100%	2	2	1	1	1	1		4	
19.5 – 20		Soft to firm dark grey CLAY.		P13/D13	19.500<br>19.950	100%	1	2	1	1	1	1		4	

Rec = Recovery

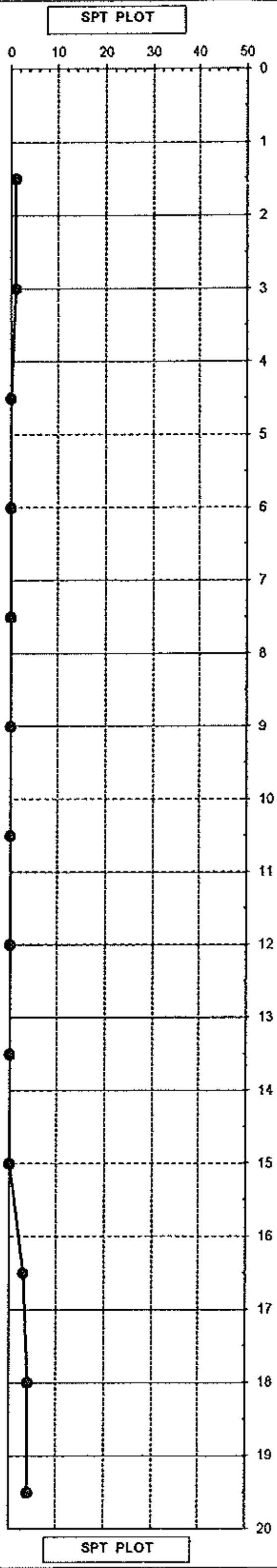

Legend:

- D – Disturbed Sample
- P – Standard Penetration Test
- UD – Undisturbed Sample
- MZ – Mazier Sample
- VS – Vane Shear Test
- C – Rock Coring
- W – Water Sample
- N - No. of Blows/300mm

NOTE:

** Existing ground level

Example:

50 | 120 = 50 Blows/120 mm

Cohesive Soil (N)	0	2	4	8	15	30	
	V.Soft,	Soft,	Firm	Stiff,	V.Stiff,	Hard	
Non-cohesive Soil (N)	0	4	10	30	50		
	V.Loose	Loose	Med Dense	Dense	V.Dense		

HSD ENGINEERING SERVICES

ENGINEERING BOREHOLE LOG

Project :	SOIL INVESTIGATION WORKS
Location :	BAGAN DATUK WATER CITY PHASE 1, PERAK DARUL RIDZUAN
Client :	PERBADANAN KEMAJUAN NEGERI PERAK
Consultant:	INFRA TECH GEO SOULTIONS (M) SDN. BHD.
Maincon :	INFRA TECH PROJECTS MALAYSIA SDN. BHD.
Rig Type :	YWE D-90R
Drill Method:	Rotary Wash
Casing Type :	NW
Driller :	SHAH
Supervisor:	REDZA
Borehole No :	BH 18
Ground Level:	0.096 m
Water Level :	0.90 m
Date Start :	17.05.2017
Date Finish :	22.05.2017

Depth m	Strata Thickness	Description of Strata	Sample No	Depth m	Rec Ratio	75	75	75	75	75	75	For <75	N Value	For <300
21		Soft dark grey sandy CLAY of intermediate plasticity.	P14/D14	21.000<br>21.450	100%	1	1	1	0	1	1		3	
22	24.00	Very soft to soft dark grey sandy CLAY.	P15/D15	22.500<br>22.950	100%	2	1	0	0	1	1		2	
24		Firm dark grey CLAY.	P16/D16	24.000<br>24.450	100%	1	2	1	1	2	1		5	
25		Soft to firm dark grey CLAY.	P17/D17	25.500<br>25.950	100%	1	2	1	1	1	1		4	
27		Firm dark grey CLAY.	P18/D18	27.000<br>27.450	100%	2	2	1	1	2	1		5	
28		Firm dark grey CLAY.	P19/D19	28.500<br>28.950	100%	1	2	1	1	2	1		5	
30	31.50	Firm dark grey spotted with black CLAY.	P20/D20	30.000<br>30.450	100%	1	0	0	0	0	1		1	
31.5	33.00	Very soft dark grey silty SAND.	P21/D21	31.500<br>31.950	100%	0	0	0	0	0	0		0	
33		Very soft dark grey CLAY.	P22/D22	33.000<br>33.450	100%	0	0	0	0	0	0		0	
34		Very soft dark grey CLAY.	P23/D23	34.500<br>34.950	100%	0	0	0	0	0	0		0	
36		Very soft dark grey CLAY.	P24/D24	36.000<br>36.450	100%	0	0	0	0	0	0		0	
37	39.00	Very soft dark grey CLAY.	P25/D25	37.500<br>37.950	100%	0	0	0	0	0	0		0	
39		Very soft dark grey sandy CLAY.	P26/D26	39.000<br>39.450	100%	0	0	0	0	0	0		0	

Rec = Recovery

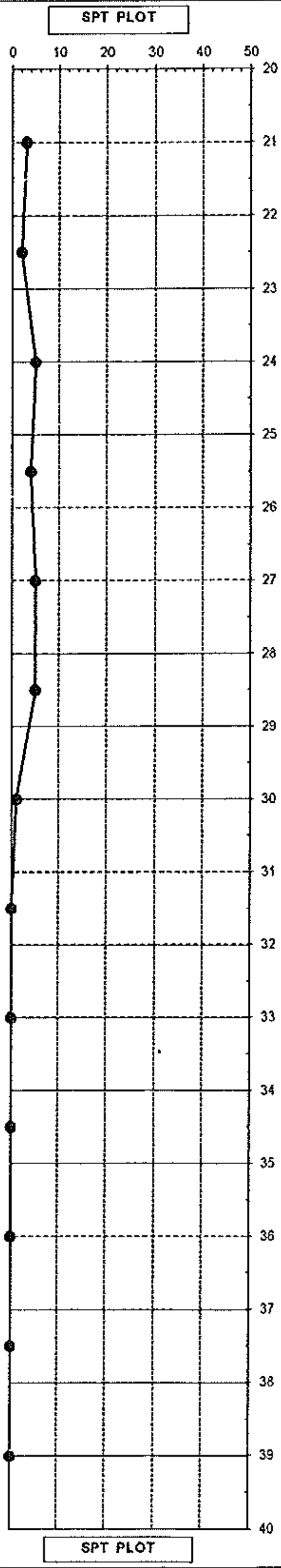

Legend:

- D – Disturbed Sample
- P – Standard Penetration Test
- UD – Undisturbed Sample
- MZ – Mazier Sample
- VS – Vane Shear Test
- C – Rock Coring
- W – Water Sample
- N - No. of Blows/300mm

NOTE:

** Existing ground level

Example:

50 | 120 = 50 Blows/120 mm

Cohesive Soil (N): 0, 2, 4, 8, 15, 30 — V.Soft, Soft, Firm, Stiff, V.Stiff, Hard

Non-cohesive Soil (N): 0, 4, 10, 30, 50 — V.Loose, Loose, Med Dense, Dense, V.Dense

HSD ENGINEERING SERVICES

ENGINEERING BOREHOLE LOG

Project : SOIL INVESTIGATION WORKS			Borehole No : BH 18
Location : BAGAN DATUK WATER CITY PHASE 1, PERAK DARUL RIDZUAN			Ground Level: 0.096 m
Client : PERBADANAN KEMAJUAN NEGERI PERAK	Rig Type : YWE D-90R	Driller : SHAH	Water Level : 0.90 m
Consultant: INFRA TECH GEO SOULTIONS (M) SDN. BHD.	Drill Method: Rotary Wash	Supervisor: REDZA	Date Start : 17.05.2017
Maincon : INFRA TECH PROJECTS MALAYSIA SDN. BHD.	Casing Type : NW		Date Finish : 22.05.2017

Depth m	Strata Thick-ness	Description of Strata	Log	SAMPLING DETAIL Sample No	Depth m	Rec Ratio	Penetration, P (mm) 75	75	75	75	75	75	For <75	N Value	For <300
							SPT BLOW COUNT								
40															
		Very soft dark grey CLAY.	- - -	P27/D27	40.500 40.950	100%	0	0	0	0	0	0		0	
41															
42		Very soft dark grey CLAY.	- - -	P28/D28	42.000 42.450	100%	1	0	1	0	0	0		1	
43															
		Very soft dark grey CLAY.	- - -	P29/D29	43.500 43.950	1%	0	0	0	1	0	0		1	
44															
45	45.45	Very soft to soft dark grey CLAY.	- - -	P30/D30	45.000 45.450	100%	1	0	1	0	0	1		2	
46		End of BH18 at 45.45 m depth													
47															
48															
49															
50															
51															
52															
53															
54															
55															
56															
57															
58															
59															
60						Rec = Recovery									

SPT PLOT

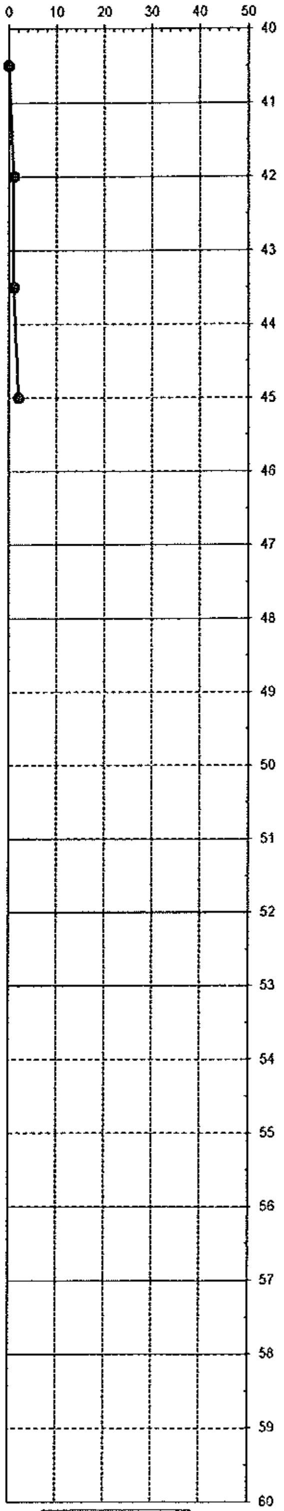

SPT PLOT

Legend:

- D — Disturbed Sample
- P — Standard Penetration Test
- UD — Undisturbed Sample
- MZ — Mazier Sample
- VS — Vane Shear Test
- C — Rock Coring
- W — Water Sample
- N - No. of Blows/300mm

NOTE:

** Existing ground level

Example:

50 | 120 = 50 Blows/120 mm

Cohesive Soil (N)	0	2	4	8	15	30
	V.Soft,	Soft,	Firm,	Stiff,	V.Stiff,	Hard

Non cohesive Soil (N)	0	4	10	30	50
	V.Loose	Loose	Med Dense	Dense	V.Dense

HSD ENGINEERING SERVICES

ENGINEERING BOREHOLE LOG

Project : SOIL INVESTIGATION WORKS			Borehole No : BH 19
Location : BAGAN DATUK WATER CITY PHASE 1, PERAK DARUL RIDZUAN			Ground Level: -0.043 m
Client : PERBADANAN KEMAJUAN NEGERI PERAK	Rig Type : YWE D-90R	Driller : SHAH	Water Level : 0.70 m
Consultant: INFRA TECH GEO SOULTIONS (M) SDN. BHD.	Drill Method: Rotary Wash	Supervisor: REDZA	Date Start : 14.06.2017
Maincon : INFRA TECH PROJECTS MALAYSIA SDN. BHD.	Casing Type : NW		Date Finish : 19.06.2017

Depth m	Strata Thick-ness	Description of Strata	Log	Sample No	Depth m	Rec Ratio	75	75	75	75	75	75	For <75	N Value	For <300
0	0.30	Top soil.		D1	0.300										
1 – 2		Very soft yellowish grey CLAY.		P1/D2	1.500<br>1.950	100%	0	0	0	0	0	0		0	
3 – 4	4.50	Very soft dark grey CLAY.		P2/D3	3.000<br>3.450	100%	0	0	0	0	0	0		0	
5	5.00	Medium dense dark grey clayey SAND.		P3/D4	4.500<br>4.950	100%	2	1	2	3	3	4		12	
	6.00	Dark grey CLAY of extremely plasticity.		UD 1	5.000<br>5.500	100%									
6 – 7		Loose dark grey clayey SAND.		P4/D5	6.000<br>6.450	100%	1	2	2	2	1	2		7	
8		Loose dark grey clayey SAND.		P5/D6	7.500<br>7.950	100%	1	2	2	2	1	2		7	
9 – 10	10.50	Loose dark grey clayey SAND.		P6/D7	9.000<br>9.450	100%	1	2	1	2	2	1		6	
11		Very soft dark grey CLAY.		P7/D8	10.500<br>10.950	100%	0	0	0	0	0	0		0	
		Dark grey CLAY of extremely plasticity.		UD 2	11.000<br>11.500	100%									
12 – 13		Very soft light grey CLAY.		P8/D9	12.000<br>12.450	100%	0	0	0	0	0	0		0	
14		Very soft medium grey CLAY.		P9/D10	13.500<br>13.950	100%	0	0	0	0	0	0		0	
15		Very soft medium grey sandy CLAY.		P10/D11	15.000<br>15.450	100%	0	0	0	0	0	0		0	
16		Dark grey CLAY of extremely plasticity.		UD 3	15.500<br>16.000	100%									
17		Very soft medium grey CLAY.		P11/D12	16.500<br>16.950	100%	0	0	0	0	0	0		0	
18 – 19		Very soft medium grey CLAY.		P12/D13	18.000<br>18.450	100%	0	0	0	0	0	0		0	
20		Very soft medium grey CLAY with traces of decayed wood.		P13/D14	19.500<br>19.950	100%	0	0	0	0	0	0		0	

Rec = Recovery

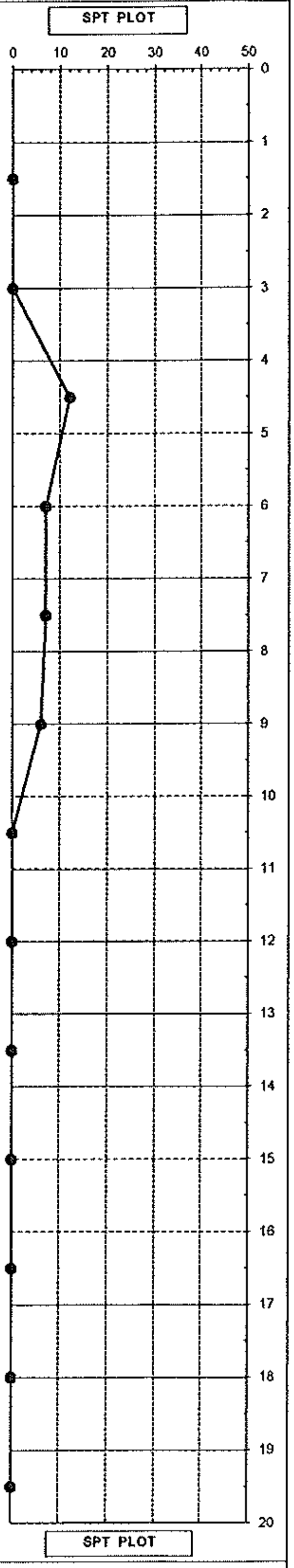

Legend:

- D – Disturbed Sample
- P – Standard Penetration Test
- UD – Undisturbed Sample
- MZ – Mazier Sample
- VS – Vane Shear Test
- C – Rock Coring
- W – Water Sample
- N - No. of Blows/300mm

NOTE:

** Existing ground level

Example:

50 | 120 = 50 Blows/120 mm

Cohesive Soil (N)	0	2	4	8	15	30
	V.Soft	Soft	Firm	Stiff	V.Stiff	Hard
Non-cohesive Soil (N)	0	4	10	30	50	
	V.Loose	Loose	Med Dense	Dense	V.Dense	

HSD ENGINEERING SERVICES

ENGINEERING BOREHOLE LOG

Project :	SOIL INVESTIGATION WORKS	Borehole No :	BH 19
Location :	BAGAN DATUK WATER CITY PHASE 1, PERAK DARUL RIDZUAN	Ground Level:	-0.043 m
Client :	PERBADANAN KEMAJUAN NEGERI PERAK / Rig Type : YWE D-90R / Driller : SHAH	Water Level :	0.70 m
Consultant:	INFRA TECH GEO SOULTIONS (M) SDN. BHD. / Drill Method: Rotary Wash / Supervisor: REDZA	Date Start :	14.06.2017
Maincon :	INFRA TECH PROJECTS MALAYSIA SDN. BHD. / Casing Type : NW	Date Finish :	19.06.2017

Depth m	Strata Thickness	Description of Strata	Log	Sample No	Type	Depth m	Rec Ratio	75	75	75	75	75	75	For <75	N Value	For <300
				SAMPLING DETAIL				Penetration, P (mm) / SPT BLOW COUNT								
20		Dark grey CLAY of extremely plasticity.		UD 4	UD	20.000 / 20.500	100%									
21		Very soft dark grey CLAY with traces of decayed wood.		P14/D15	P	21.000 / 21.450	100%	1	0	0	1	1	0		2	
22		Very soft dark grey CLAY with traces of decayed wood.		P15/D16	P	22.500 / 22.950	100%	1	1	0	0	1	1		2	
24		Very soft dark grey CLAY.		P16/D17	P	24.000 / 24.450	100%	0	0	0	0	0	0		0	
25		Very soft dark grey CLAY.		P17/D18	P	25.500 / 25.950	100%	0	0	0	0	0	0		0	
27	27.00	No recovery.		P18	P	27.000 / 27.450	0%	1	1	1	0	1	1		3	
28	28.50	Soft to firm dark grey CLAY.		P19/D19	P	28.500 / 28.950	100%	1	0	1	1	1	1		4	
30		Firm dark grey CLAY.		P20/D20	P	30.000 / 30.450	100%	1	2	1	1	2	1		5	
31		Firm medium grey CLAY.		P21/D21	P	31.500 / 31.950	100%	1	1	1	1	1	1		4	
33		Soft to firm medium grey CLAY.		P22/D22	P	33.000 / 33.450	100%	1	1	1	0	1	2		4	
34		Firm medium grey CLAY.		P23/D23	P	34.500 / 34.950	100%	1	2	1	1	2	2		6	
36		Firm medium brown streaked with pale green CLAY.		P24/D24	P	36.000 / 36.450	100%	1	2	2	2	1	2		7	
37		Firm medium brown CLAY.		P25/D25	P	37.500 / 37.950	100%	1	2	2	2	1	2		7	
39		Firm light brown streaked with medium brown CLAY.		P26/D26	P	39.000 / 39.450	100%	1	2	2	1	2	2		7	
40							Rec = Recovery									

SPT PLOT

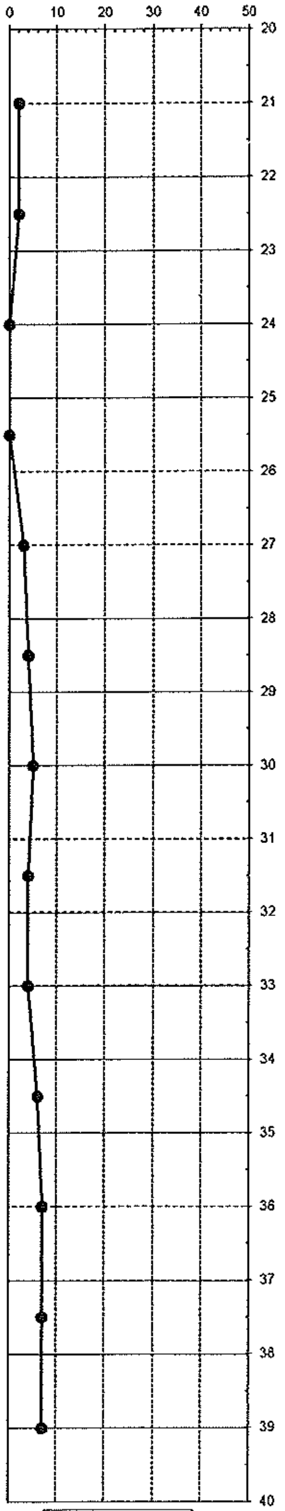

SPT PLOT

Legend:

- D – Disturbed Sample
- P – Standard Penetration Test
- UD – Undisturbed Sample
- MZ – Mazier Sample
- VS – Vane Shear Test
- C – Rock Coring
- W – Water Sample
- N - No. of Blows/300mm

NOTE:

** Existing ground level

Example:

50 | 120 = 50 Blows/120 mm

Cohesive Soil (N): 0, 2, 4, 8, 15, 30 — V.Soft, Soft, Firm, Stiff, V.Stiff, Hard

Non-cohesive Soil (N): 0, 4, 10, 30, 50 — V.Loose, Loose, Med Dense, Dense, V.Dense

HSD ENGINEERING SERVICES

ENGINEERING BOREHOLE LOG

Project : SOIL INVESTIGATION WORKS			Borehole No : BH 19
Location : BAGAN DATUK WATER CITY PHASE 1, PERAK DARUL RIDZUAN			Ground Level: -0.043 m
Client : PERBADANAN KEMAJUAN NEGERI PERAK	Rig Type : YWE D-90R	Driller : SHAH	Water Level : 0.70 m
Consultant: INFRA TECH GEO SOULTIONS (M) SDN. BHD.	Drill Method: Rotary Wash	Supervisor: REDZA	Date Start : 14.06.2017
Maincon : INFRA TECH PROJECTS MALAYSIA SDN. BHD.	Casing Type : NW		Date Finish : 19.06.2017

Depth m	Strata Thickness	Description of Strata	Log	Sample No	Depth m	Rec Ratio	75	75	75	75	75	75	For <75	N Value	For <300
				SAMPLING DETAIL			Penetration, P (mm) / SPT BLOW COUNT							N	
40 – 42	42.00	Medium dense light brown clayey SAND.		P27/D27	40.500<br>40.950	100%	6	5	5	9	7	5		26	
42 – 43		Stiff medium grey CLAY.		P28/D28	42.000<br>42.450	100%	1	2	4	3	2	2		11	
43 – 44.5		Firm medium grey CLAY.		P29/D29	43.500<br>43.950	100%	1	2	3	1	2	1		7	
45 – 45.45	45.45	Firm to stiff dark grey CLAY.		P30/D30	45.000<br>45.450	100%	2	3	2	2	2	2		8	
46		End of BH 19 at 45.45 m depth.													

Depth scale: 40, 41, 42, 43, 44, 45, 46, 47, 48, 49, 50, 51, 52, 53, 54, 55, 56, 57, 58, 59, 60

Rec = Recovery

SPT PLOT

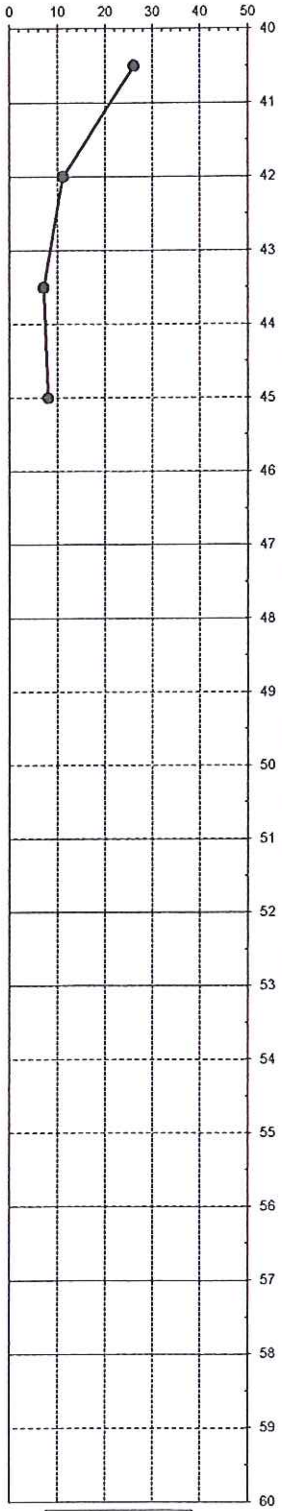

SPT PLOT

Legend:

- D – Disturbed Sample
- P – Standard Penetration Test
- UD – Undisturbed Sample
- MZ – Mazier Sample
- VS – Vane Shear Test
- C – Rock Coring
- W – Water Sample
- N - No. of Blows/300mm

NOTE:

** Existing ground level

Example:

50 | 120 = 50 Blows/120 mm

Cohesive Soil (N): 0, 2, 4, 8, 15, 30 — V.Soft, Soft, Firm, Stiff, V.Stiff, Hard

Non-cohesive Soil (N): 0, 4, 10, 30, 50 — V.Loose, Loose, Med Dense, Dense, V.Dense

HSD ENGINEERING SERVICES

ENGINEERING BOREHOLE LOG

Project :	SOIL INVESTIGATION WORKS	Borehole No :	BH 20
Location :	BAGAN DATUK WATER CITY PHASE 1, PERAK DARUL RIDZUAN	Ground Level:	-0.019 m

Client :	PERBADANAN KEMAJUAN NEGERI PERAK	Rig Type :	YWE D-90R	Driller :	SHAH	Water Level :	
Consultant:	INFRA TECH GEO SOULTIONS (M) SDN. BHD.	Drill Method:	Rotary Wash	Supervisor:	REDZA	Date Start :	12.07.2017
Maincon :	INFRA TECH PROJECTS MALAYSIA SDN. BHD.	Casing Type :	NW			Date Finish :	15.07.2017

Strata Thickness	Description of Strata	Sample No	Depth m	Rec Ratio	75	75	75	75	75	75	For <75	N Value	For <300
0.30	Top soil.	D	0.300										
3.00	Very soft dark grey CLAY with some decayed wood.	P1/D1	1.500<br>1.950	100%	0	0	0	0	0	0		0	
4.50	No recovery.	P2	3.000<br>3.450	0%	0	0	0	0	0	0		0	
		VS 1	3.500										
	Very soft dark grey CLAY with some seashell.	P3/D2	4.500<br>4.950	100%	0	0	0	0	0	0		0	
	Dark grey CLAY of extremely high plasticity.	UD 1	5.000<br>5.500	100%									
	Very soft dark grey CLAY with some seashell.	P4/D3	6.000<br>6.450	100%	0	0	0	0	0	0		0	
		VS 2	6.500										
	Very soft dark grey CLAY with lenses of fine sand and some seashell.	P5/D4	7.500<br>7.950	100%	0	0	0	0	0	0		0	
	Very soft dark grey CLAY with some seashell.	P6/D5	9.000<br>9.450	100%	0	0	0	0	0	0		0	
		VS 3	9.500										
	Very soft dark grey CLAY with some seashell.	P7/D6	10.500<br>10.950	100%	0	0	0	0	0	0		0	
	Dark grey CLAY of extremely high plasticity.	UD 2	11.000<br>11.500	100%									
	Very soft dark grey CLAY with lenses of fine sand and some seashell.	P8/D7	12.000<br>12.450	100%	0	0	0	0	0	0		0	
		VS 4	12.500										
14.00	Very soft dark grey CLAY with fine sand, seashell and decayed wood.	P9/D8	13.500<br>13.950	100%	0	0	0	0	0	0		0	
	Dark grey sandy CLAY of intermediate plasticity.	UD 3	14.000<br>14.500	100%									
16.50	Very soft dark grey sandy CLAY with some seashell and decayed wood.	P10/D9	15.000<br>15.450	100%	0	0	0	0	0	0		0	
	Very soft dark grey CLAY with lenses of fine sand and some seashell and decayed wood.	P11/D10	16.500<br>16.950	100%	0	0	0	0	0	0		0	
	Very soft dark grey CLAY with lenses of fine sand and some seashell and decayed wood.	P12/D11	18.000<br>18.450	100%	0	0	0	1	0	1		2	
19.50		VS 5	18.500										
	Very soft dark grey sandy CLAY with decayed wood and seashell.	P13/D12	19.500<br>19.950	100%	0	0	0	0	0	0		0	

Rec = Recovery

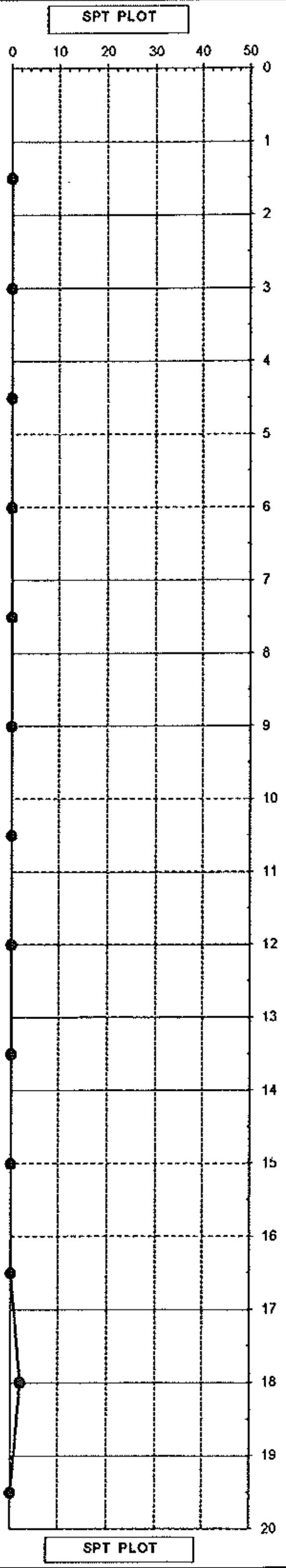

Legend:

- D — Disturbed Sample
- P — Standard Penetration Test
- UD — Undisturbed Sample
- MZ — Mazier Sample
- VS — Vane Shear Test
- C — Rock Coring
- W — Water Sample
- N - No. of Blows/300mm

NOTE:

** Existing ground level

Example:

50 | 120 = 50 Blows/120 mm

Cohesive Soil (N)	0	2	4	8	15	30	
	V.Soft,	Soft,	Firm,	Stiff,	V.Stiff,	Hard	
Non-cohesive Soil (N)	0	4	10	30	50		
	V.Loose	Loose	Med Dense	Dense	V.Dense		

HSD ENGINEERING SERVICES

ENGINEERING BOREHOLE LOG

Project :	SOIL INVESTIGATION WORKS	Borehole No :	BH 20
Location :	BAGAN DATUK WATER CITY PHASE 1, PERAK DARUL RIDZUAN	Ground Level:	-0.019 m
Client :	PERBADANAN KEMAJUAN NEGERI PERAK	Water Level :	0.00 m
Consultant:	INFRA TECH GEO SOULTIONS (M) SDN. BHD.	Date Start :	12.07.2017
Maincon :	INFRA TECH PROJECTS MALAYSIA SDN. BHD.	Date Finish :	15.07.2017

Rig Type :	YWE D-90R	Driller :	SHAH
Drill Method:	Rotary Wash	Supervisor:	REDZA
Casing Type :	NW		

Depth m	Strata Thickness	Description of Strata	Log	Sample No	Depth m	Rec Ratio	75	75	75	75	75	75	For <75	N Value	For <300
20		Dark grey CLAY of high plasticity.		UD 4	20.000 20.500										
21		Very soft dark grey CLAY with lenses of fine sand with some decayed wood and seashell.		P14/D13	21.000 21.450	100%	0	0	0	0	0	0		0	
22		Very soft dark grey CLAY with decayed wood.		P15/D14	22.500 22.950	100%	0	0	0	0	0	0		0	
24		Very soft dark grey CLAY with decayed wood.		P16/D15	24.000 24.450	100%	0	0	0	0	0	0		0	
25		Very soft dark grey CLAY with decayed wood.		P17/D16	25.500 25.950	100%	0	0	0	0	0	0		0	
27		Very soft dark grey CLAY with decayed wood and seashell.		P18/D17	27.000 27.450	100%	0	0	0	0	0	0		0	
28		Soft dark grey CLAY with decayed wood.		P19/D18	28.500 28.950	78%	0	0	0	1	1	1		3	
30		Soft dark grey CLAY with decayed wood.		P20/D19	30.000 30.450	89%	0	0	0	0	0	0		0	
31		Soft dark grey CLAY with decayed wood.		P21/D20	31.500 31.950	100%	0	0	0	0	0	0		0	
33		Soft dark grey CLAY with lenses of fine grained sand and decayed wood.		P22/D21	33.000 33.450	100%	0	0	0	0	0	0		0	
34	34.50	Medium dense dark grey fine to medium coarse grained SAND with decayed wood.		P23/D20	34.500 34.950	20%	1	4	5	6	6	5		22	
36		Medium dense grey fine to medium coarse grained SAND.		P24/D23	36.000 36.450	42%	0	4	4	5	8	7		24	
37	37.50	Stiff dark grey sandy CLAY with decayed wood.		P25/D24	37.500 37.950	61%	1	2	4	3	2	3		12	
39	39.00	Firm lght grey CLAY with lenses of fine sand and decayed wood.		P26/D25	39.000 39.450	100%	0	1	1	1	2	2		6	
40															

Sampling Detail; Penetration, P (mm); SPT Blow Count. Rec = Recovery

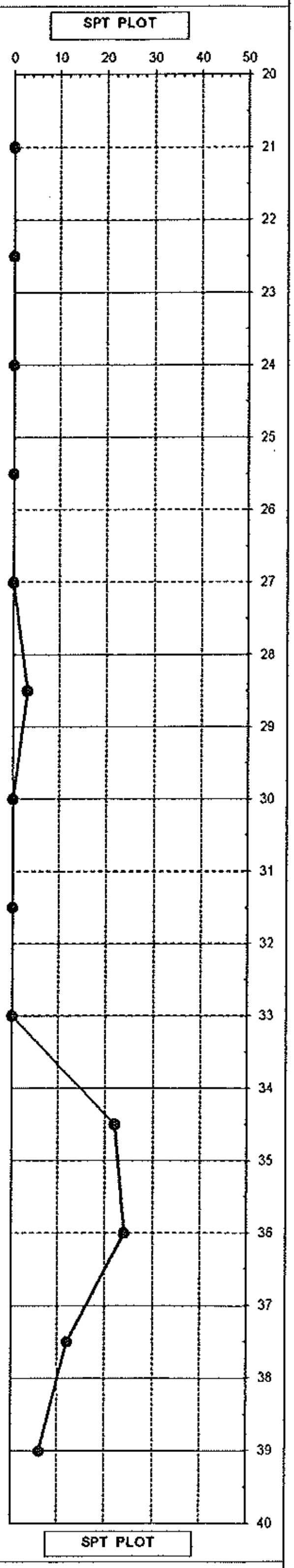

Legend:

- D — Disturbed Sample
- P — Standard Penetration Test
- UD — Undisturbed Sample
- MZ — Mazier Sample
- VS — Vane Shear Test
- C — Rock Coring
- W — Water Sample
- N - No. of Blows/300mm

NOTE:

** Existing ground level

Example:

50 | 120 = 50 Blows/120 mm

Cohesive Soil (N): 0, 2, 4, 8, 15, 30 — V.Soft, Soft, Firm, Stiff, V.Stiff, Hard

Non-cohesive Soil (N): 0, 4, 10, 30, 50 — V.Loose, Loose, Med Dense, Dense, V.Dense

HSD ENGINEERING SERVICES

ENGINEERING BOREHOLE LOG

Project : SOIL INVESTIGATION WORKS
Location : BAGAN DATUK WATER CITY PHASE 1, PERAK DARUL RIDZUAN
Client : PERBADANAN KEMAJUAN NEGERI PERAK
Consultant: INFRA TECH GEO SOULTIONS (M) SDN. BHD.
Maincon : INFRA TECH PROJECTS MALAYSIA SDN. BHD.

Rig Type : YWE D-90R
Drill Method: Rotary Wash
Casing Type : NW

Driller : SHAH
Supervisor: REDZA

Borehole No : BH 20
Ground Level: -0.019 m
Water Level : 0.00 m
Date Start : 12.07.2017
Date Finish : 15.07.2017

Depth m	Strata Thickness	Description of Strata	Log	Sample No	Depth m	Rec Ratio	75	75	75	75	75	75	For <75	N Value	For <300
40–42	42.00	Very soft dark grey CLAY with some decayed wood and seashell.		P27/D26	40.500 40.950	100%	0	0	0	0	0	0		0	
42–43.5	43.50	Soft light grey sandy CLAY.		P28/D27	42.000 42.450	100%	3	3	3	0	0	0		3	
43.5–45		Dense light grey medium to coarse SAND.		P29/D28	43.500 43.950	89%	3	2	9	9	8	7		33	
45–45.45	45.45	Medium dense light grey fine to medium coarse SAND.		P30/D29	45.000 45.450	42%	0	3	5	8	8	7		28	
		End of BH 20 at 45.45 m depth.													
		Standpipe installed at 18.00 m depth.													

SAMPLING DETAIL; Penetration, P (mm) — SPT BLOW COUNT; Rec = Recovery

SPT PLOT

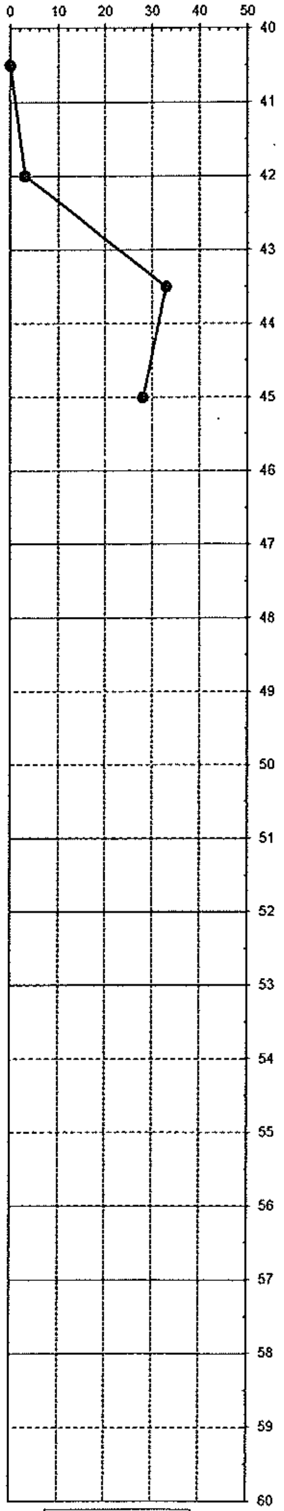

SPT PLOT

Legend:

- D — Disturbed Sample
- P — Standard Penetration Test
- UD — Undisturbed Sample
- MZ — Mazier Sample
- VS — Vane Shear Test
- C — Rock Coring
- W — Water Sample
- N - No. of Blows/300mm

NOTE:

** Existing ground level

Example:

50 | 120 = 50 Blows/120 mm

Cohesive Soil (N): 0, 2, 4, 8, 15, 30 — V.Soft, Soft, Firm, Stiff, V.Stiff, Hard

Non-cohesive Soil (N): 0, 4, 10, 30, 50 — V.Loose, Loose, Med Dense, Dense, V.Dense

HSD ENGINEERING SERVICES
